# Global chromatin landscapes identify candidate noncoding modifiers of cardiac rhythm

**DOI:** 10.1172/JCI153635

**Published:** 2023-02-01

**Authors:** Samadrita Bhattacharyya, Rahul K. Kollipara, Gabriela Orquera-Tornakian, Sean Goetsch, Minzhe Zhang, Cameron Perry, Boxun Li, John M. Shelton, Minoti Bhakta, Jialei Duan, Yang Xie, Guanghua Xiao, Bret M. Evers, Gary C. Hon, Ralf Kittler, Nikhil V. Munshi

**Affiliations:** 1 Department of Internal Medicine, Division of Cardiology,; 2 McDermott Center for Human Growth and Development,; 3 Quantitative Biomedical Research Center, Department of Population and Data Sciences,; 4 Laboratory of Regulatory Genomics, Cecil H. and Ida Green Center for Reproductive Biology Sciences, Division of Basic Reproductive Biology Research, Department of Obstetrics and Gynecology,; 5 Department of Bioinformatics,; 6 Hamon Center for Regenerative Science and Medicine, and; 7 Department of Molecular Biology, UT Southwestern Medical Center, Dallas, Texas, USA.

**Keywords:** Cardiology, Cardiovascular disease, Epigenetics, Transcription

## Abstract

Comprehensive *cis-*regulatory landscapes are essential for accurate enhancer prediction and disease variant mapping. Although *cis-*regulatory element (CRE) resources exist for most tissues and organs, many rare — yet functionally important — cell types remain overlooked. Despite representing only a small fraction of the heart’s cellular biomass, the cardiac conduction system (CCS) unfailingly coordinates every life-sustaining heartbeat. To globally profile the mouse CCS *cis-*regulatory landscape, we genetically tagged CCS component–specific nuclei for comprehensive assay for transposase-accessible chromatin–sequencing (ATAC-Seq) analysis. Thus, we established a global CCS-enriched CRE database, referred to as CCS-ATAC, as a key resource for studying CCS-wide and component-specific regulatory functions. Using transcription factor (TF) motifs to construct CCS component–specific gene regulatory networks (GRNs), we identified and independently confirmed several specific TF sub-networks. Highlighting the functional importance of CCS-ATAC, we also validated numerous CCS-enriched enhancer elements and suggested gene targets based on CCS single–cell RNA-Seq data. Furthermore, we leveraged CCS-ATAC to improve annotation of existing human variants related to cardiac rhythm and nominated a potential enhancer-target pair that was dysregulated by a specific SNP. Collectively, our results established a CCS-regulatory compendium, identified novel CCS enhancer elements, and illuminated potential functional associations between human genomic variants and CCS component–specific CREs.

## Graphical abstract

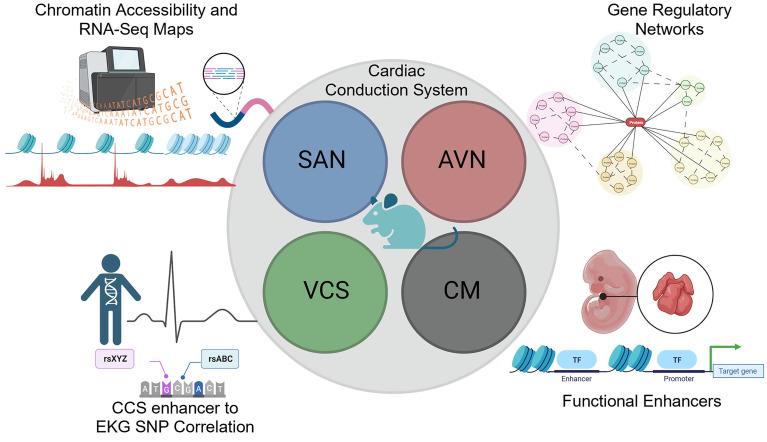

## Introduction

To ensure efficient heart contraction and proper blood circulation necessary for life, the cardiac conduction system (CCS) coordinates a fixed sequence of events. Electrical impulses are initiated in the sinoatrial node (SAN), slowed in the atrioventricular node (AVN), and rapidly transmitted through the ventricular conduction system (VCS) (1–3). Despite its essential function, the molecular underpinnings of CCS lineage–specific function and maintenance remain poorly understood. Methods for CCS identification, marking, and recombination have demonstrated that CCS cells originate from cardiomyocyte (CM) progenitors and uncovered key mechanistic insights (1–3). Interestingly, each CCS component possesses a distinct lineage history (1–3), and recent single-cell studies have uncovered additional heterogeneity within each CCS component (4, 5). In general, few transcription factors (TFs) have been shown to regulate CCS-component formation and even fewer enhancers have been validated to drive CCS-enriched gene expression (1–3). Since gene regulatory networks (GRNs) are the drivers of cellular phenotypes (6, 7), defining CCS component–specific networks represents a critical step toward a molecular understanding of CCS cell–specific function during normal cardiac rhythm and dysfunction in cardiac dysrhythmia.

The ENCyclopedia Of DNA Elements (ENCODE) consortium (8) was established to annotate the noncoding genome using a vast array of genomic assays; this has collectively defined functional enhancers, lineage-specific GRNs, and the relationships between genome-wide association study (GWAS) single nucleotide polymorphisms (SNPs) and *cis-*regulatory elements (CREs). However, existing ENCODE data sets are derived from cell lines, whole organs, and large anatomical regions (8, 9), which make them less useful for exploring under-represented cell types. For rare cells, many informative CREs are diluted out or eliminated altogether by dominant signals from more abundant cell types (6). Consequently, the lack of rare cell–type CRE compendia limits enhancer identification and accurate GWAS SNP annotation. Surmounting these hurdles has been particularly challenging in the cardiovascular system. Despite efforts to generate cardiac CRE data sets from intact tissue samples and ES cell–derived CMs (10–13), few studies have interrogated CCS subtype–specific genomic elements, and most were performed by TF Chromatin Immunoprecipitation–sequencing (ChIP-Seq) (14, 15). Nevertheless, recent studies have partially addressed these challenges by mapping chromatin accessibility in the mouse SAN (16, 17) and AVN as well as human SAN-like cells differentiated from ES cells (18). Aside from characterizing novel enhancers for *Isl1*, *Tbx3*, and *Shox2*, these studies also provided mechanistic insight into specific human genomic variants (17, 18). Nevertheless, global analysis of CCS chromatin accessibility across components derived from bona fide conduction cells in vivo has remained challenging. As a result, functional annotation of many electrocardiogram-related (EKG-related) and cardiac rhythm–related GWAS SNPs remains difficult since a comprehensive encyclopedia of CCS-enriched CREs does not yet exist. Thus, we sought to address this knowledge gap by defining a reference regulome for the CCS, which comprises only a small fraction of the heart’s total cell count.

Here we describe a comprehensive CRE database for each CCS component, altogether comprising 99,041 nonredundant ATAC-Seq peaks. Focusing on unique CCS elements, we inferred component-specific regulatory strategies that dictated how changes in chromatin accessibility influenced CCS lineage–specific function and identified candidate TFs that drove CRE usage. By aggregating CCS component–specific regulatory elements, we constructed putative GRNs for each CCS lineage. Furthermore, we intersected our CCS-enriched CRE database, CCS-ATAC, and cardiac H3K27ac mouse ENCODE data sets to identify CCS-enriched enhancers, many of which we validated with the VISTA transgenic enhancer database (19). Finally, we demonstrated that CCS-ATAC improved annotation of EKG- and cardiac rhythm–related human variants by enriching for specific GWAS SNPs. Taken together, our study establishes a CCS regulatory compendium, identifies what we believe to be novel CCS enhancer elements, and clarifies associations between human genomic variants and CCS component-specific CREs.

## Results

### CCS-ATAC: a chromatin accessibility roadmap for the CCS.

**Figure 1:**
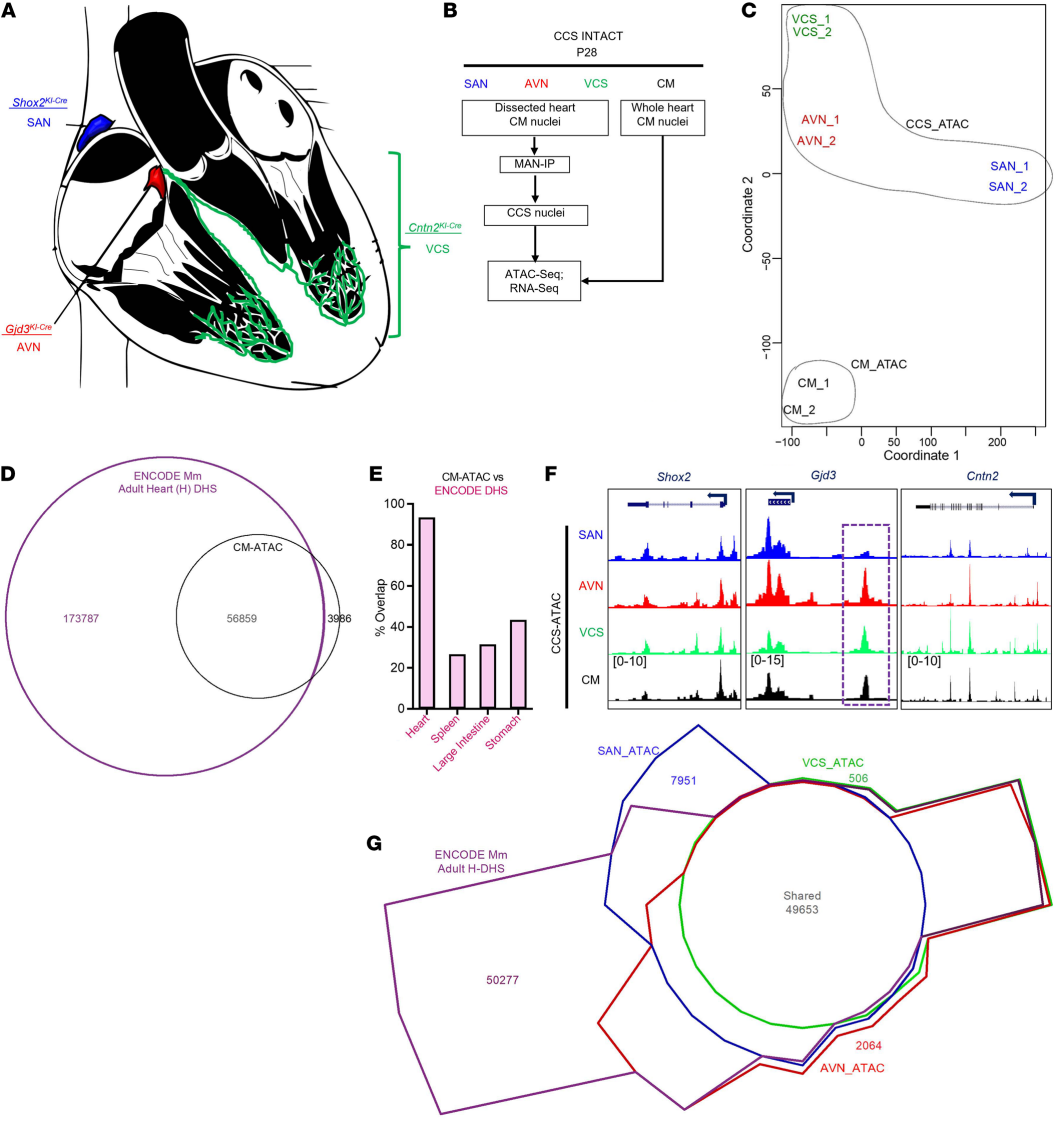
Purification of CCS component–specific nuclei to create a comprehensive regulatory atlas. (**A**) Diagram of CCS components with associated Cre driver lines. (**B**) Experimental workflow. MAN-IP, magnet-assisted nuclei immunoprecipitation. (**C**) MDS plot of individual ATAC-Seq data sets. CCS-ATAC and CM-ATAC data subsets are indicated by the outlined areas. (**D**) Venn diagram comparing CM-ATAC with ENCODE DHS-Seq data set from adult (8 weeks) mouse (Mm) heart. (**E**) Bar graph representing percentage overlap between CM-ATAC and the indicated ENCODE DHS-Seq data sets. H, heart (8 weeks); S, spleen (8 weeks); LI, large intestine (8 weeks); St, stomach (postnatal). (**F**) Genome browser tracks for *Shox2*, *Gjd3*, and *Cntn2* loci, which contain the Cre drivers used in the current study. Purple dotted box indicates the previously characterized AVN enhancer (25). (**G**) Chow-Ruskey plot comparing CCS-ATAC with ENCODE adult heart data set. Numbers of unique or shared regions are shown.

Technical challenges have hampered construction of a comprehensive CCS *cis-*regulatory map. In addition, CCS cell scarcity hinders genomic analyses requiring large quantities of starting material, such as DNase I-hypersensitivity–Seq (DHS-Seq) or ChIP-Seq. Although alternative approaches have been developed (20), substantial roadblocks remain. To address these challenges and generate a database of CCS CREs, we adapted the isolation of nuclei tagged in specific cell types (INTACT) method to purify CCS component–specific nuclei (6, 21, 22). In brief, CCS-INTACT leverages CCS component–specific Cre driver lines (4, 23, 24) with an inducible nuclear membrane tag (6) (Figure 1A). Using a published method (22), we purified CCS component-specific nuclei from the P28 mouse adult heart (Figure 1B). We chose P28 to maximize recovery of nuclei and to focus our subsequent analysis on epigenetic phenomena associated with lineage-committed CCS cell types. Even though CCS cell types constitute only a small fraction of the heart’s cellular biomass, CCS-INTACT enabled efficient purification of lineage-specific nuclei with high sensitivity and specificity (Supplemental Figure 1; supplemental material available online with this article; https://doi.org/10.1172/JCI153635DS1).

Next, we performed ATAC-Seq on purified nuclei (Figure 1B). For comparison, we profiled CM nuclei acquired from the whole heart (Figure 1B). Individual ATAC-Seq data sets mapped to the expected genomic locations with similar overall patterns (Supplemental Figure 2, A–D). Moreover, a multi-dimension scaling (MDS) plot of the CCS and CM data sets revealed high concordance between biological replicates and separation of CCS cell types from overall CMs (Figure 1C). To benchmark our approach, we compared the CM-ATAC data set with ENCODE adult heart (H) DHS-Seq data and observed a high degree of overlap (Figure 1D). We also confirmed the specificity of CM-ATAC by comparing it with ENCODE DHS data sets for adult spleen, liver, and stomach (Figure 1E). Focusing on CCS and CM marker genes (Figure 1F and Supplemental Figure 2E), we observed minimal differences in chromatin accessibility between components, although we accurately identified a previously described *Gjd3* (Cx30.2) enhancer by AVN-enriched chromatin accessibility (25). Overall, our ATAC-Seq data sets allowed retrospective identification of 5 previously described CCS-enriched enhancers (Supplemental Figure 2, F–H). Taken together, these data illustrate the fidelity and robustness of our ATAC-Seq data sets.

In parallel, we generated nuclear RNA-Seq data sets from purified nuclei with reasonable concordance between biological replicates (Supplemental Figure 3A) and good correlation for individual marker genes (Supplemental Figure 3, B and C). Differential transcript analysis identified genes that were upregulated or downregulated in each CCS component compared with CMs (Supplemental Figure 3, D–F). Gene set enrichment analysis (see Methods) of SAN-, AVN-, and VCS-enriched genes recovered biological terms consistent with their CCS component-specific function (Supplemental Figure 3, G–I). In general, we found that CCS component-specific gene expression was consistent with prior studies (26–28) (Supplemental Figure 4).

Although CM-ATAC demonstrated substantial overlap with ENCODE H-DHS-Seq data (Figure 1D), 173,787 ENCODE DHS regions remained unassigned. While some nonoverlapping regions were likely derived from nonmyocytes in the ENCODE heart samples, we hypothesized that many could reflect CCS-enriched signals. To address this latter possibility, we performed a 4-way comparison of the SAN, AVN, and VCS ATAC-Seq data with the ENCODE H-DHS data set (Figure 1G). Interestingly, only 50,277 regions remained unassigned, suggesting that a substantial fraction of regions in the original ENCODE heart data sets represented signals from CCS cell types. Moreover, we found that 7,951, 2,064, and 506 regions were unique to the CCS data sets, thereby identifying putative CCS-enriched CREs. Although most of the overlap between CCS-ATAC and ENCODE H-DHS data is captured by the adult data set, incremental improvement was observed by adding ENCODE data from E10.5 and P0 mouse heart (Supplemental Figure 5). To address whether newly identified CCS CREs overlap CREs from other tissues, we compared our ATAC-Seq data sets (135,587 nonredundant regions) with the entire mouse ENCODE DHS Universe (9), including every tissue throughout development, and found that 99.86% of ATAC-Seq regions demonstrated overlap (Supplemental Figure 6, A and B). Nearly all CCS-enriched CREs were captured by the overall ENCODE data set, suggesting potential cooption of CCS enhancers by other tissues, or vice versa (29). Interestingly, we observed 142 completely novel chromatin accessibility peaks not encountered in the ENCODE Universe (Supplemental Figure 6, B–E, and Supplemental Table 1), 33 of which were component-specific (11 SAN, 12 AVN, and 10 VCS). It is formally possible that these novel candidate elements could merely represent noise, and definitive proof of enhancer functionality will require future experimentation. Collectively, these data establish what we believe to be a novel method for isolating CCS-enriched nuclei, describe high-fidelity chromatin accessibility maps, and identify many putative and novel CCS-enriched CREs.

### Insight into global CCS cis-regulatory logic from chromatin accessibility patterns.

**Figure 2:**
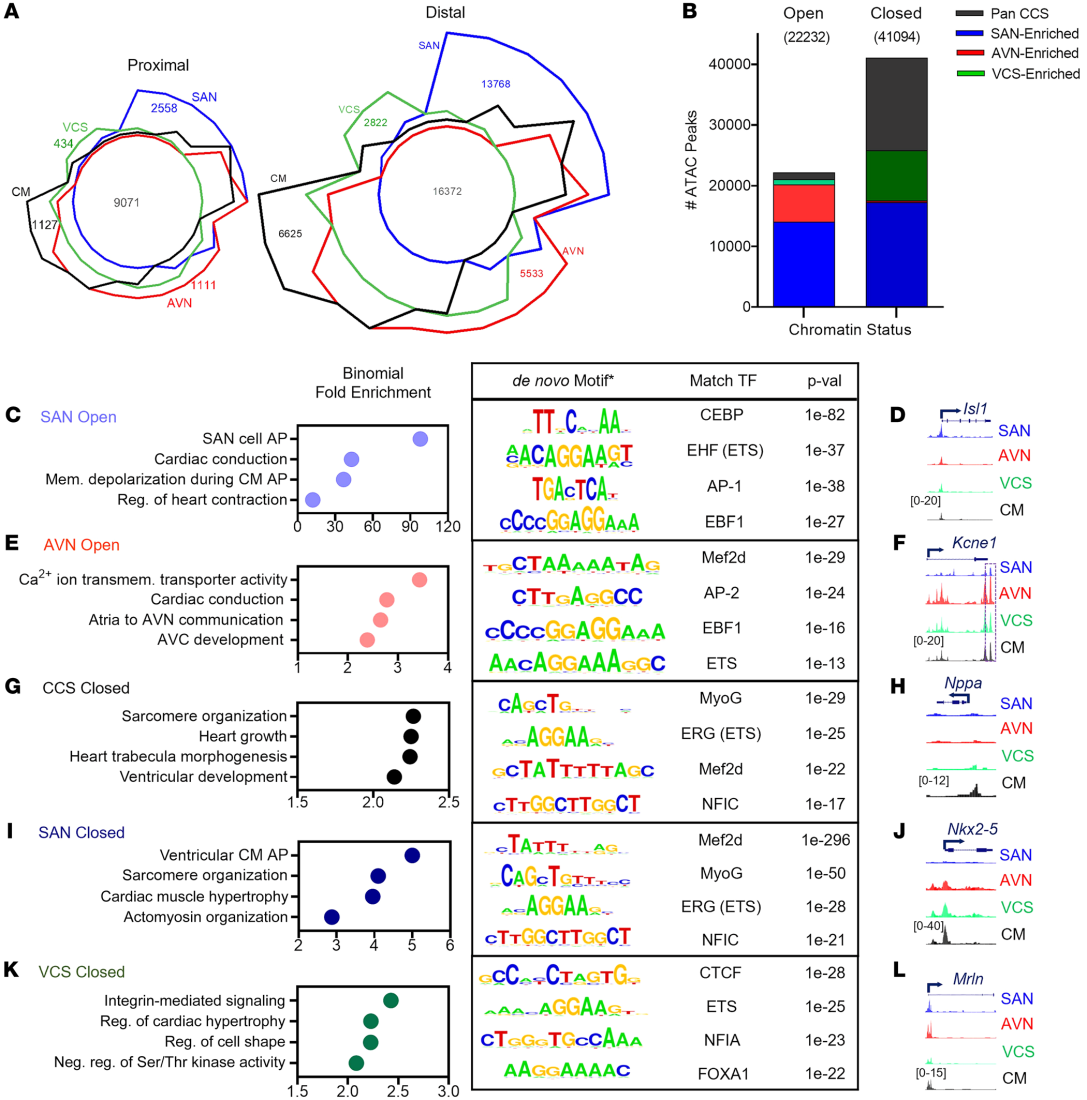
Global regulatory logic of CCS component identity. (**A**) Chow-Ruskey plot comparing CM-ATAC with CCS-ATAC components for proximal (left) and distal (right) regions identified by ATAC-Seq. Numbers of unique or shared regions are displayed. (**B**) Bar graph representing overall number of regions that are uniquely open or closed relative to the CCS. Total number of aggregated regions for CCS and individual components are stacked and color-coded within each bar. (**C**) GO term identification (left) and motif discovery (right) for SAN Open regions. (**D**) Genome browser view of the *Isl1* locus. (**E**) GO term identification (left) and motif discovery (right) for AVN Open regions. (**F**) Genome browser view of the *Kcne1* locus. Purple dotted box indicates the previously reported heart enhancer (74). (**G**) GO term identification (left) and motif discovery (right) for CCS Closed regions. (**H**) Genome browser view of the *Nppa* locus. (**I**) GO term identification (left) and motif discovery (right) for SAN Closed regions. (**J**) Genome browser view of the *Nkx2-5* locus. (**K**) GO term identification (left) and motif discovery (right) for VCS Closed regions. (**L**) Genome browser view of the *Mrln* locus. GO terms are ordered by binomial fold enrichment, and transcription factor (TF) motifs are ranked by fold-enrichment compared with the whole genome for ATAC-Seq peaks that overlapped ENCODE mouse Heart-H3K27Ac ChIP-Seq regions. AP, action potential; Mem, membrane; reg, regulation; AVC, atrioventricular canal; neg, negative.

The *cis-*regulatory logic that distinguishes conducting from working CMs and among the various CCS components remains to be completely understood (1–3). Our CCS-ATAC data set, with broad representation across the entire CCS, provided a unique opportunity to explore the underlying regulatory properties within and across CCS components. We began by grouping all ATAC-Seq peaks (proximal and distal) into the following clusters: (a) unique differentially accessible regions (DARs; Supplemental Figure 7A); (b)shared regions (Supplemental Figure 7B); and (c) mixed DARs (Supplemental Figure 7C). Although the distinction between proximal and distal peaks is arbitrarily defined, previous studies have suggested that distal elements are key drivers of cell-type specificity (30, 31). Thus, we used established cutoffs (32) to categorize regions of accessibility and performed a 4-way comparison (Figure 2A). As expected, we found that the vast majority of CCS-enriched open regions were distal, and the SAN contained, by far, the most unique regions among all CCS components (Figure 2A). Even when unique distal regions were identified by pairwise comparison with CMs (Supplemental Figure 7, D–F), SAN-enriched peaks were more numerous and distinct than AVN- or VCS-enriched peaks. Furthermore, extensive sharing of AVN- and VCS-enriched peaks was observed (Supplemental Figure 7, E and F), which is consistent with the known overlap between the distal AVN and the His bundle comprising the proximal VCS (4). Thus, these results confirm that cell-type specificity is reflected by the distal enhancer repertoire and hints at a particularly distinctive SAN profile.

Chromatin accessibility contains dense information regarding cell-type differentiation, lineage specification, and maturity (6, 29–31, 33). To broadly characterize CCS ATAC-Seq peaks, we used specific criteria (see Methods) to categorize genomic loci as open or closed either across the entire CCS or in a specific CCS component. For example, if a region was open in SAN, AVN, and CM, but closed in VCS, this element was categorized as VCS-closed. Alternatively, if a region was open in CM but closed in SAN, AVN, and VCS, it was categorized as CCS-closed. Thus, we were able to obtain a high-level view of CCS-centered *cis-*regulatory logic.

We used 135,587 ATAC-Seq regions for this analysis (Figure 2B), approximately 39% of which were shared among all samples (Supplemental Figure 7B). Considering all CCS-unique regions, including individual components (SAN, AVN, and VCS) or in aggregate (CCS), we observed nearly twice as many closed versus open regions (Figure 2B), suggesting that chromatin inaccessibility may influence transcriptional regulation of CCS function. Shared CCS-enriched regions (open and closed) comprise 12% of the total possible regions, with the overwhelming majority in the CCS Closed category. Looking more closely at individual CCS components, we observed that SAN-specific regions, comprising 23% of the total possible regions, contained nearly equal numbers of uniquely open and closed loci, indicating substantial contributions by both accessible and inaccessible chromatin in the SAN. Interestingly, AVN-specific regions, comprising 5% of the total possible regions, were dominated by open loci, whereas VCS-specific regions, comprising 7%, were dominated by closed loci. Taken together, our data imply that each CCS component deploys distinct gene-regulatory logic. Interestingly, these data also suggest that restricting chromatin accessibility influences CCS gene expression, although our data sets cannot resolve the role of active silencing versus passive closure of chromatin.

To examine these classifications in more detail, we performed Genomic Regions Enrichment of Annotations (GREAT) analysis (see Supplemental Methods) to highlight Gene Ontology (GO) terms that may illuminate general themes encoded in the CCS-ATAC data set. Consistent with shared features across the CCS (1–3), all 3 components returned terms containing action potential, transmembrane transporter activity, or cardiac conduction (Figure 2, C and E, and Supplemental Figure 8, A and B). However, we also observed component-specific GO terms, such as SAN cell action potential and atria to AVN communication (Figure 2, C and E). Notably, the GO terms associated with closed chromatin (CCS, SAN, and VCS) also revealed biologically relevant terms (Figure 2, G, I, and K, and Supplemental Figure 8C), such as sarcomere organization, cardiac muscle hypertrophy, and integrin-mediated signaling pathway, which is consistent with the notion that the CCS is specialized for conduction rather than force generation (1–3, 34, 35). Collectively, the CCS regulatory logic deduced from our ATAC-Seq data sets highlight key overarching principles of CCS- and component-specific gene regulation.

Next, we performed de novo motif analyses (see Methods) to identify candidate TF binding sites for each CCS cell type and chromatin accessibility status (Figure 2, C, E, G, I, and K, and Supplemental Figure 8, A–C). From these analyses, we note that TFs of the ETS, bHLH, homeobox, and MEF2 families are particularly well represented, which is consistent with previous studies implicating Etv1, Hand1/2, Nkx2-5, and Mef2 TFs in CCS gene expression and function (28, 36–42).

Finally, we identified several genomic loci that illustrate how chromatin accessibility status may relate to a gene’s function within the CCS (Figure 2, D, F, H, J, and L, and Supplemental Figure 8, D–F). Since connections between distal elements and specific gene promoters cannot be definitively resolved by chromatin accessibility data alone, we focused on examples of differential chromatin accessibility proximal to transcriptional start sites. The genomic loci containing *Isl1* and *Kcne1*, which function within the SAN and AVN, respectively, demonstrate chromatin accessibility patterns consistent with their CCS component-specific expression (1–3) (Figure 2, D and F). Similarly, the genomic loci harboring *Nppa* and *Nkx2–5*, which are known to be excluded from the CCS and SAN, respectively, show corresponding chromatin accessibility (1–3) (Figure 2, H and J). Taken together, our analysis provides context for CCS lineage–specific regulatory networks and yields a list of potential TFs that may influence CCS cell type–specific gene expression programs.

### Construction of CCS gene–regulatory networks from chromatin accessibility data.

**Figure 3:**
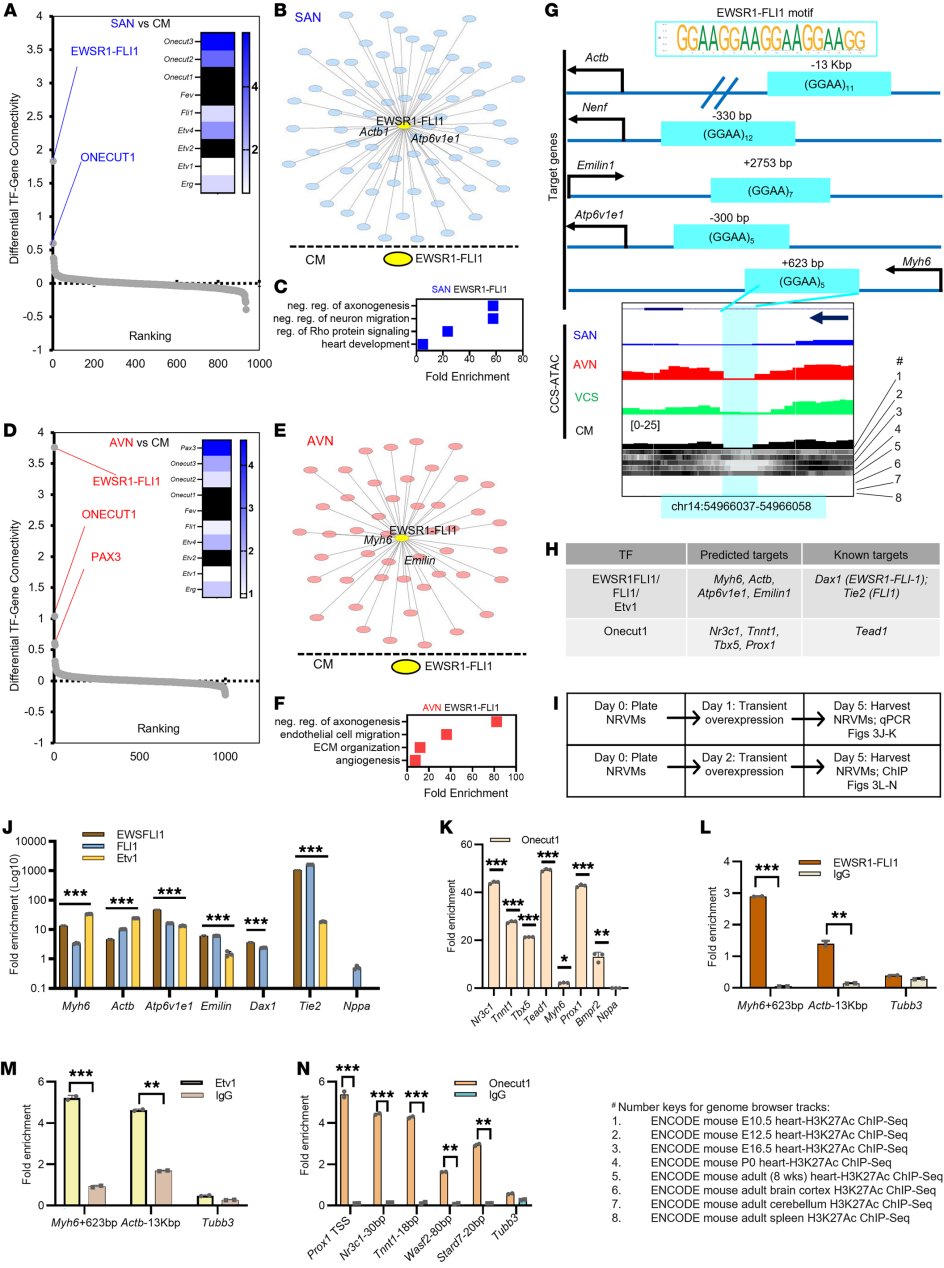
CCS-ATAC enables construction of CCS component-specific GRNs. (**A**) TF subnetworks were compared to assess SAN enrichment (blue). Inset: Heatmap shows fold enrichment in TF gene expression for SAN relative to CM. Solid black indicates undetectable. (**B**) Diagram representing a SAN-enriched EWSR1-FLI1 sub-network with selective labeling of highly connected genes. Central EWSR1-FLI1 node is shown in yellow, and individual downstream genes are depicted by light blue ovals. Proximity to the central node indicates greater connectivity. (**C**) Enriched GO terms for SAN EWSR1-FLI1 subnetwork target genes. (**D**) TF subnetworks were compared to assess enrichment in AVN (red). Inset: Heatmap shows fold enrichment in TF gene expression for AVN relative to CM. Solid black indicates undetectable expression. (**E**) Diagram of the AVN EWSR1-FLI1 subnetwork with selective labeling of highly connected genes as in (**B**). (**F**) Enriched GO terms for the AVN EWSR1-FLI1 subnetwork target genes. ECM, Extracellular matrix. (**G**) Examples of SAN and AVN EWSR1-FLI1 target gene loci with EWSR1-FLI1 consensus motifs, relative location, and number of GGAA microsatellite repeats. Genome browser view is shown for the Myh6 gene locus, with ENCODE mouse H-H3K27Ac ChIP-Seq at various time points (1, E10.5; 2, E12.5; 3, E16.5; 4, P0; 5, 8 weeks) as well as other ENCODE mouse adult tissue H3K27Ac ChIP-Seq (6, cortex; 7, cerebellum; 8, spleen. (**H**) Table of predicted and known target genes for EWSR1-FLI1, FLI1, Etv1, and Onecut1. (**I**) Experimental workflow for TF subnetwork validation in NRVMs. (**J** and **K**) Bar graphs showing target gene induction for each overexpressed TF. Error bars illustrate SE. of target gene expression among 3 independent experiments. *Nppa* served as a negative control. (**L** and **M**) Bar graphs showing genomic localization by ChIP-qPCR fold-enrichment for EWSR1-FLI1 (**L**) and Etv1 (**M**) compared with IgG control. Error bars illustrate SEM of target gene expression among 3 independent experiments. *Tubb3* served as a negative control. (**N**) Bar graphs showing genomic localization by ChIP-qPCR fold-enrichment for Onecut1 compared with IgG control. Error bars illustrate SEM target gene expression among 3 independent experiments. *Tubb3* served as a negative control. Significance determined by 2-tailed *t* test. *P < 0.05; **P < 0.01; ***P < 0.005.

Combining motif identification algorithms with chromatin accessibility data can be used to construct putative cell-type specific GRNs (43). However, these methods focus on gene proximal regulatory elements to improve the accuracy of CRE-gene assignments, such that the resulting networks will necessarily underestimate the contribution of distal CREs. Despite this caveat, we successfully confirmed established connections (1–3) and identified novel TF subnetworks within specific CCS components (Supplemental Figures 9–12). Overall, the resulting networks are structurally similar with large central hubs of broadly expressed promoter-binding TFs, including members of the SP, EGR, E2F, and TFAP gene families (Supplemental Figures 9–12). Closer examination of individual GRNs readily identifies known cardiogenic TFs (e.g., Mef2d, Nkx2-5, Isl1, Gata4, Tbx20, Hand2) (44) and several component-specific TF family members (e.g., KLF11, KLF16, and EGR3). To identify key CCS component-specific sub-networks, we quantified TF-gene connectivity and ranked TF networks by comparing each CCS component with CMs (Figure 3, A and D).

Surprisingly, we observed an extensive subnetwork for EWSR1-FLI1 in both the SAN and AVN (Figure 3, B and E). Given that EWSR1-FLI1 is a neomorphic TF resulting from a somatic-fusion event in cancer cells (45) and would not be expected to be expressed in the CCS, we were skeptical of the EWSR1-FLI1 motifs identified in the SAN and AVN (Figure 3, A and D). Instead, we reasoned that these motifs likely reflected the activity of an ETS family TFs in the CCS, which is consistent with the observed enrichment of ETS family binding motifs in SAN Open and AVN Open chromatin regions (Figure 2, C and E). Our RNA-Seq data sets demonstrated enrichment of specific ETS family members in the SAN (Figure 3A, inset) and AVN (Figure 3D, inset), and scRNA-Seq analysis indicated that *Etv1*, rather than *Fli1*, is expressed in the SAN and AVN (see next section and Supplemental Figure 19). Interestingly, we found several instances of putative target genes adjacent to accessible GGAA microsatellite repeat–containing regulatory elements (46) (Figure 3G, top). For example, the *Myh6* locus contains an extended span of chromatin inaccessibility within an otherwise accessible genomic region (Figure 3G, bottom), a known biochemical feature of TF footprints (47) that suggests the presence of a bound protein in vivo. Although FLI1 and other TFs of the ETS family typically bind to single GGAA elements, they can also bind to a subset of GGAA repeats in vitro (48), implying that ETS family TFs could bind to GGAA microsatellite sequences under specific conditions in vivo. GO analysis of predicted EWSR1-FLI1 target genes implicates processes associated with nervous system development (Figure 3, C and F) that are perhaps redeployed in SAN and AVN. We also explored predicted subnetworks for ONECUT1, the second ranked TF in SAN and AVN (Supplemental Figure 13, A–D). Taken together, our analysis defines several testable CCS regulatory networks and highlights an intriguing ETS family TF network that regulates SAN and AVN gene expression.

Given that Etv1 is required for proper CCS function (28) along with our identification of EWSR1-FLI1 motif enrichment, we hypothesized that EWSR1-FLI1 and/or Etv1 directly regulate specific target genes within SAN and AVN. To test this hypothesis, we first compiled a list of EWSR1-FLI1 and ETV1 target genes based on the GRNs constructed form our CCS-ATAC data sets (Figure 3H). Then, we used a gain-of-function assay in neonatal rat ventricular myocytes (NRVMs) to assess target gene regulation by EWSR1-FLI1, FLI1, and ETV1 (Figure 3I). Activation of *Tie2* by all 3 TFs confirmed functionality of the assay, and specificity was confirmed by *Dax1* activation by EWSR1-FLI1 and FLI1, but not ETV1 (Figure 3J). Interestingly, all of the predicted target genes tested in our gain-of-function assay demonstrated variable amounts of activation, with *Atp6v1e1* reaching induction levels as high as 32-fold by EWSR1-FLI1 (Figure 3J). Independently, we evaluated a distinct set of ETV1 targets predicted by our GRN analysis (Supplemental Table 2) and observed variable amounts of activation (Supplemental Figure 13F). We attempted to directly compare our ETV1 overexpression results with those of a previous study (49) (Supplemental Figure 13G), but technical incompatibilities between the 2 data sets precluded a straightforward head-to-head comparison. Since ONECUT1 was identified as the second highest-ranking TF in the SAN and AVN GRNs (Figure 3, A and D), we also tested its ability to activate putative target genes and similarly found varying amounts of target-gene activation (Figure 3K). Altogether, these data demonstrate the sufficiency of ETS family TFs and ONECUT1 to activate predicted downstream target genes, although they cannot distinguish between direct and indirect effects.

To directly test the ability of these TFs to bind target gene promoters, we performed a series of ChIP-qPCR experiments in the NRVM system (Figure 3I). For EWSR1-FLI1 and Etv1, we demonstrated that each bound directly to the predicted *Myh6* and *Actb* DNA genomic binding sites (Figure 3, L and M). Similarly, we observed that Onecut1 directly bound to the promoter region of 5 of the target genes that we had tested for gene expression activation (Figure 3N). From these studies, we concluded that EWSR1-FLI1, Etv1, and Onecut1 activate expression of specific target genes through direct genomic occupancy. Collectively, we provide experimental support for the proposed CCS GRNs, which can be leveraged in the future to investigate specific TF subnetworks in greater mechanistic detail.

### Validation of novel CCS enhancer elements.

**Figure 4:**
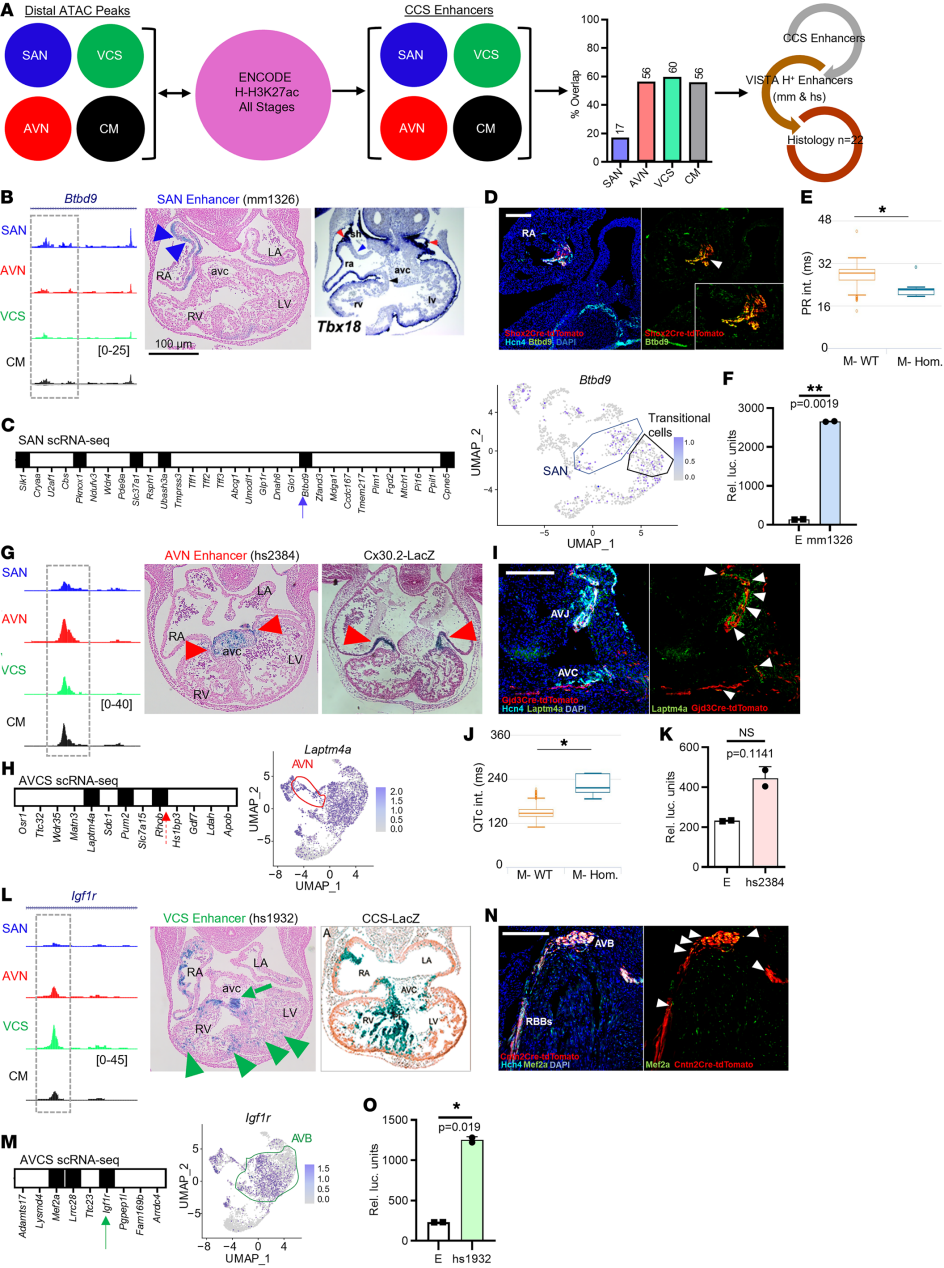
Defining CCS component-specific enhancers. (**A**) Schematic showing enhancer validation workflow. Distal chromatin accessibility regions for individual CCS- or CM-ATAC data sets were compared with ENCODE H-H3K27Ac annotations across development to identify CCS enhancers (see Methods for details). Bar graph indicates percentage overlap between H-H3K27Ac regions and individual CCS and CM data sets. Flowchart outlines how specific CCS enhancers were chosen for histological validation (see Supplemental Figure 14). (**B**) Genome browser view of SAN-candidate enhancer element mm1326, which lies within an intron of the *Btbd9* gene. Transverse section through mm1326 transgenic mouse embryo showing LacZ expression in the region of sinus horn myocardium (blue arrows). Previously published *in situ* hybridization analyses of an E10.5 embryo showing *Tbx18* expression in sinus horn myocardium (red arrows) (52). (**C**) SAN expression of genes within ±500kb of mm1326 are indicated by solid box (left). Uniform Manifold Approximation and Projection (UMAP) plot of *Btbd9* gene overlaid upon SAN scRNA-Seq atlas (right). SAN (blue) and transitional (brown) cells are indicated. (**D**) Immunofluorescence analysis showing Btbd9 expression in P0 *Shox2^Cre/+^;R26^tdTomato/+^* mouse heart cryosection. Arrow indicates Btbd9 expression in SAN region. Zoomed inset shows overlap of tdTomato and Btbd9 signals. (**E**) Box-and-whiskers plot showing decreased PR interval (using 1-way ANOVA) in Btbd9 KO mice compared with controls. (**F**) Bar graph representing RLUs for mm1326 enhancer relative to empty-luciferase construct in primary mouse SAN cells. (**G**) Genome browser view of AVN-candidate enhancer element hs2384, which lies intergenic to the *Rhob* and *Hs1bp3* genes. Transverse section through hs2384 transgenic mouse embryo showing LacZ expression in the AVC myocardium and cushion mesenchyme (red arrows). Previous report of X-Gal-stained *Cx30.2-lacZ* transgenic E11.5 embryo with expression in AVC myocardium (25) (arrows). (**H**) AVN expression of genes within ± 500 kb of hs2384 are indicated by solid box (left). UMAP plot of *Laptm4a* gene overlaid upon AVCS scRNA-Seq atlas (right). AVN cells are indicated (red). (**I**) Immunofluorescence analysis showing Laptm4a expression in P4 *Gjd3^Cre/+^;R26^tdTomato/+^* mouse heart cryosection. Arrowheads indicate expression of Laptm4a adjacent to tdTomato in transitional cells of the atrial septum and proximal AVN. (**J**) Box-and-whiskers plot demonstrating QT_c_ prolongation (using 1-way ANOVA) in Laptm4a KO mice compared with controls. (**K**) Bar graph representing RLUs for hs2384 enhancer relative to empty-luciferase construct in primary mouse AVCS cells. Data were analyzed via 2-tailed paired *t* test. Error bars illustrate SEM of luciferase expression between 2 independent experiments. (**L**) Genome browser view of VCS-candidate enhancer element hs1932, which lies within an intron of the *Igf1r* gene. Transverse section through hs1932 transgenic mouse embryo showing LacZ expression in the presumptive AVB (green arrowheads) and Purkinje fibers (dotted arrow). Expression of *CCS-LacZ* in E10.5 mouse heart (75) is shown as a reference. (**M**) AVB expression of genes within ± 500 kb of hs1932 are indicated by solid box. UMAP plot of *Mef2a* gene overlaid upon AVCS scRNA-Seq atlas. Cells comprising proximal AVB are indicated (green). (**N**) Immunofluorescence analysis showing Mef2a expression in P28 *Cntn2^Cre/+^;R26^tdTomato/+^* mouse heart cryosection. Arrowheads indicate overlap of tdTomato and Mef2a in the AVB and RBB. (**O**) Bar graph representing RLUs for hs1932 enhancer compared with empty-luciferase construct in primary mouse AVCS cells. RA, right atrium; LA, left atrium; RV, right ventricle; LV, left ventricle. *P* values were determined by 2-tailed paired t tests. *P < 0.05; **P < 0.01; ***P < 0.005. Error bars illustrate SEM of luciferase expression between 2 independent experiments.

Although an estimated 1.4 million putative enhancers have been identified in the mammalian genome (8, 9), systematic cataloging of CCS-enriched enhancer elements has not been achieved. Therefore, we sought to validate novel CCS enhancers from CCS-ATAC by taking advantage of the VISTA enhancer database (19). For this analysis, distal ATAC-Seq regions were overlapped with all ENCODE mouse H-H3K27Ac peaks across development to focus on established cardiac enhancers (Figure 4A). Each CCS component demonstrated substantial overlap except the SAN, which possesses a highly divergent CRE repertoire (Figure 2B). Importantly, many bona fide CCS enhancers are likely to be missed by this analysis, since active enhancer annotations do not yet exist for CCS components. Nevertheless, we ultimately generated a list of putative enhancers for each CCS component as well as a background set of CM enhancers from our ATAC-Seq data sets.

Next, we intersected the database of VISTA cardiac enhancers with the CM and CCS enhancer lists to create a set of VISTA enhancers for each component (Figure 4A). Interestingly, a substantial fraction (184/300; 61%) of VISTA cardiac enhancers overlapped with the elements found in our CM and CCS enhancer compendium (Supplemental Figure 14). Finally, we performed a 4-way analysis of the VISTA cardiac enhancers across CM and CCS enhancer data sets to distinguish CCS component-enriched enhancers (Supplemental Figure 14A). Among the 184 overlapping cardiac enhancers, 157 (85%) were shared with CM-ATAC, leaving 27 CCS-enriched enhancers (Supplemental Figure 14B). Fixed embryos were available for 22 of these 27 CCS enhancer elements (Supplemental Figure 14, B and C), and each of the 22 lacZ-stained embryos underwent whole mount imaging followed by analysis of serial sections (Figure 4, B, G, and L, and Supplemental Figure 15). We note 2 important limitations of our validation pipeline. First, random transgenesis can result in varying lacZ expression patterns across embryos, such that the estimated hit rate could be underestimated by analyzing only 1 embryo per enhancer. Second, extensive embryo fixation prevented marker immunostaining analysis of sections, so CCS localization of lacZ staining was assessed by comparison with published examples at similar developmental time points.

Overall, 14 of 22 VISTA enhancer elements demonstrated CCS-enriched expression, 4 elements were expressed throughout the heart, and the remaining 4 elements were not expressed in the CCS (Supplemental Figure 14, B and C, and Supplemental Figure 15). Although this yields an excellent hit rate (18 of 22; 82%), we did not formally test a random set of putative enhancer elements to quantify the precise enrichment compared with random expectation. However, we assembled a compendium of 53 active cardiac enhancers from the literature (10, 50, 51) to interpret our observed hit rate. Among these experimentally tested cardiac enhancer sequences, (14 of 53; 26%) showed expression in the presumptive CCS. Compared with this historical control (82% versus 26%; Fisher’s exact test *P* < 0.05), we observed a significant improvement in the identification of active CCS enhancers using our ATAC-Seq data sets. Moreover, we note that the control enhancer set is already enriched for cardiac regulatory elements, rather than a truly random set of elements, so the enrichment of CCS elements in CCS-ATAC compared with the control set likely represents an underestimate of the true value. Taken together, the 18 positive cases strongly support the fidelity of CCS-ATAC, which is likely to harbor many additional unidentified CCS enhancers that require further exploration.

Among the 14 elements with CCS-enriched expression, we examined 3 illustrative examples in greater detail (Figure 4, B–O). VISTA element mm1326 directed regionally restricted LacZ expression in a subset of right atrial cells and a portion of the inflow tract that contributes to the SAN, which partially overlaps the expression pattern of *Tbx18* (52) (Figure 4B). Since enhancers can regulate nearby or distal target genes, we analyzed a published Promoter Capture Hi-C (PCHi-C) data set (53) (Supplemental Figure 16) in conjunction with a SAN single-cell expression atlas (Supplemental Figure 17) to identify enriched genes within 1 Mb of the putative enhancer (Figure 4C). We identified 6 such genes (Figure 4C and Supplemental Figure 20A), including *Cpne5*, which specifically marks the mouse SAN and AVN (5). In addition, we found that another SAN-enriched candidate gene, *Btbd9*, colocalizes with SAN markers and regulates PR interval in mice (54) (Figure 4, D and E). Tissue specificity of the mm1326 element was further confirmed by transient transfection of primary mouse SAN cells, which demonstrated robust activation by the putative SAN enhancer compared to a reporter construct bearing only a minimal promoter (Figure 4F).

Within the AVN enhancer subset, VISTA element hs2384 directed LacZ expression to the developing AV canal (AVC) in a pattern partially overlapping a known AVC enhancer transgenic line, *Cx30.2-lacZ* (25) (Figure 4G). Analysis of PCHi-C and an AVCS single-cell expression atlas (4) (Supplemental Figures 18 and 20) highlighted 3 candidate target genes, *Laptm4a*, *Pum2*, and *Rhob* (Figure 4H and Supplemental Figure 20B). Interestingly, we found that Laptm4a localizes adjacent to Hcn4 and Gjd3-tdTomato in the AVC (Figure 4I), and Laptm4a-knockout mice have conduction defects (55) (Figure 4J). Although transient transfection of primary mouse AVC cells with the hs2384 element resulted in numerically increased reporter expression compared with a construct bearing a minimal promoter, the difference did not reach statistical significance in preliminary experiments (Figure 4K).

Among predicted VCS enhancers, VISTA element hs1932 directed LacZ expression to the developing AV bundle (AVB) and Purkinje fiber network (PFN), which overlap the pattern of *CCS-lacZ* expression (Figure 4L). Examination of PCHi-C and the AVCS single–cell atlas highlighted *Mef2a*, *Lrrc28*, and *Igf1r* as putative downstream targets (Figure 4M and Supplemental Figure 20C). Given that Mef2a knockout mice die suddenly with terminal cardiac arrhythmias (56), we assessed Mef2a localization within the VCS and observed expression in the AVB and right bundle branch (RBB) (Figure 4N). Furthermore, we performed transient transfection experiments in primary mouse AVC cells and found that element hs1932 demonstrated robust activation compared with a minimal promoter construct, thus confirming the specificity of this VCS enhancer element (Figure 4O). In summary, CCS-ATAC precisely identified CCS-component specific enhancers from the VISTA enhancer database, suggesting that CCS-ATAC is a rich resource for enhancer discovery and disease variant annotation.

### Annotation of cardiac rhythm variants using CCS-ATAC enhancer elements.

**Figure 5:**
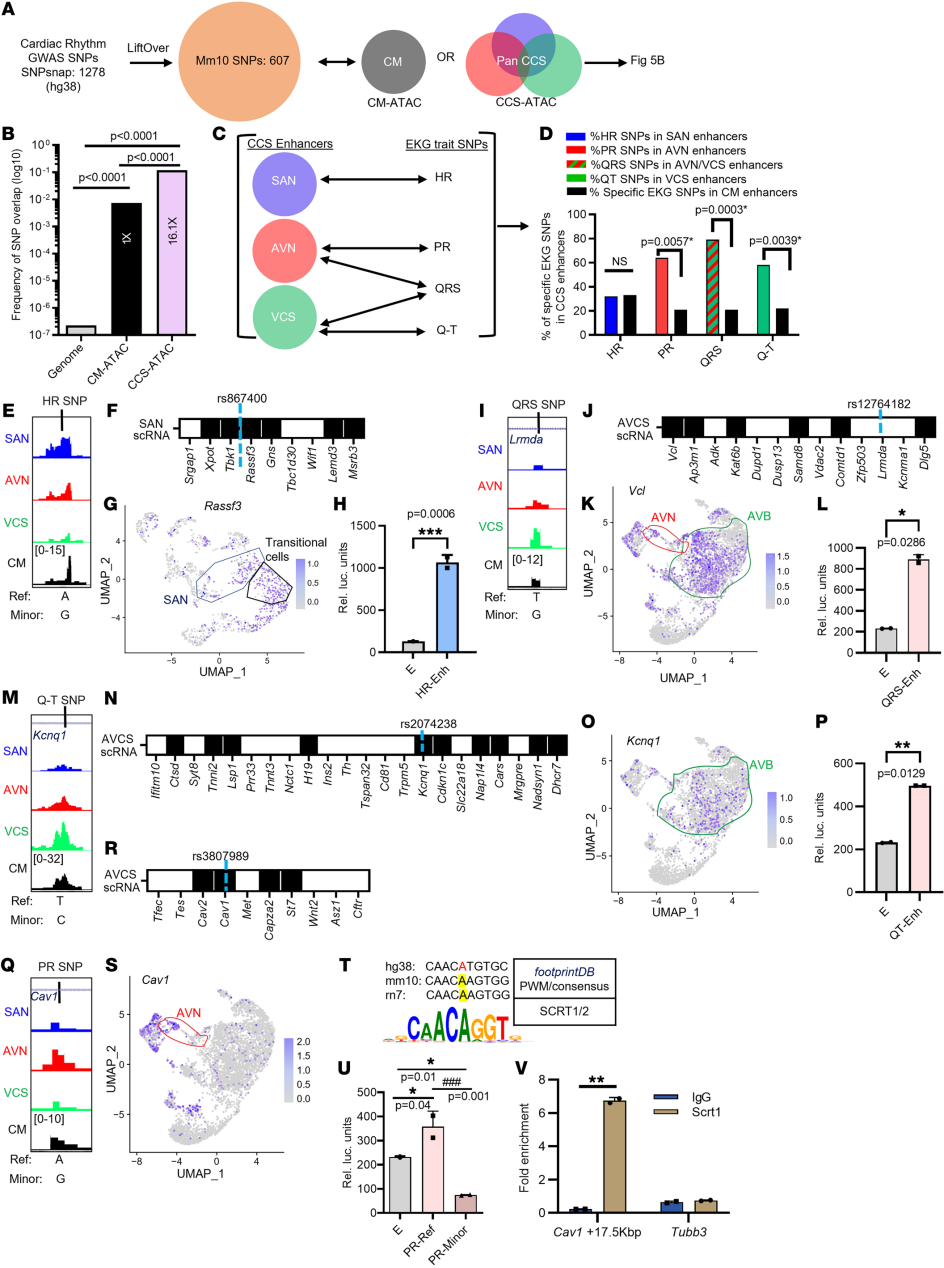
Using CCS-ATAC to improve human GWAS SNP annotation. (**A**) Pipeline for identification of syntenic cardiac rhythm-related SNPs by LiftOver to mouse reference genome (mm10) and overlap with CCS enhancers (Figure 4A). (**B**) Bar graph indicating the probability (in log_10_) of a cardiac rhythm–related SNP landing in the mouse genome (mm10), CM enhancers, and CCS enhancers. Statistical significance (*P* value < 0.001) by χ^2^ test of trends for the pairwise comparisons are shown. Fold enrichment for SNP probability relative to CM enhancers is labeled. (**C**) Workflow for comparing GWAS SNPs associated with individual EKG traits and CCS component-specific enhancers. (**D**) Bar graph demonstrating percentage of specific EKG trait SNPs landing within CCS specific enhancers. *P* values indicate statistical significance by χ^2^ test for pairwise comparisons with the CM enhancer subset. For QRS interval, AVN and VCS data sets were combined (green and red striped bar). (**E**) Genome browser view showing location of HR lead SNP rs867400 (mm10: chr10:121498835-121498836), which lies intergenic to the *Tbk1* and *Rassf3* genes, in relation to CCS-ATAC open regions. Reference and minor SNP alleles are indicated. (**F**) SAN-enriched genes within ± 500 kb of rs867400 are indicated by solid box. (**G**) UMAP plot of *Rassf3* gene overlaid upon SAN scRNA-Seq atlas. SAN (blue) and transitional (brown) cells are indicated. (**H**) Bar graph represents RLUs of enhancer containing rs867400 relative to empty luciferase in primary mouse SAN cells. Error bars illustrate SEM of luciferase expression between 2 independent experiments. Ordinary 1-way ANOVA test was used to calculate *P* values. (**I**) Genome browser view showing location of QRS lead SNP rs12764182 (mm10: chr14:22666305-22666306), which lies within an intron of the *Lrmda* gene, in relation to CCS-ATAC open regions. Reference and minor SNP alleles are indicated. (**J**) AVCS-enriched genes within ± 500 kb of rs12764182 are indicated by solid box. (**K**) UMAP plot of *Vcl* gene overlaid upon AVCS scRNA-Seq atlas. Cells comprising compact AVN (red) and AVB (green) are indicated. (**L**) Bar graph represents RLUs of enhancer containing rs12764182 relative to empty luciferase in primary mouse AVCS cells. Error bars illustrate SEM of luciferase expression between 2 independent experiments. Ordinary 1-way ANOVA was used to calculate *P* values. (**M**) Genome browser view showing location of Q-T lead SNP rs2074238 (mm10: chr7:143122498-143122499), which lies within an intron of the *Kcnq1* gene, in relation to CCS-ATAC open regions. Reference and minor SNP alleles are indicated. (**N**) AVB-enriched genes within ± 500 kb of rs2074238 are indicated by solid box. (**O**) UMAP plot of *Kcnq1* gene overlaid upon AVCS scRNA-Seq atlas. AVB cells (green) are indicated. (**P**) Bar graph represents RLUs of enhancer containing rs2074238 relative to empty luciferase in primary mouse AVCS cells. Error bars illustrate SEM of luciferase expression between 2 independent experiments. Ordinary 1-way ANOVA test was used to calculate *P* values. (**Q**) Genome browser view showing location of PR lead SNP rs3807989 (mm10: chr6:17325447-17325448), which lies within an intron of the *Cav1* gene, in relation to CCS-ATAC open regions. Reference and minor SNP alleles are indicated. (**R**) AVN-enriched genes within ± 500 kb of rs3807989 are indicated by solid box. (**S**) UMAP plot of *Cav1* gene overlaid upon AVCS scRNA-Seq atlas. AVN cells (red) are indicated. (**T**) Sequence and evolutionary conservation surrounding rs3807989 is shown with SNP location highlighted in red. Matching SCRT1/2 consensus binding site logo is shown beneath for comparison. (**U**) Bar graph represents RLUs of reference and minor allelic variant for PR SNP relative to empty luciferase in primary mouse AVCS cells. Error bars illustrate SEM of luciferase expression between 2 independent experiments. Ordinary 1-way ANOVA test was used to calculate *P* values. (**V**) Bar graphs showing genomic localization by ChIP-qPCR fold-enrichment for Scrt1 compared with IgG control at the *Cav1* locus. Error bars illustrate SEM of target gene expression among 3 independent experiments. *Tubb3* served as a negative control. *P < 0.05; **P < 0.01; ***P < 0.005. For each SNP, lifted over mm10 coordinates are used to generate the ATAC tracks. Blue dotted lines below coordinates indicate SNP location. HR, heart rate; n.s., not significant; PWM, position weight matrix.

Given the overwhelming enrichment of GWAS SNPs within distal CREs, accurate SNP annotation critically depends upon cell type specific CRE data sets (31, 57). To date, several GWAS have been conducted for EKG- and arrhythmia-related traits to identify over 1,200 candidate SNPs (1, 58–60). While previous studies have highlighted the functional relevance of individual SNPs (17, 61–64), comprehensive SNP annotation remains limited by insufficient knowledge of the CCS regulatory landscape. We reasoned that our CCS-ATAC compendium could improve annotation of human GWAS SNPs associated with cardiac rhythm, despite limited functional conservation between mouse and human enhancers (50). Supporting our hypothesis, we successfully mapped 607 of 1,278 human GWAS SNPs to the mouse genome (Figure 5A).

To quantify the impact of a CCS cell-type-specific CRE compendium for SNP annotation, we calculated the frequency with which a cardiac rhythm–related SNP lands within the CM or CCS enhancer data set (Figure 5B). As a reference point, the frequency with which 1 of the 607 SNPs landed within the genome (approximately 2.7 billion DNA basepairs) was 2.2 × 10^–7^. As expected, the CM enhancer subset is substantially enriched (approximately 19,000-fold) for cardiac rhythm-related SNPs compared with the whole genome (Figure 5B). Importantly, compared with CM enhancers, CCS enhancers demonstrated significant additional (approximately 16-fold) enrichment for cardiac rhythm GWAS SNPs. Altogether, this analysis demonstrates that our newly defined CCS enhancers substantially enrich for cardiac rhythm–related GWAS SNPs.

The list of 607 conserved SNPs represents a heterogeneous group of cardiac electrical traits and diseases, many of which are not easily attributable to a specific CCS component. To understand how component-specific enhancer data sets inform biologically relevant phenotypes, we focused on EKG traits that functionally correlate with a particular CCS component. Thus, we analyzed GWAS SNP subsets for heart rate (HR), PR interval, QRS interval, and QT interval (Figure 5C). Each trait was compared with the corresponding single CCS component-specific data set except for QRS interval. Since pathology anywhere from the AVN to the proximal bundle branches can prolong the QRS interval (65), we combined the AVN and VCS data sets to analyze the corresponding GWAS SNPs. Compared with the CM data set, each component-specific data set was substantially enriched for functionally related EKG traits, except for the SAN and HR (Figure 5D), which we attribute to the highly divergent SAN-CRE repertoire (Figure 2B) that does not overlap with the ENCODE H-H3K27ac data sets (Figure 4A). Taken together, these results clearly show that CCS component-specific enhancers augment discrimination of functionally correlated EKG traits.

Any SNP in linkage disequilibrium with the sentinel variant could theoretically be causative (66), but we fortuitously identified several sentinel SNPs that landed within CCS-ATAC peaks (see below), so we chose to examine 1 example among this subset for each EKG trait in greater detail. Although the SAN data set did not improve discriminatory power overall (Figure 5D), we highlighted HR SNP rs867400, which landed within a broad SAN-enriched accessibility peak (Figure 5E). Using PCHi-C (Supplemental Figure 22) and our SAN single-cell atlas, we identified 6 potential target genes (*Xpot1*, *Tbk1*, *Rassf3*, *Gns*, *Lemd3*, and *Msrb3*) (Figure 5, F and G, and Supplemental Figure 21A). Among potential candidate genes, *Rassf3* demonstrated the most enriched expression pattern (Figure 5G). Although it is unclear how Rassf3 could influence heart rate, Rassf-family proteins have been implicated in cardiac growth and crosstalk with the Hippo signaling pathway (67, 68). Transfection of primary mouse cells demonstrated that the enhancer containing rs867400 was active in the SAN (Figure 5H). Interestingly, this enhancer was SAN specific, as it was unable to activate luciferase expression in primary AVC cells (Supplemental Figure 21A), although, mouse transgenic analysis will be required to conclusively prove SAN specificity in vivo. Collectively, these results support the idea that SNP rs867400 influences HR via a SAN-enriched enhancer element, perhaps by regulating *Rassf3*.

The QRS interval SNP rs12764182 lies within an intronic region of the *Lrmda* locus that is preferentially accessible in the VCS (Figure 5I). Analysis of PCHi-C and our AVCS single-cell atlas identified 6 potential target genes (Figure 5, J and K, and Supplemental Figure 21B), including *Vcl*, which is required for normal AV conduction in mice (69). Furthermore, transient transfection analysis revealed that the underlying enhancer element was functional in primary mouse AVC cells (Figure 5L). Taken together, these data demonstrate that SNP rs12764182 resides within a functional AVN enhancer to potentially regulate *Vcl*.

The QT interval SNP rs2074238 overlies a site of preferential VCS accessibility (Figure 5M) and is associated with 10 AVB-enriched genes (Figure 5, N and O, and Supplemental Figure 21C). Among these candidates, *Kcnq1* stands out for its prior association with long QT syndrome (70). Transient transfection analysis demonstrated that the candidate VCS enhancer functioned robustly in primary AVC cells (Figure 5P). Altogether, these results suggest that rs2074238 resides within an enhancer that is active in the VCS and identifies several potential candidate genes, including *Kcnq1*.

For the PR interval SNP rs3807989, we found a corresponding region of AVN chromatin accessibility (Figure 5Q) and AVN-enriched expression of *Cav2*, *Cav1*, *Capza2*, and *St7* (Figure 5, R and S, and Supplemental Figure 21D). Notably, cardiac Cav1 knockout mice display reduced conduction velocities (71), and the PR interval SNP overlies a strong consensus SCRT1/2 binding site in which the minor allele is expected to alter DNA binding (Figure 5T). Since the genomic sequence surrounding rs3807989 is well conserved between rodents and humans (Figure 5T), we tested whether the variant allele reduced enhancer activity by performing transient transfection analysis (Figure 5U). As expected, the enhancer containing the reference allele was active in mouse primary AVC cells. Interestingly, we not only observed that the enhancer containing the minor allele had diminished transcriptional activity, it was significantly lower than empty vector, suggesting active repression. To evaluate whether Scrt1 could directly bind to the SNP-containing regulatory element, we performed ChIP-qPCR following overexpression of Scrt1 in NRVMs (Figure 5V). This experiment confirmed that Scrt1 did bind to the presumptive *Cav1* enhancer sequence. Based on this observation, we speculated that the minor allele not only diminished SCRT1/2 binding but may have created or enhanced binding of an active repressor, although additional studies are required to confirm this hypothesis. Thus, for the PR interval SNP rs3807989, we provide compelling evidence that the underlying enhancer is functional in the AVN; the variant allele functions by a loss-of-function mechanism; and Scrt1 binds directly to this AVN enhancer sequence. Collectively, these examples demonstrate the utility of CCS-ATAC for annotating EKG GWAS SNPs and generating viable hypotheses for experimental validation.

## Discussion

Here, we describe what we believe to be the most comprehensive CCS CRE compendium to date. Analysis of global patterns of chromatin accessibility in these data sets suggested that individual CCS components are likely to implement unique regulatory strategies to achieve distinct functionality. We also leveraged motif-searching algorithms in conjunction with chromatin-accessibility data to generate functionally relevant GRNs for each CCS component. Importantly, we successfully validated several GRN predictions, and we identified ETS and Onecut TFs as potential regulators of CCS gene expression. Underscoring the functional importance of our CCS-ATAC data set, we validated several CCS-enriched enhancer elements from the VISTA database and demonstrated activity for a subset of enhancers in primary cells. Finally, we showed the utility of CCS-ATAC for improved annotation of GWAS SNPs associated with cardiac rhythm. Collectively, these results illuminate several key aspects of CCS component function and provide a rich database for future mechanistic investigation.

Prior studies have begun to elucidate the molecular underpinnings of SAN, AVN, and VCS formation (1–3). In the current study, the creation of CCS component-specific regulomes allowed us to compare broad regulatory themes across the entire CCS, individual components, and working CMs (Figure 2B). Strikingly, we observed several unique cis regulatory strategies within the CCS. First, closed loci dominated the CCS chromatin landscape, suggesting that repressive transactions may play an important role in distinguishing the transcriptional programs of working and conducting myocytes. Consistent with this notion, several transcriptional repressors function during CCS specification by inhibiting expression of myocyte structural components (1–3). Second, the SAN possessed the most strikingly divergent CRE repertoire with equal numbers of uniquely open and closed loci. Perhaps this widespread regulatory reorganization stems from its proposed derivation from a distinct cardiac progenitor lineage (2). Third, the AVN appeared to rely on uniquely open elements for lineage-specific function, while closure of specific loci is the dominant theme for the VCS. Interestingly, VCS specification occurs late during embryonic development by recruitment from dividing ventricular trabeculae (1–3), thus suggesting the attractive hypothesis that VCS lineage commitment is orchestrated by repression of specific ventricular genes to distinguish VCS myocytes from working CM progenitors. Taken together, our observations are consistent with previous analyses of CCS formation and function, yet the precise mechanisms by which enhancer deployment activates gene expression in each case remains to be completely understood.

Recently, there has been a growing appreciation that most human phenotypic and disease variation resides within the noncoding genome. In this regard, epigenomic profiles of specific rare cell types, such as pancreatic β cells and individual neuronal subtypes, have been particularly informative and have greatly accelerated interpretation of genomic variants identified in large-scale GWAS. Until now, however, comprehensive CCS epigenomic data sets have not been available for accurate interpretation of the many GWAS SNPs associated with EKG traits and cardiac arrhythmias. Indeed, we showed that CCS-ATAC improves annotation of EKG- and cardiac rhythm-related GWAS SNPs (Figure 5, B–D) and identified several feasible enhancer-gene candidates for comprehensive mechanistic investigation in the future. Importantly, we noted that our ATAC-Seq profiling was performed at a single time point in adult animals and that more extensive profiling of developmental time points is likely to further improve SNP annotation (72). Moreover, we predicted that mapping active enhancer marks, such as H3K27Ac, in individual CCS components will further enhance interpretation of genomic variants, especially for the SAN, where existing cardiac H3K27Ac data sets demonstrated poor overlap (Figure 4A). Further improvements in SNP annotation will arise from profiling human CCS tissues and distinguishing between redundant and necessary enhancers by systematic perturbation. Nevertheless, CCS-ATAC provides a key first step toward detailed interpretation of clinically relevant genomic variation related to cardiac rhythm and should thus serve as a valuable resource for annotating new variants as they are discovered. We envision the current study as an initial roadmap by which to guide the necessary future investigation into the mechanistic basis of regulatory variation and its effect on CCS function.

Compared with transcriptome data, chromatin accessibility provides unique information above and beyond cell type classification (29). Based on seminal studies from the ENCODE consortium, DHS-Seq data has been used to uncover DNA footprints of many TFs, infer TF regulatory networks, localize common disease-associated variants, and decode cell fate and lineage relationships (73). However, large data sets from a massive library of cell lines, tissues, and developmental time points were required. Using CCS-INTACT, we successfully generated CCS subtype–specific regulatory landscapes, which comprise the CCS-ATAC database, and that enabled systems-level analysis of the CCS and a more nuanced appreciation of individual components. Future mechanistic analysis of the many candidates highlighted in the current study promises to broaden our understanding of the developmental transitions that orchestrate CCS lineage-specific function and, subsequently, malfunction, to cause cardiac dysrhythmias.

## Methods

See Supplemental Methods for extended methods and materials.

### Mouse strains.

The *R26R^tdTomato/tdTomato^* reporter (strain 007914), *C57BL/6J* (in the study referred to as WT; stock number 000664), and B6;129-*Gt(ROSA)26Sor^tm5(CAG–Sun1/sfGFP)Nat^*/J (6) (referred to as Rosa26-SunTag; stock number 021039) mice were obtained from the Jackson Laboratory. We used previously characterized KI-Cre driver lines that label the SAN (23) (*Shox2^KI–Cre^*), AVN (4) (*Gjd3^KI–Cre^*), and VCS (24) (*Cntn2^KI–Cre^*).

### Lineage labeling strategy to obtain CCS-enriched nuclei.

To isolate cell-type-specific nuclei, we used Rosa26-Sun-Tag (6) mice in combination with specific CCS Cre driver lines and the R26-tdTomato reporter allele. Whole P28 hearts were harvested and placed in cold 1X PBS to remove excess blood. tdTomato fluorescence was used to distinguish Cre^+^ hearts from Cre^–^ hearts. SAN, AVN, and ventricular endocardium were microdissected from Cre^+^ hearts guided by tdTomato expression using an epifluorescent microscope (Zeiss Stemi SV11 dissection microscope equipped with epifluorescent and bright field illuminators) to maximize enrichment of labeled CCS tissue. Microdissected tissue pieces were pooled from multiple Cre^+^ hearts for each CCS component. The number of Cre^+^ animals required to obtain enough nuclei for performing ATAC-Seq and nuclear RNA-Seq (2 biological replicates per CCS component for both ATAC- and RNA-Seq) were as follows: *Shox2^KI–Cre^* (SAN) = 35, *Gjd3^KI–Cre^* (AVN) = 28, and *Cntn2^KI–Cre^* (VCS) =25. We used our previously described protocol for 2.1 M sucrose buffer/2.2 M sucrose cushion for CM nuclei–based nuclei isolation (22). The pure CM nuclei (input) obtained at the end of ultracentrifugation was immunolabeled with anti-Myc antibody (Invitrogen, PA1-981). Subsequently, the recommended protocol for anti-Rabbit IgG Microbead–mediated (Miltenyi) magnetic separation/enrichment of immunolabeled nuclei was performed with MACS MS columns (Miltenyi). Both the flow through (FT) and eluant fractions were collected and mounted with Vectashield + DAPI (Vector Labs) on glass slides to visualize sfGFP^+^ nuclei under a confocal microscope. We evaluated sensitivity, specificity, and fold enrichment of the MAN-IP assay for each CCS component, which we collectively refer to here as CCS-INTACT, from multiple independent experiments. Representative evaluation is shown in Supplemental Figure 1. CM nuclei isolated from WT P28 mouse hearts were used for comparison. We also confirmed coexpression of native sfGFP and myc in the same nuclei during CCS INTACT experiments.

### Functional validation of enriched TF subnetworks.

To evaluate the sufficiency of nodal subnetwork TFs to activate the predicted target genes, we performed overexpression in NRVMs. We reasoned that a totally heterologous cell culture system, such as HEK or COS cells, would not harbor the transcriptional milieu required to activate reporter gene expression. In contrast, we were concerned that primary cells may already express the subnetwork TF, such that overexpression would not be capable of further activating reporter gene expression above basal levels. NRVMs were harvested using established protocols and plated at the desired density on day 0. Cells were transfected on day 1 with expression plasmids for the indicated TFs. mCherry was used to detect transfection efficiency and compare fold enrichment of target gene expression upon overexpression of individual TFs. Lipofectamine 3000 reagent (Invitrogen) was used for transient overexpression of the TFs in NRVMs. After 72 hours of transfection, cells overexpressing the TFs were harvested for cellular RNA using the ZR-Duet DNA/RNA MiniPrep Plus kit (Zymo Research). RNA was converted to cDNA using SuperScript III First-Strand Synthesis System (Invitrogen). Lists of target genes for each enriched TF were bioinformatically obtained from the Cytoscape GRNs. Quantitative PCR (qPCR) was performed on target genes that were closest in distance from the TF node in the TF-GRNs — i.e., targets with highest TF-gene interaction score. qPCR for each marker gene was done in triplicates. Positive control marker genes for each TF were also included in the qPCR based on prior studies. Gene expression was normalized to 18s rRNA. Fold enrichment of target gene expression for each TF was calculated relative to mCherry overexpression in NRVMs. To compare Etv1 qPCR results with bulk RNA-Seq from a prior study (49), we downloaded the associated data set, and differentially enriched genes were assessed in parallel.

### Functional validation of CCS candidate enhancers and GWAS SNPs.

Primary SAN and AVCS cells were isolated using the Pierce Primary CM Isolation Kit (Thermo Fisher Scientific) upon gross anatomical dissection of P6 mouse hearts. Primary cells were plated on 24-well plates coated with fibronectin (Sigma-Aldrich) on day 0. Twenty-four hours after plating primary CMs, luciferase constructs were transfected using Lipofectamine 3000. For each test, we used 2 biological replicates including enhancer elements or SNP alleles of GWAS variants. After 72 hours of transfection, cells were harvested for luciferase assays. Cell lysates were prepared using 1× passive lysis buffer (Promega, E1941). 100 μL of cell lysate was incubated with an equal amount of Brightglo reagent (Promega) in a 384-well plate. Normalized luciferase units were recorded for 2 biological replicates per element using SoftMax Pro v7.0 Software on a SpectraMax M5 plate reader. SAN cells were used for functional validation of SAN candidate and HR SNP-containing enhancers. AVCS cells were used for functional validation of AVN/VCS candidate and PR/QRS/Q-T SNP-containing enhancers. Relative luciferase units (RLUs) for each test enhancer were compared with empty luciferase. RLUs of reference and minor allelic variants for the PR SNP were calculated with respect to empty luciferase.

### Immunostaining of heart cryosections.

For the enhancer candidates shown in Figure 4, all genes within 1 Mb of the enhancer element were assessed for enrichment in scRNA-Seq data sets from mouse P4 SAN and P0 AVCS. Among enriched genes, specific enhancer targets were identified based on previous evidence from the literature. Antibody staining was done for 1 target in each CCS component: SAN — Btbd9 (dilution: 1:50, Thermo Fisher Scientific, PA5-59793), AVN — Laptm4a (dilution: 1:50, Novus Biologicals, NBP1-81645), and VCS — Mef2a (dilution: 1:100, Thermo Fisher Scientific, PA5-27380) on P1 or P28 mouse heart cryosections using a previously described protocol(4).

### Data availability.

Bulk sequencing data sets generated as a part of this study are available in NCBI Gene Expression Ombnibus (GEO) under the following accession numbers: GSE152064, GSE152065, and GSE152066. The P4 SAN scRNA-Seq data set is available in NCBI Gene Expression Ombnibus (GEO) under accession number GSE153536.

### Statistics.

Statistical calculations and graphs were generated on GraphPad Prism 7 software and R studio. Statistical significance (*P* value) was calculated using Fisher’s exact test, 2-tailed paired *t* test, or ordinary 1-way ANOVA test.

### Study approval.

All animal experiments were reviewed and approved by the Institutional Animal Care and Use Committee at UT Southwestern Medical Center.

## Author contributions

SB conducted the experiments and wrote the manuscript. RKK analyzed the data and edited the manuscript. SB and NVM conceived the study and designed the experiments. SB, SG, and GOT generated the experimental data sets. SB, RKK, and SG conducted bioinformatic and statistical analyses. MZ constructed gene regulatory networks, BL generated 10× Genomics single-cell libraries; JD preprocessed scRNA sequenced libraries; CP sectioned embryos; JMS and BME provided histological expertise. SB and MB bred and maintained mice. YX and GX supervised gene regulatory network construction. GCH supervised single-cell genomics experiments. RK supervised bioinformatics analyses. NVM supervised generation of experimental data sets. RK and NVM obtained research funding for the study. RK and GCH edited the manuscript; NVM wrote the final manuscript with input from all of the authors. The order of authorship for co–first authors was determined because SB initiated the study.

## Supplementary Material

Supplemental data

Supplemental table 1

Supplemental table 2

Supplemental table 3

Supplemental table 4

## Version 1 12/01/2022

In-Press Preview

## Version 2 02/01/2023

Electronic publication
