# Supplementary material for: Global chromatin landscapes identify candidate noncoding modifiers of cardiac rhythm
Source: J Clin Invest. 2023 Feb 1;133(3):e153635. doi: 10.1172/JCI153635 (PMC9888383; doi:10.1172/JCI153635)
Supplement: Supplemental data [file jci-133-153635-s057.pdf]

SUPPLEMENTARY MATERIALS

Supplementary Methods

Antibodies

Anti-Myc antibody (rabbit polyclonal, Invitrogen, #PA1-981, dilution 2µg) were used**.** The following fluorophore-conjugated secondary antibody was used: Alexa Fluor 647 Donkey Anti-Rabbit IgG (H+L) Antibody, 1:400 (Invitrogen) to show co-localization of native sfGFP in Myc^{+} nuclei.

Confocal microscopy

Confocal images of immunostained nuclei were acquired with a Nikon A1R+ scanning confocal system. Objectives used were 10X and 20X. Excitation laser filters used were 488 nm line, green fluorescence; 555 nm line, red fluorescence; and 647 nm line, far-red fluorescence. Images were analyzed using NIS Elements Viewer v4.2 software, ImageJ, and Adobe Photoshop CS6 Extended software.

RNA extraction from purified CCS-enriched nuclei

The enriched nuclei (Mock-IP nuclei for CM and MAN-IP eluant nuclei for CCS samples) were mixed with equal volume of DNA/RNA Shield (2X concentrate provided in ZR-Duet DNA/RNA MiniPrep Plus kit, #D7003), and RNA was extracted using the same kit. Extracted nuclear RNA concentration was measured by Qubit RNA HS Assay Kit (Thermo Fisher Scientific). We extracted RNA from multiple littermates with desired genotypes until we reached >500ng of RNA (per replicate for each sample). Two biological replicates per experimental sample were used for library construction. Extracted RNA with high concentration was pooled. Next, RNA was concentrated to 10µl volume using the RNA Clean and Concentrator-5 Kit (Zymo Research, #R1015).

Chromatin Immunoprecipitation (ChIP)-qPCR sample preparation and analyses.

NRVMs were collected from post-natal day 1 (P1) rats using the Neomyt kit (Cellutron, #nc-6031) and plated at 70% density (2.5 x10^5 cells/mL) on Day 0. Cells were then transfected on Day 2 with expression plasmids: Etv1/ER81 (Mouse ETV1/ER81 cDNA ORF Clone, C-Myc tag, SinoBiological, #MG51882-CM), Onecut1 (Mouse ORF cDNA clone, Myc-DDK-tagged, NovoPro, #749369-1), Scrt1 (Lenti ORF clone of mouse Scrt1, Myc-DDK-tagged, Amsbio, #MR205267) and pLVX-FLI1 (Flag-tagged, courtesy: McFadden lab). pLVX-ZsGreen (courtesy: McFadden lab) was used to detect transfection efficiency. Lipofectamine P3000 reagent (inVitrogen, #L3000008) was used for transient overexpression of the TFs in NRVMs. For ChIP-qPCR sample preparation, NRVMs were cross-linked with 37% of formaldehyde in PBS for 15 min and neutralized by the addition of glycine to a final concentration of 0.125M for 10 min 72 hours after transient overexpression. NRVMs were then collected and washed with cold PBS. ChIP was then performed using iDeal ChIP-qPCR kit (Diagenode, #C01010180) following vendor's protocol. In brief, cell lysates were sonicated (thirty cycles of 30 s on/off) to shear DNA by using a Bioruptor Pico sonicator (Diagenode, #B01060010). Pre-washed Protein A- coated magnetic beads were then incubated for 4 hours under constant rotation with the following antibodies: c-myc (NovusBiologicals, #NB600-336), anti-flag (Sigma, #F135) and IgG (kit provided negative control antibody). Subsequently, for magnetic IP step, sheared chromatin was incubated overnight at 4 °C with antibodies described above. Finally, cross-linking was stopped, and DNA isolation was performed in IP samples as well as input. ChIP-d chromatin fragments were analyzed by qPCR using AzuraQuant™ Green Fast qPCR Mix. The rat primers used for qPCR analysis are listed in Supplemental Table 4.

Plasmid construction.

For enhancer cloning, PCR primers were designed to amplify the mm10 coordinates corresponding to 4 candidate CCS 5enhancers: mm1326, hs2384, hs1932, and hs1751. For enhancers

containing GWAS SNPs, the corresponding mm10 genomic coordinates were extended by 80 basepairs in both directions from the presumptive SNP location to design PCR primers. For Figure 5, we chose to only examine minor and reference alleles for the PR SNP (rs3807989) because there was near-perfect conservation between the rodent and human genomic sequences. Given that mouse regulatory elements were lifted over to the human genome and that we tested elements in primary mouse cells, we did not want to use candidate enhancers without high sequence conservation. For rs3807989, the minor allele version was made using PCR primers containing the appropriate mutation. PCR was performed with WT mouse genomic DNA. Next, each enhancer sequence was cloned into a destination reporter vector containing a Hsp68 promoter coupled to the luciferase reporter gene (pGL3-hsp68-luciferase) using Gibson Assembly® Cloning Kit (NEB, # E5510S). PCR and/or restriction enzyme analysis were used to confirm successful enhancer insertion.

VISTA embryo imaging

VISTA embryos were received as fixed specimens in 4% formaldehyde from VISTA database. Embryos were transferred to PBS for WM imaging following by paraffin embedding. Whole mount images were acquired with Zeiss Stemi SV11 dissection microscope equipped with bright field illuminators and Optronics Macrofire camera setup. Objectives used were 0.63X and 1.6X, and scale bars were drawn based on the objective and zoom factor used. For paraffin blocks, 7 µm sections were obtained and stained with Nuclear Fast Red (NFR) to check for regional localization of LacZ signal in embryo hearts. Histology sections and anatomical localization of LacZ expression in Figure 4 and Supplementary Data Figure 15 were evaluated by a trained veterinary pathologist, with additional guidance from Kaufman's Atlas of Mouse Development and historical reports of CCS component-specific immunostaining and enhancer activity. For the historical control set of active cardiac enhancers, individual elements were identified from the literature, and images were

obtained from the online version of each manuscript (including the Supplement) and/or the VISTA enhancer database. LacZ expression was analyzed for CCS localization similar to the CCS subset as described above.

Generation of single cell suspension for P4 mouse SAN region

P4 *Shox2*$^{KI\text{-}Cre}$ hearts were dissected in ice-cold 1X PBS. tdTomato fluorescence was used to distinguish Cre^{+} mouse hearts from Cre^{-} hearts. SAN region was micro-dissected from Cre^{+} hearts guided by tdTomato expression under epifluorescent microscope. Micro-dissected tissue pieces were pooled from (n = 5) Cre^{+} hearts. In order to obtain single cell suspension of micro-dissected SAN, we used the Pierce Cardiomyocyte Isolation Kit (Thermoscientific, #88281). The cell yield and viability were determined using Trypan Blue stain on a hemocytometer counting chamber. Since P28 CMs are too large to fit within the sequencing droplets of the 10X Chromium platform and our prior AVCS scRNA-seq dataset was obtained from cells rather than nuclei, we performed SAN scRNA-seq on hearts derived from P4 mice.

ATAC-Seq library preparation and sequencing

Following isolation and enrichment of CCS-enriched nuclei from micro-dissected P28 mouse hearts, we performed Omni-ATAC-Seq(76) to generate chromatin accessibility maps for individual CCS cellular components (SAN, AVN, and VCS) and whole heart (CM). The details of the adapted protocol is described in our previous study(77). Briefly, 50,000 nuclei (per replicate) were pelleted in ATAC-Resuspension Buffer containing 0.1% Tween-20 after counting on a Countess II FL (using DAPI light cube) Automated Cell Counter. Two biological replicates per experimental sample were used for library construction. 4nM of each ATAC-seq library was used for paired-end sequencing on an Illumina NextSeq 500 High Output (400 M) flow cell. Image analysis and base calling were performed with the Sanger/Illumina 1.9 pipeline.

Bulk RNA-seq library preparation and sequencing

RNA sequencing libraries were made using the TruSeq Stranded Total RNA with Ribo-Zero Human/Mouse/Rat kit (Illumina). Ribosomal depleted RNA was converted to cDNA using random priming strategy. To minimize batch variability, all RNA Next Generation Sequencing (NGS) libraries were generated on the same day after >500ng RNA was accumulated for each sample. The quality and concentration of the cDNA libraries for NGS were assessed using a 2100 Bioanalyzer with the High Sensitivity DNA Kit (Agilent) and a Qubit Fluorometer with the Qubit dsDNA HS Assay Kit (Life Technologies) respectively. NGS libraries were sequenced on a HiSeq 2500 (50 bp, Illumina) by paired-end sequencing. All RNA-seq libraries were run on the same flow cell.

Data processing pipeline for ATAC-seq

Raw FASTQ files were analyzed using FastQC v0.11.7 (http://www.bioinformatics.babraham.ac.uk/projects/fastqc). Adapters were trimmed using Cutadapt v.1.9.1. The trimmed reads were mapped to the mm10 assembly of the mouse genome (the University of California, Santa Cruz, version from igenomes) with Bowtie2 (version 2.3.2)(78). The duplicates were removed using picard-tools (v1.131) and blacklist regions were filtered out using samtools. The resulting set of reads were used to call the regions from each biological replicate sample. Since we do not have matched input data for these samples, we called the regions using genomic background and Poisson p-value over local region cutoff of 1e-04.

After calling significantly enriched ATAC-seq regions, for QC purposes we compared these regions and called regions shared between two samples if the regions from the two samples overlapped by at least 1bp. Regions were called unique if they did not overlap with ATAC-seq regions from any other sample. We also performed correlation analysis to measure the reproducibility

between the replicates for each sample group. Since biological replicates within each sample group showed high correlation (R > 0.98), we combined the sequencing files (fastq) for each biological replicate in a given sample to obtain more sequencing depth. Combined fastq files were processed again using the pipeline described above, and significantly enriched ATAC-seq regions were identified using HOMER with 8 week adult whole heart input serving as a control. To determine differential accessibility between samples, we used an occupancy-based analytical method(79). Specifically, the Fisher's exact test was used to identify ATAC-seq peaks that were found significantly more in a CCS component compared to CMs, or vice versa.

The ATAC-seq peaks were called using HOMER(80) (v.4.9) (http://homer.ucsd.edu/homer/ngs/peaks) with a q-value threshold of 0.05 and using random internal background within the sample. The ATAC-seq regions were called for individual replicates without input sample (i.e., HOMER uses internal background within the sample to call the ATAC-seq peak). To do the initial quality control and check replicates' reproducibility we performed the MergePeaks option. Since the reproducibility is very high, for the reminder of the paper we combined the sample replicates' (e.g. AVN-1 and AVN-2, VCS-1 and VCS-2, etc.,) raw data. The annotation of peaks was done using ChIPseeker(81). The genome-wide distribution of ATAC-seq regions on promoters, exons, introns, and intergenic regions in each of the samples was also done using ChIPseeker. We used HOMER to normalize ATAC-seq read count per basepair to 10 million total mapped tags. We generated Pearson correlation graphs in R and Venn plots using venerable R package for the ATAC-seq and ENCODE DHS samples. Enriched regions with their annotated center within 2 kb from any transcription start site (TSS) were considered promoters and separated from the putative enhancers for regions that happen to lie outside ±2 kb from annotated TSSs.

Bioinformatic analysis on ATAC-seq datasets

The shared regions between replicates in SAN, AVN, VCS, and CM samples were obtained as described under data processing pipeline above. Next a union list of shared regions for SAN, AVN, VCS, and CM samples' replicates were generated. Upon quantifying the ATAC-seq signal in the union regions list, the signal was transformed into Log2 scale. Finally, multidimensional scaling (MDS) was performed using cmdscale() function in R to visualize the level of similarity of individual replicates of a sample. MDS is a means of visualizing the level of similarity of individual cases of a dataset. MDS is used to translate "information about the pairwise 'distances' among a set of n samples into a configuration of n points mapped into an abstract Cartesian space.

To compare with mouse ENCODE DHS(82) datasets (https://www.encodeproject.org/), we used the above-mentioned combined peaks and called the peaks with adult mouse heart (H) ChIP input (https://www.encodeproject.org/, see Table S3 for accession ID) using HOMER. Peaks were compared between the samples using bedtools intersectBed tool(83). We separated the peaks into distal and promoter proximal peaks based on a ±2 kb cutoff relative to the annotated TSS. *De novo* motif analysis was performed using findMotifsGenome module available in HOMER package. To identify normalized ATAC counts that were significantly enriched in a specific CCS component compared to CM (or vice versa), Fisher's exact test was used to determine statistical significance.

Data processing pipeline for bulk RNA-seq

CASAVA v1.8.0 (Illumina) was used to demultiplex data and reads with CASAVA 'Y' flag (purity filtering) were discarded. After quality filtering and adaptor trimming using cutadapt_v1.1, Tophat v2.0.6(84) was used to align the reads to the mouse reference genome (mm10 assembly). Between 28 and 44 million reads were mapped for each sample. Nuclear transcriptome encompasses a high proportion of non-coding RNAs, novel transcripts, unspliced transcripts, intronic regions, low % of intergenic regions, etc.(85). Therefore, we included both exonic and intronic reads to detect gene expression changes. RNA-seq reads were counted in the annotated

genomic transcripts regions using featurecounts tool available in Subread package in R. Differential gene expression analysis was performed using DESeq2(86) and based on both gene TSS as well as expression signal at 3' end of genes. P values for all RNA-seq experiments are those reported by DESeq2 after false discovery rate (FDR) correction (FDR<5%). The major isoform of each gene was used for all analysis steps. GSEA(87) Gene Ontology (GO) Analysis software was used for GO analysis. To compare bulk RNA-seq samples with prior studies, we downloaded available RNA-seq or microarray datasets(88-90), and differentially enriched genes were evaluated in parallel.

GREAT analysis

GREAT analysis(91) was used for generating GO terms of neighboring genes for peaks that were common to both replicates of each ATAC-seq sample. A gene association rule was defined such that each gene is assigned a basal regulatory domain of a minimum distance upstream and downstream of the TSS (regardless of other nearby genes) Proximal: 5 kb upstream, 1 kb downstream, plus Distal: up to 1000 kb. The gene regulatory domain was extended in both directions to the nearest gene's basal domain but no more than the maximum extension in one direction. We initially attempted to use multiple-testing corrected p-values in the form of FDR to identify significant GO terms, but we did not retrieve a large list of terms that met the arbitrary FDR cutoff of <0.05. Therefore, we used the GREAT analysis as a way to confirm the specificity of our ATAC-seq datasets by focusing on binomial enrichment, since this approach tends to highlight terms with strong biological significance(91).

Transcription Factor (TF)-gene interaction analysis

For Gene Regulatory Network (GRN) inference, we used a previously described method(92). Briefly, vertebrate TF motifs from the JASPAR database(93) were used as the basis for inferring

GRNs. Only TFs with high-purity (>0.7) binding sites overlapping an ATAC-seq peak were retained(92). Binding sites located in the gene body or in the 2,500-bp region upstream of its TSS were assigned to the overlapping gene(s), and intergenic binding sites were assigned to the gene whose TSS was closest to the peak. To infer GRNs, an interaction score (defined as the sum over all TF binding sites of a TF that can be assigned to a gene) between a TF and a gene was calculated as previously described(92). This score provided a unidirectional (TFs to genes) and weighted (based on the interaction score) relationship, establishing the edges of the GRN.

Large GRN visualization

We considered TF-to-gene interactions with scores > 1 and only those TFs with >1 and < 2000 gene connections. We filtered for the top 30 TF entries. Next, the top 3 TF containing "TFAP2" were further removed from each sample to avoid over-representation of abundant TFs common to all samples. Additionally, we filtered for the top 200 genes with the most connections to upstream TFs. The node sizes of TFs are determined from their strength and number of interactions with target genes. TFs are labeled in uppercase. Nodes on the periphery of each network are same sized (not weighted) as they depict target genes (labeled in sentence case) regulated by these TFs. The inferred networks were visualized using Cytoscape v3.7.2.

Differential TF-Gene Interaction Analysis

Upon normalizing for overall TF connectivity by calculating an average TF-to-gene interaction score for each TF motif, we compared each TF score for a specific CCS component with the corresponding TF score in CMs. By computing a differential TF score, we rank-ordered TF sub-networks based on their degree of motif enrichment in each component relative to CMs. TFs that showed differential score of > +0.5 (Figure 3A, D) were used to construct the representative sub-networks and visualized using "Directed Diffuse Layout" on Cytoscape v3.7.2. TF motifs identified

in our CCS-ATAC datasets and their respective enrichment in each compartment were used for calculating TF differential scoring data.

Identification and validation of TF motifs associated with predicted target genes

Using FIMO (Find Individual Motif Occurrences) (http://meme-suite.org/doc/fimo.html), we scanned for individual matches to the EWSR1-FLI1 $(GGAA)_{n}$, Etv1 (ACCGGAAGT), and Onecut1 (AAAAATCGATA) motifs. We filtered the sequence menu to only contain databases that have additional information that is specific to UCSC Mammal genome-->Mouse--> Heart tissue DHS-seq data (ENCODE) such that MEME Suite uses tissue/cell-specific information. Using the FIMO output, we scanned the chromosome coordinates harboring the desired motif/sequence for matches with our list of target genes that showed interaction with EWSR1-FLI1 or Onecut1 in the SAN and AVN subnetworks. TF binding sites located in the gene body or in the 15 kb region upstream of its TSS were assigned to the overlapping gene(s), and intergenic binding sites were assigned to the gene whose TSS was closest to the peak. To validate candidate binding sites, ChIP-qPCR was performed using primers that were designed to amplify ~200bp surrounding the TF motif for each predicted target.

Identification of candidate CCS enhancers from VISTA Enhancer Browser

Distal SAN/AVN/VCS/CM ATAC-seq regions enriched for H-H3K27ac ChIP-seq (all stages of mouse development) signal (hereafter referred to as CCS enhancers) were overlapped with mouse (160) and human (140) VISTA heart enhancers(94). hg19 (human) or mm9 (mouse) genomic coordinates of the VISTA elements were lifted over to mm10 for our analysis. 4-way Venn diagram was generated using VENNY 2.1.0 (https://bioinfogp.cnb.csic.es/ tools/venny/), which highlighted 27 CCS enhancer candidates. 22 out of 27 transgenic embryos were available from VISTA database. Annotated expression patterns, details of reproducibility of each structure, and higher-

resolution view of individual embryos were considered when obtaining the 22 embryos for further investigation.

Mouse knockout (KO) data from International Mouse Phenotyping Consortium (IMPC)

EKG phenotyping data for Btbd9 and Laptm4 KO mice were obtained from the IMPC database (https://www.mousephenotype.org/). Statistical significance was determined by performing analysis of variance (ANOVA).

GWAS analysis

Lead GWAS SNPs related to heart rhythm (EKG parameters, Atrial Fibrillation, Arrhythmia, HR variability) or EKG parameters (Heart Rate, P-R interval/segment, QRS complex/duration, Q-T interval) were obtained from the NHGRI-EBI GWAS catalog(95) in the SNP Annotation and Proxy Search (SNAP) (SNPsnap) database (https://data.broadinstitute.org/mpg/snpsnap/database_download.html). After combining lead SNP data from multiple ethnic populations with r^2>0.9 and minimum allele frequency less than 0.01, we obtained a list of 1278 non-redundant variants. Next, we used LiftOver to intersect these 1278 SNPs with CCS-ATAC to focus on 607 syntenic mm10 lead SNPs. Hg19 coordinates from the SNPsnap database were converted to hg38 coordinates for the analysis. dbSNP (http://www.ncbi.nlm.nih.gov/SNP) was used to obtain information about reference and minor alleles for detailed investigation of selected SNPs.

To calculate the frequency of these 607 SNPs landing in CCS enhancer regions, the number of SNPs landing within the CCS enhancer dataset was divided by the total genomic space (in basepairs) encompassed by the CCS enhancer dataset. For comparison, similar analysis was performed using the whole genome and the CM enhancer dataset to show progressive enrichment of the 607 cardiac rhythm- and EKG-related SNPs. To calculate the percentage of specific EKG

SNPs that land within enhancers for a specific CCS component (e.g. what % of PR interval SNPs land on AVN enhancers), we divided the number of GWAS SNPs landing within CCS component or CM enhancers (schematic in Figure 5C) by the total number of GWAS SNPs. A comparison of these two values indicated the degree to which a specific EKG trait is enriched in enhancers for a particular CCS component versus enhancers for all CMs.

FootprintDB Analysis

FootprintDB(96) (http://floresta.eead.csic.es/footprintdb/index.php) was used for predicting TFs that bind a specific DNA site or motif. The DNA sequence (~10-11 nucleotides) encompassing the SNP of interest was used as the input. The output was a list of position weighted matrix (PWM) consensus TFs predicted to bind a similar DNA motif (curated from public databases). We chose to display the logo of the TF motif that had the highest % motif similarity score.

Genome Browser Tracks

The genomic tracks were generated using UCSC Genome Browser (http://genome.ucsc.edu/). For genome browser track visualization, normalized ATAC read counts are shown on the y-axis with genome position on the x-axis.

Promoter Capture Hi-C (PCHi-C) Maps

PCHi-C interactions from hiPSCs- derived cardiomyocytes and TADs are hosted by the WashU EpiGenome Browser(97) as a public track hub. This can be accessed by going to http://epigenomegateway.wustl.edu/browser/. The public hub (‘A promoter interaction map for cardiovascular disease genetics’) can be found under the Human Hg19 browser. PCHi-C maps were generated using the following link: http://epigenomegateway.wustl.edu/legacy/?genome=hg19&publichub=Lindsey.

10X Genomics scRNA-seq library preparation from P4 SAN

10x Genomics' scRNA-seq library was constructed following the manufacturer's instructions. Briefly, single cell suspension was washed with 1X PBS (0.04% BSA) before counting. The concentration of single cell suspension was adjusted to about 500 to 1000 cells/μL and was loaded on the 10x Genomics' Chromium™ system (10x Genomics, Pleasanton, CA) with the aim of generating 6000 to 10000 transcriptomes per channel (Chromium™ Single Cell 3′ Library & Gel Bead Kit v2, catalog number 120237). Illumina NextSeq 500 was used for paired-end sequencing of the library. Average insert size was about 400 base pairs.

Data processing pipeline for P4 SAN scRNA-seq dataset

BCL files generated by Illumina NextSeq500 were demultiplexed and converted to standard FASTQ files using mkfastq function from Cell Ranger pipeline (version 2.1.0) with default parameters. Raw UMI count matrices representing gene expression values were generated using Salmon Alevin (version 1.2.1) with default parameters, except the parameter of forceCells, which was adjusted to 10000. Ambient RNA and random barcode swapping were eliminated using CellBender with default parameters, except the parameters of expected-cells, total-droplets-included and epocs, which were adjusted to 6000, 10000 and 200, respectively. All subsequent processing was performed with Seurat (version 3.1.5). Genes detected in fewer than 3 cells or with fewer than 200 UMIs in total across all cells were filtered. The total and subset UMI counts for each cell were normalized, UMI count and percent mitochondrial genes regressed out, log transformed and scaled with SCTransform. The resulting matrix was used to perform PCA using RunPCA and clustered with RunUMAP, FindNeighbors and FindClusters (dimensions 1:10 and resolution 0.6). Uniform Manifold Approximation and Projection (UMAP) plots were used as a dimension reduction technique for visualization of cell clusters. For expression summaries, transcript expression within

>15% of CCS component-specific cells categorized a gene as expressed within a given CCS component.

List of published datasets used for analysis

1) scRNA-seq dataset for mouse AVCS: P0 Gjd3-CreEGFP mouse AVCS scRNA-seq library was previously reported(98) (accession number GSE118932) and used for AVN and VCS target gene validation. We used the fastq files and followed the same data processing pipeline as described above for SAN scRNA-seq dataset. The resulting matrix was used to perform PCA using RunPCA and clustered with RunUMAP, FindNeighbors and FindClusters (dimensions 1:10 and resolution 0.6). UMAP plots were used for visualization of distinct AVCS cell clusters. Using supervised analysis, we mapped the expression of known AVN marker genes onto the AVCS CM cell atlas and assigned compact AVN cells with established chamber and CCS markers as previously described(98).

2) ENCODE datasets: The ENCODE datasets used in this study are listed in Supplemental Table 3. Bigwig files from ENCODE portal (https://www.encodeproject.org/) were downloaded, imported, and configured on UCSC Genome Browser to visualize tracks

Supplementary Figure Legends

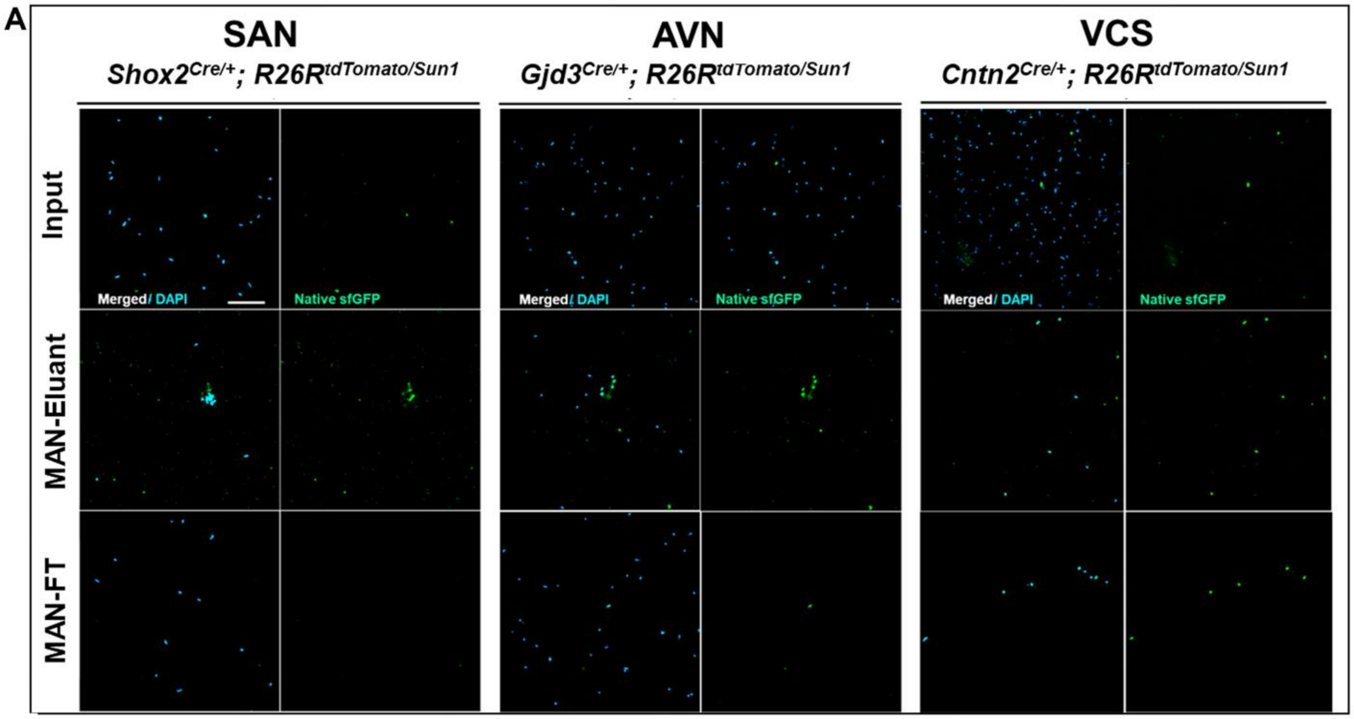

B

CCS component	Fold enrichment	Sensitivity (%)	Specificity (%) of myc IP	Avg. # of IP nuclei per P28 heart (Countess II FL)
SAN	1.8-2	96	95.4	24000
AVN	1.5-2.5	93	90	44000
VCS	1.5	70	84	65000
CM	Mock-IP	-	-	1.25 million

Supplemental Figure 1. Efficient isolation of CCS lineage-specific nuclei using CCS-INTACT
A) CCS lineage-positive nuclei were obtained from CCS$^{Cre/+}$; *R26*$^{tdTomato/+}$; *R26*$^{Sun1-2xsf-GFP-6xmyc/+}$ mice (SAN: Shox2-Cre; AVN: Gjd3-Cre; VCS: Cntn2-Cre). Confocal images of DAPI-stained nuclei showing native sfGFP expression in freshly prepared nuclei (Input), MAN-IP enriched/immunolabeled nuclei (MAN-Eluant), and MAN-IP flow-through (FT) for all three CCS lineages. Magnification 100µm.
B) Table summarizing performance of CCS INTACT MAN-IP for each CCS component. Whole hearts from wild-type mice were used to obtain pure CM nuclei. Quantification of labeled nuclei from multiple independent experiments (n=7 per Cre-driver line) for myc MAN-IP of CCS^{+} nuclei (MAN-Eluant) was used to calculate fold enrichment (ratio between sfGFP^{+} nuclei in MAN-Eluant and input), sensitivity, and specificity (in %) of myc MAN-IP assay. Average number of lineage-labeled nuclei obtained per P28 mouse heart for each reporter line is reported in the last column of the table.

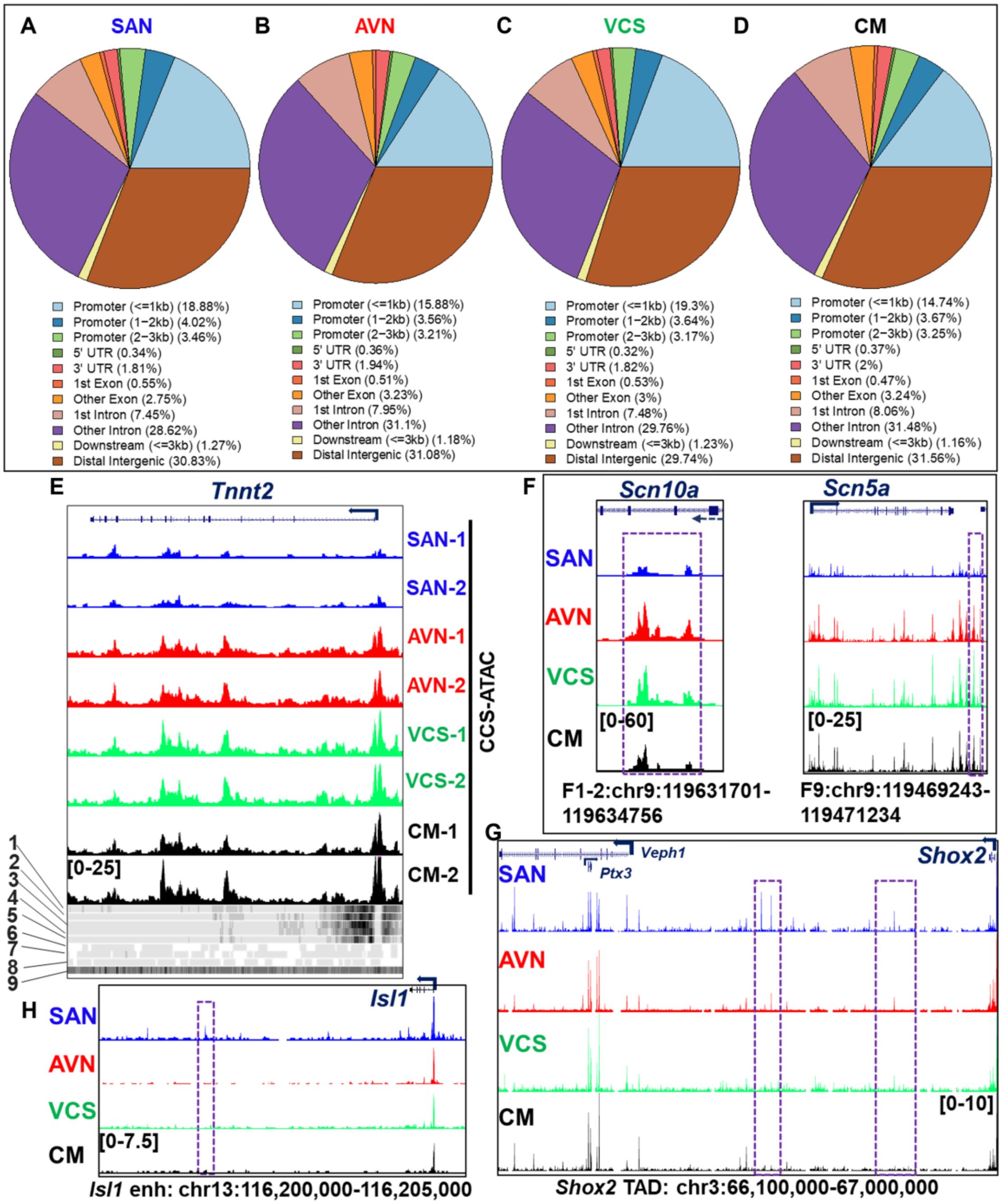

Supplemental Figure 2. Overview of CCS-ATAC-seq datasets

A) Pie chart showing genomic distribution for SAN-ATAC.

B) Pie chart showing genomic distribution for AVN-ATAC.

C) Pie chart showing genomic distribution for VCS-ATAC.

D) Pie chart showing genomic distribution for CM-ATAC.

E) Genome browser tracks for *Tnnt2* (established CM marker) locus showing each ATAC-seq sample including replicates. Number keys at the bottom of the genome tracks are as follows: ENCODE mouse H-H3K27Ac ChIP-Seq (1: E10.5, 2: E12.5, 3: E16.5, 4: P0, 5: 8 weeks); ENCODE mouse adult (8 weeks) non-cardiac tissues H3K27Ac ChIP-Seq (6: Cortex, 7: Cerebellum, 8: Spleen); 9: Placental conservation.

F) Chromatin accessibility tracks for previously established *Scn5a* and *Scn10a* enhancers(99).

G) Chromatin accessibility tracks for previously established *Shox2* TAD(100).

H) Chromatin accessibility tracks for previously established *Isl1* enhancer(100).

For each browser track, normalized ATAC read counts are shown on the y-axis with genome position on the x-axis.
Purple dotted boxes locate demonstrate enhancer fragments described in the above studies, and blue arrows indicate direction of transcription.
Mm10 coordinates for each enclosed genomic element are listed at the bottom of each browser track.
TAD, Topologically associated domain.

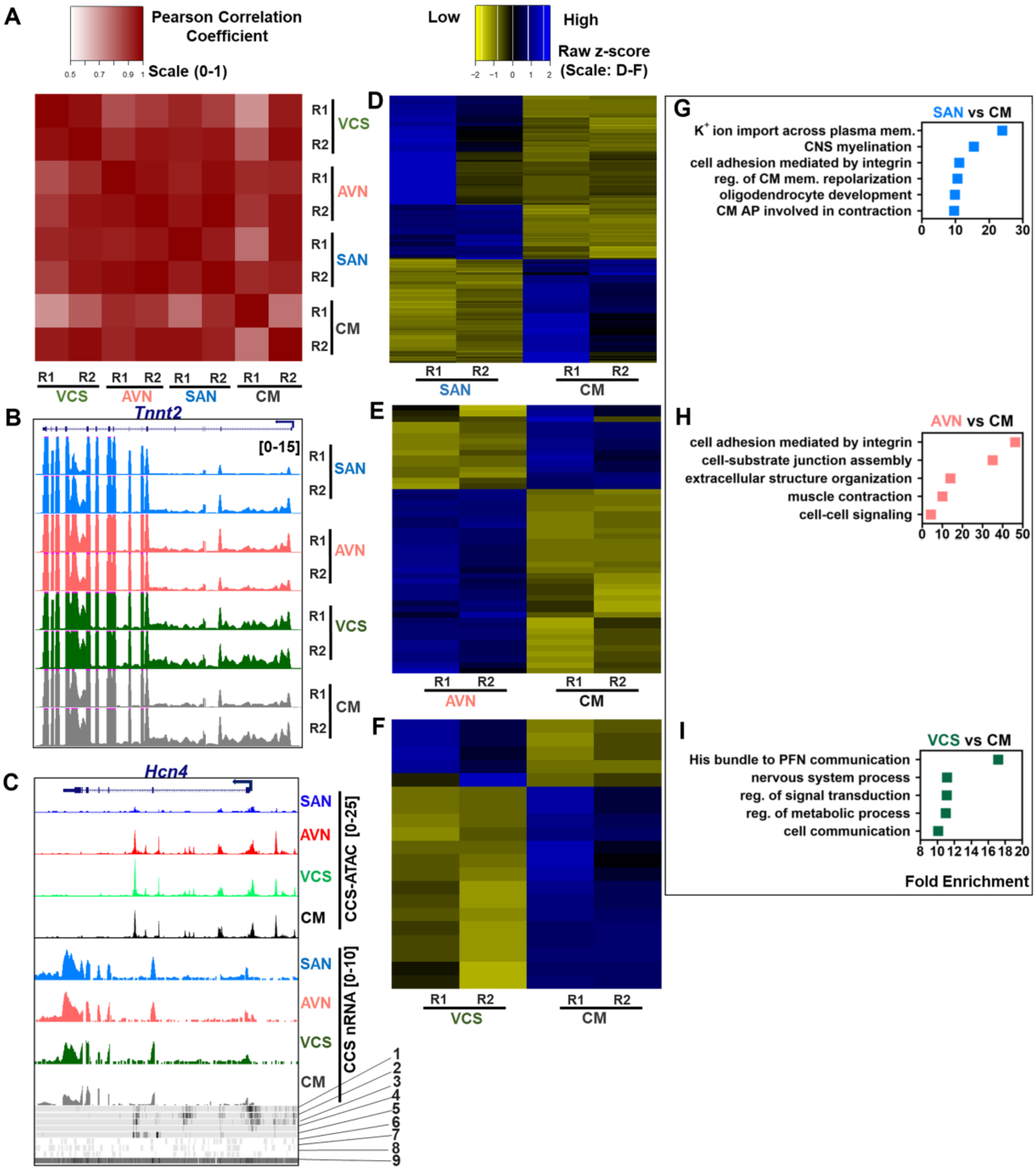

Supplemental Figure 3. Overview of CCS component-specific nuclear RNA-Seq datasets.
A) Pearson correlation matrix across CCS components and between replicates. Whole heart CM nuclei-derived RNA-seq were used as a comparison.
B) Genome browser track showing nuclear RNA expression of cardiac marker gene *Tnnt2* for all RNA-seq libraries.
C) Genome browser tracks for CCS marker *Hcn4* showing promoter accessibility (ATAC-Seq) and level of nuclear transcription (RNA-Seq). Number keys at the bottom of the genome tracks are as follows: ENCODE mouse H-H3K27Ac ChIP-Seq (1: E10.5, 2: E12.5, 3: E16.5, 4: P0, 5: 8 weeks); ENCODE mouse adult (8 weeks) non-cardiac tissues H3K27Ac ChIP-Seq (6: Cortex, 7: Cerebellum, 8: Spleen); 9: Placental conservation.
D) Heatmap representing differential gene expression between SAN and CM.
E) Heatmap representing differential gene expression between AVN and CM
F) Heatmap representing differential gene expression between VCS and CM
G) Enriched Biological Process GO terms for SAN-enriched genes.

H) Enriched Biological Process GO terms for AVN-enriched genes.
I) Enriched Biological Process GO terms for VCS-enriched genes.
For each browser track, normalized ATAC read counts are shown on the y-axis with genome position on the x-axis.
Mem., membrane; CNS, Central nervous system; reg., regulation; AP, Action potential; PFN, Purkinje fiber network.

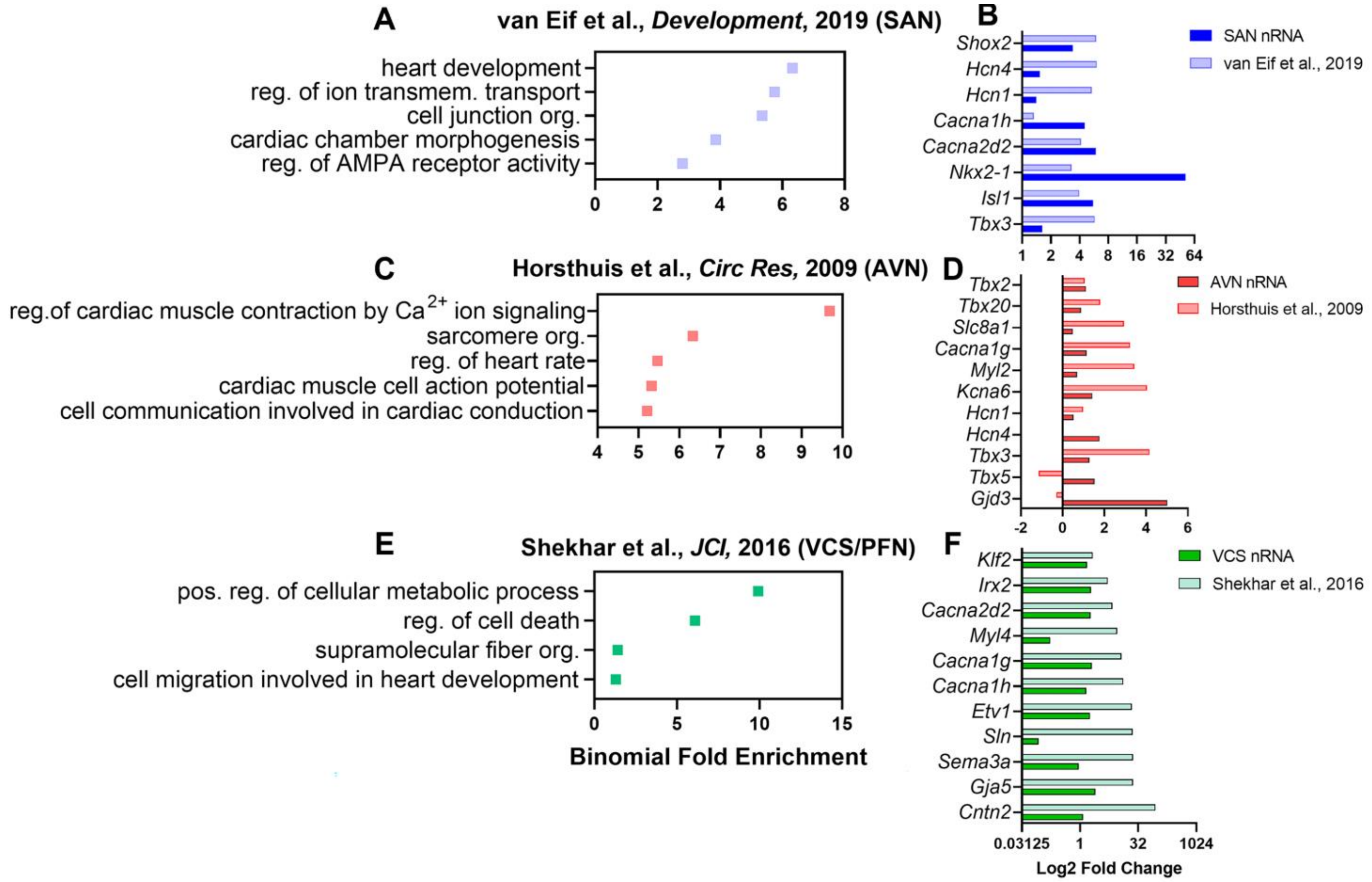

Supplemental Figure 4. Comparison of CCS nuclear RNA-Seq with published datasets.
A) Enriched Biological Process GO terms for SAN-enriched genes from Van Eif et al., 2019.
B) Comparison of selected marker gene enrichment between the current SAN nRNA-seq and published SAN datasets.
C) Enriched Biological Process GO terms for AVN-enriched genes from Horsthuis et al., 2009.
D) Comparison of selected marker gene enrichment between the current AVN nRNA-seq and published AVN datasets.
E) Enriched Biological Process GO terms for VCS-enriched genes from Shekhar et al., 2016.
F) Comparison of selected marker gene enrichment between the current VCS nRNA-seq and published VCS datasets.

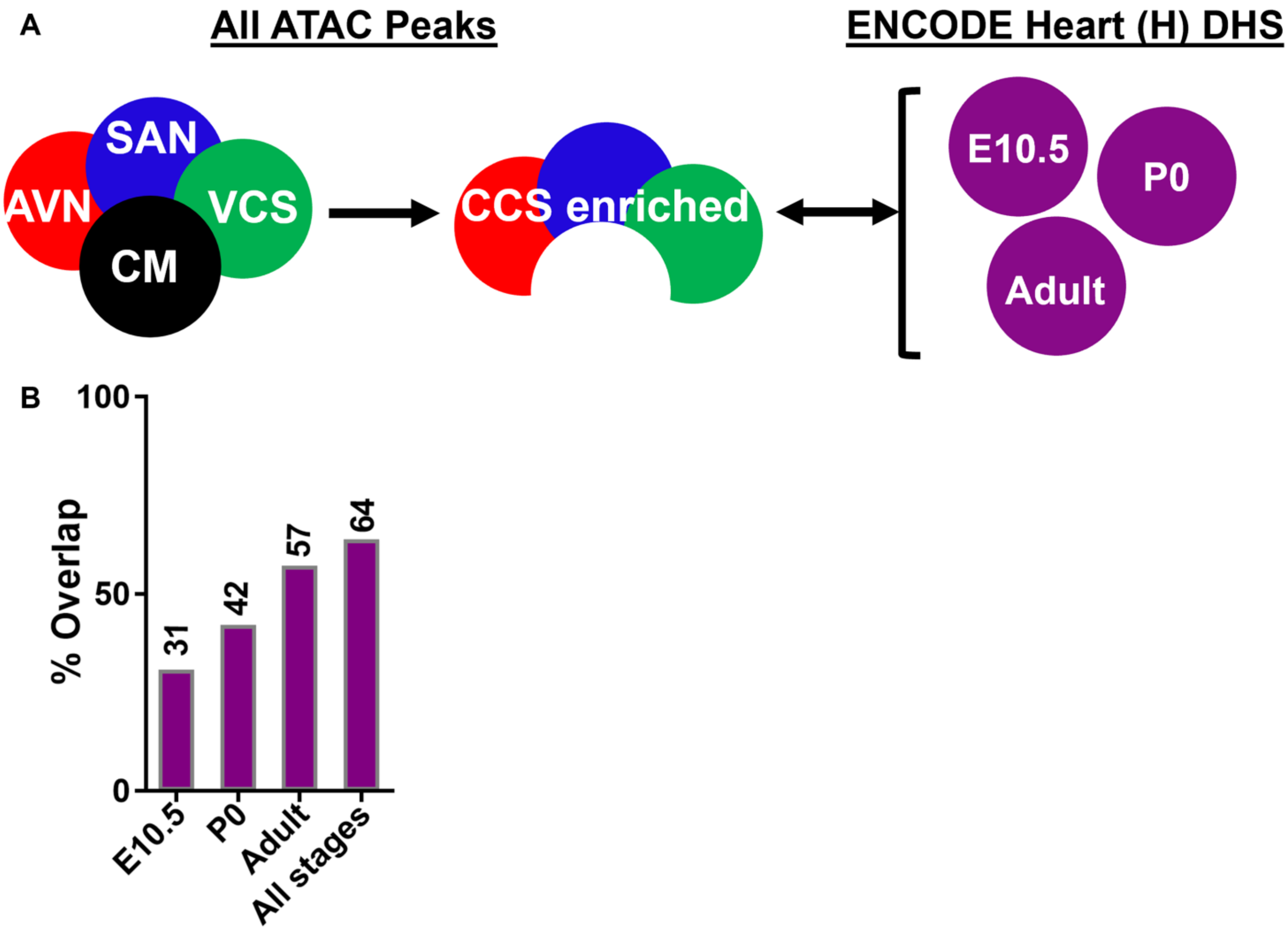

Supplemental Figure 5. Overlap between CCS-ATAC and ENCODE Heart DHS-Seq datasets. A) Outline of comparative analysis between CCS enriched ATAC-Seq (proximal and distal) and ENCODE Heart (H) DHS-Seq datasets.

B) Bar graph denoting percentage overlap between CCS enriched ATAC peaks and ENCODE H DHS-Seq derived peaks across development or in aggregate.

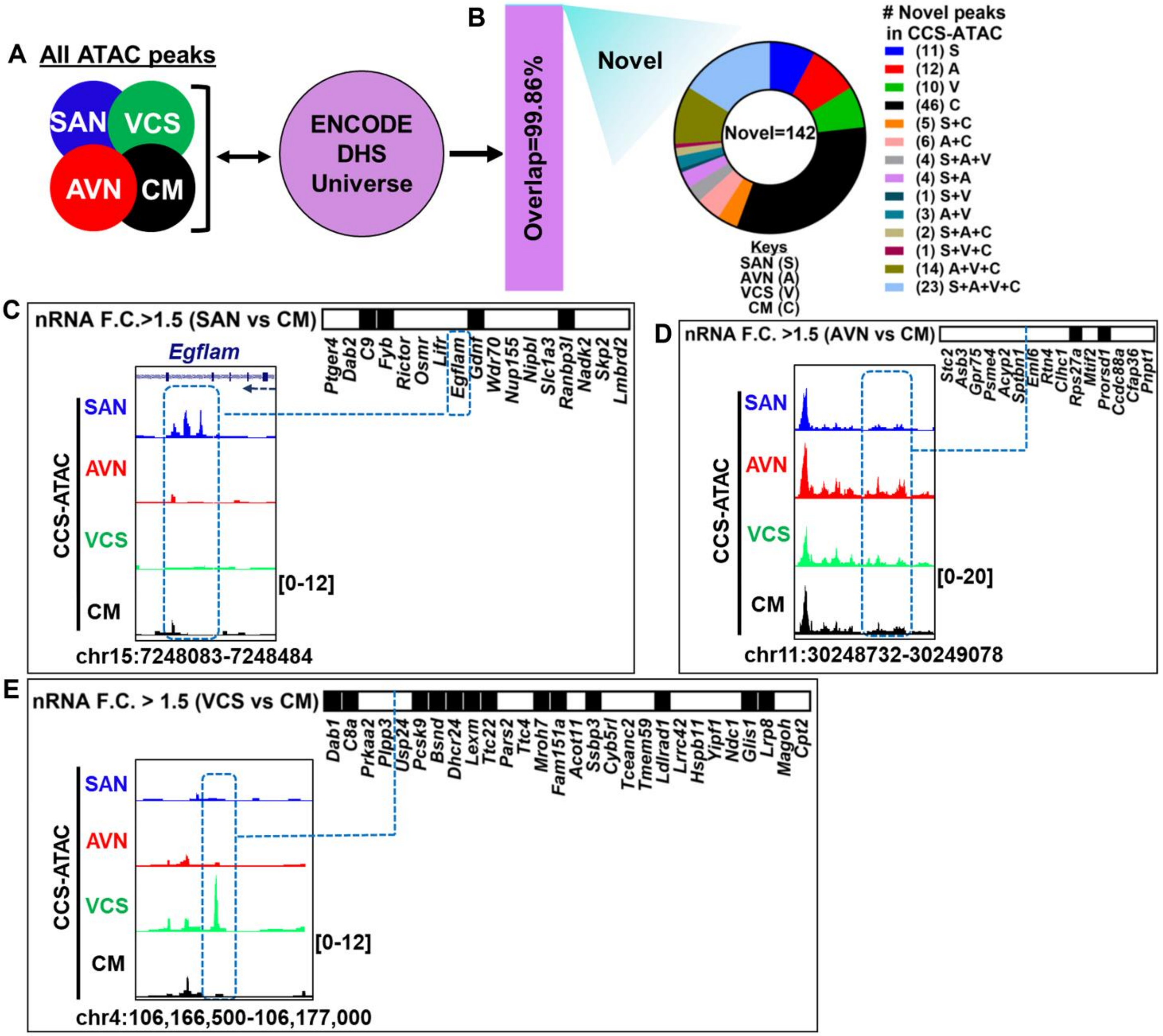

Supplemental Figure 6. Identification of novel CCS-enriched CREs.

A) Schematic of comparison between CCS/CM ATAC peaks and the non-redundant ENCODE mouse DHS universe for identification of novel CCS CREs.

B) Bar showing 99.86% overlap between ATAC and ENCODE DHS universe. CCS/CM ATAC datasets uncovers 0.14% (or n=142) novel elements (not encountered in the DHS universe). Pie chart demonstrates distribution of novel CREs for each ATAC sample or in combination with color key for each sample and corresponding number of peaks. All CCS component-specific peaks were manually inspected, and details of the novel 142 elements are provided in Supplemental Table 1.

C) Genome browser view showing CCS component-specific ATAC signal for novel SAN CRE in *Egflam* locus. SAN-enriched genes (with >1.5-fold change between SAN and CM RNA-seq datasets) within ±500kb of the novel element are depicted as solid black squares.

D) Genome browser view showing CCS component-specific ATAC signal for novel AVN CRE. AVN-enriched genes (with >1.5-fold change between AVN and CM RNA-seq datasets) within ±500kb of the novel element are depicted as solid black squares.

E) Genome browser view showing CCS component-specific ATAC signal for novel VCS CRE. VCS-enriched genes (with >1.5-fold change between VCS and CM RNA-seq datasets) within ±500kb of the novel element are depicted as solid black squares.
For each browser track, normalized ATAC read counts are shown on the y-axis with genome position on the x-axis.

Supplementary Figure 7

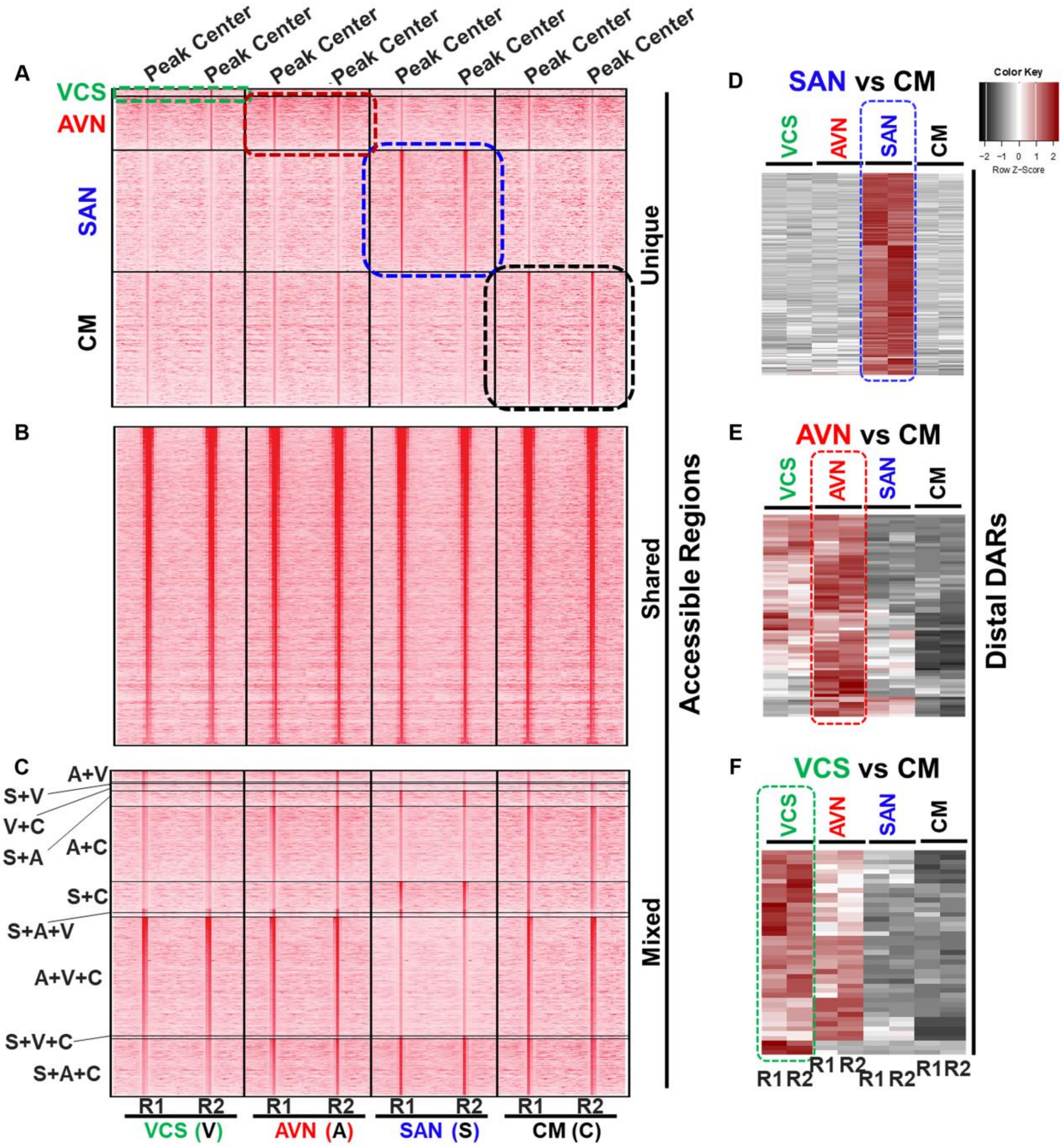

Supplemental Figure 7. Adult mouse CCS chromatin accessibility maps show distinct features.

A) Heatmap showing unique accessible regions in each CCS component (VCS: green dotted box, AVN: red dotted box, SAN: blue dotted box) and in CM (black dotted box).
B) Heatmap showing shared accessible regions.
C) Heatmap showing mixed accessible regions.
D) 4-way heatmap displaying SAN-enriched distal regions (blue).
E) 4-way heatmap displaying AVN-enriched distal regions (red).
F) 4-way heatmap displaying VCS-enriched distal regions (green).
Scale for D-F: Raw z-score
For each browser track, normalized ATAC read counts are shown on the y-axis with genome position on the x-axis.
DARs, differentially accessible regions.

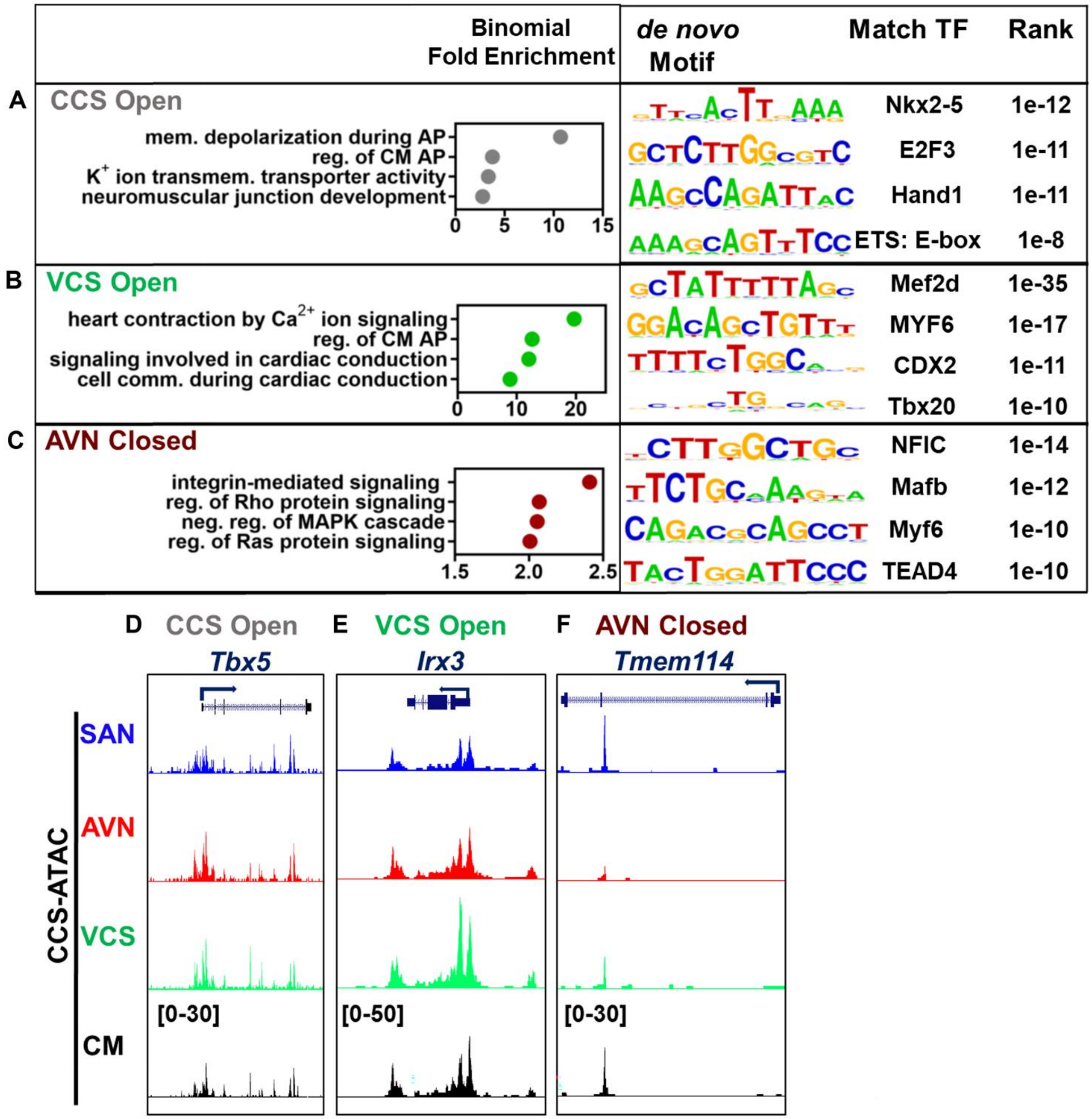

Supplemental Figure 8. Gene regulatory logic for additional CCS categories.
A) GO term identification (left) and motif discovery (right) for CCS Open regions.
B) GO term identification (left) and motif discovery (right) for VCS Open regions.
C) GO term identification (left) and motif discovery (right) for AVN Closed regions.
D) Genome browser view of the *Tbx5* locus (CCS Open).
E) Genome browser view of the *Irx3* locus (VCS Open).
F) Genome browser view of the *Tmem114* locus (AVN Closed).
For each browser track, normalized ATAC read counts are shown on the y-axis with genome position on the x-axis.
Mem, membrane; AP, action potential; reg, regulation; comm, communication; neg, negative.

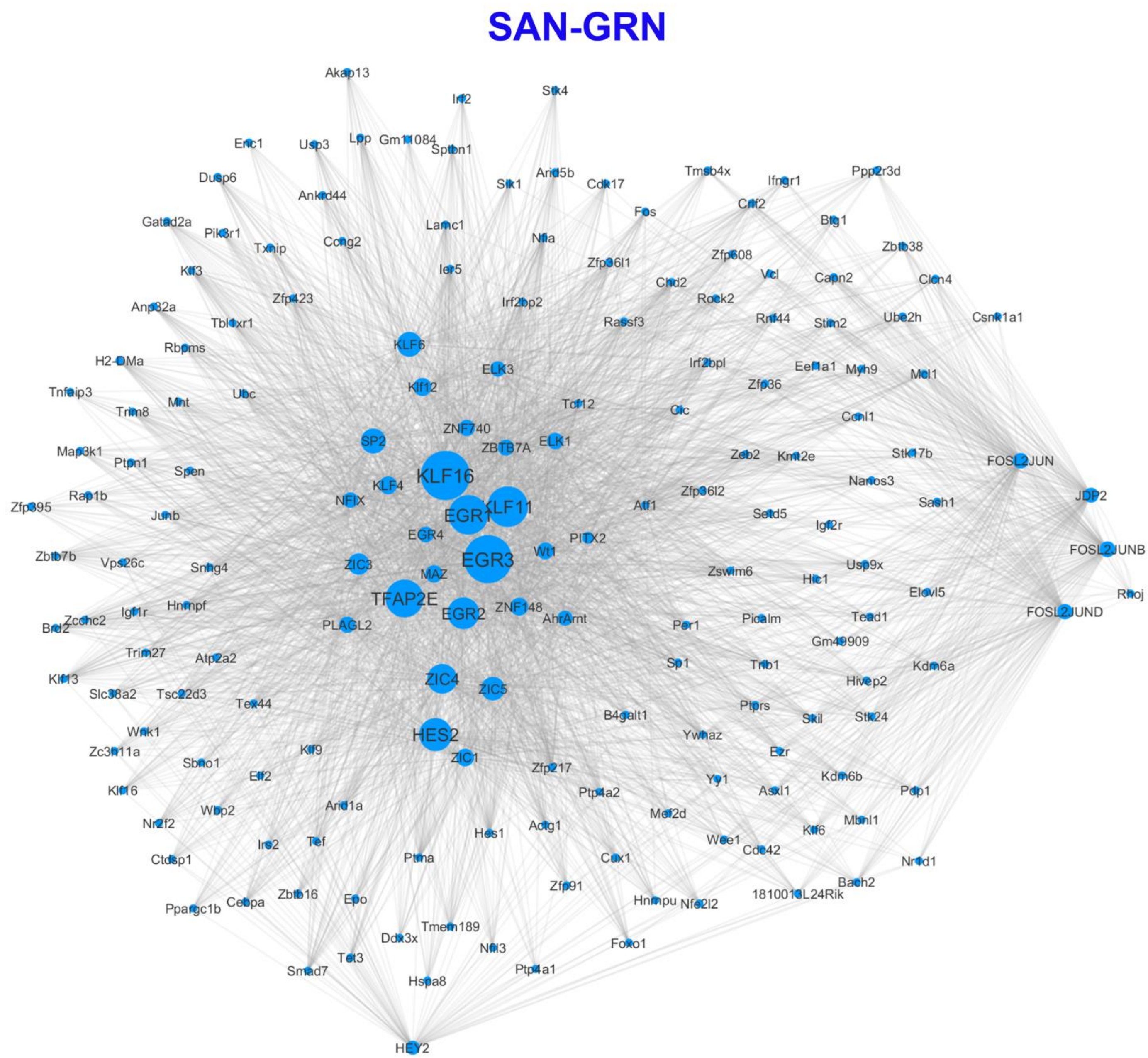

Supplemental Figure 9. Complete SAN Gene Regulatory Network (GRN).
SAN GRN representing TF nodes (gray spheres) with >1 and < 2000 gene connections. Node sizes are determined from their weight of interactions with target genes. TFs are labeled in uppercase. Spheres on the periphery depict target genes regulated by TF nodes and are labeled in lower case. Connecting lines represent interactions.

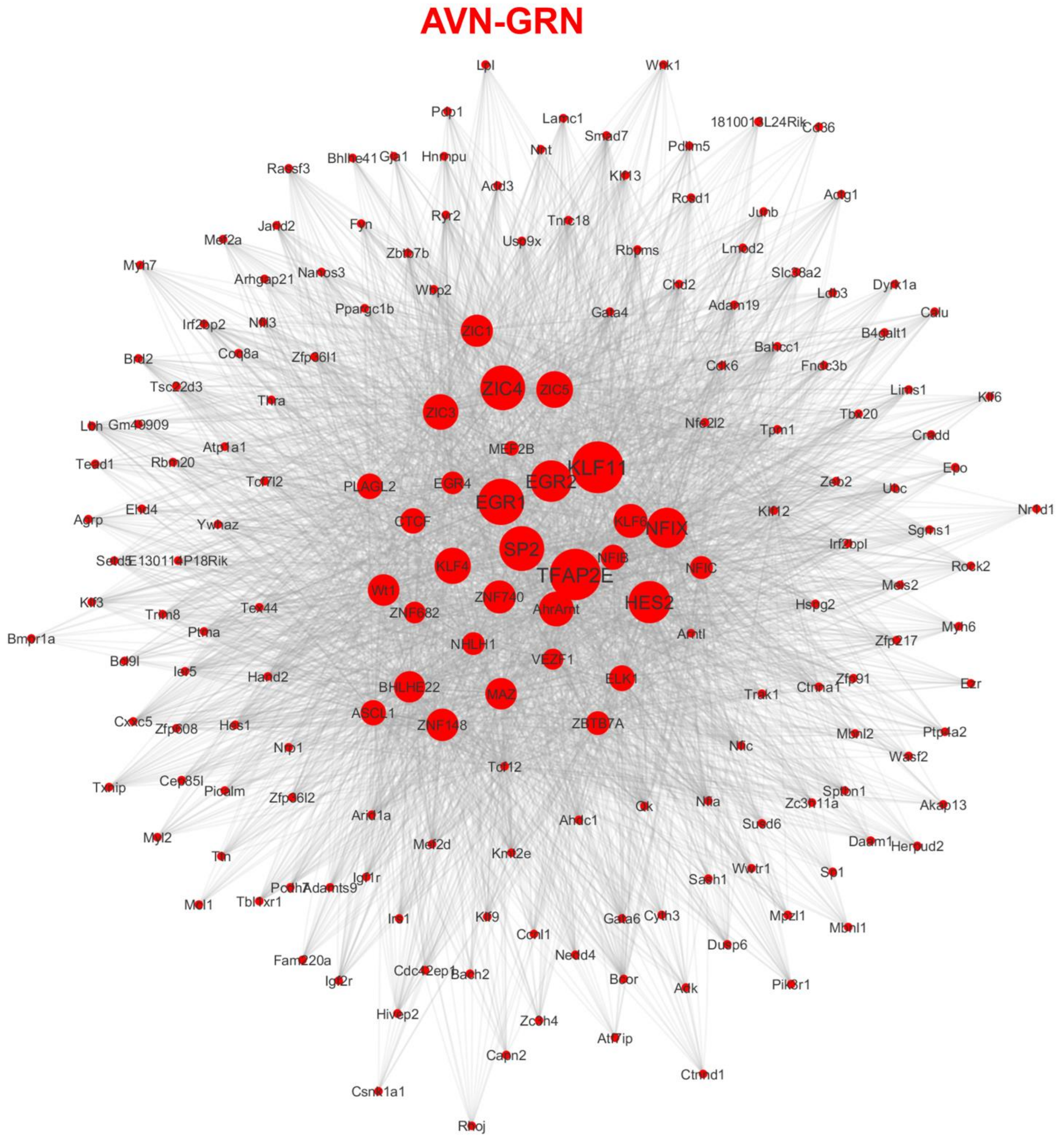

Supplemental Figure 10. Complete AVN GRN.

AVN GRN representing TF nodes (gray spheres) with >1 and < 2000 gene connections. Node sizes are determined from their weight of interactions with target genes. TFs are labeled in uppercase. Spheres on the periphery depict target genes regulated by TF nodes and are labeled in lower case. Connecting lines represent interactions.

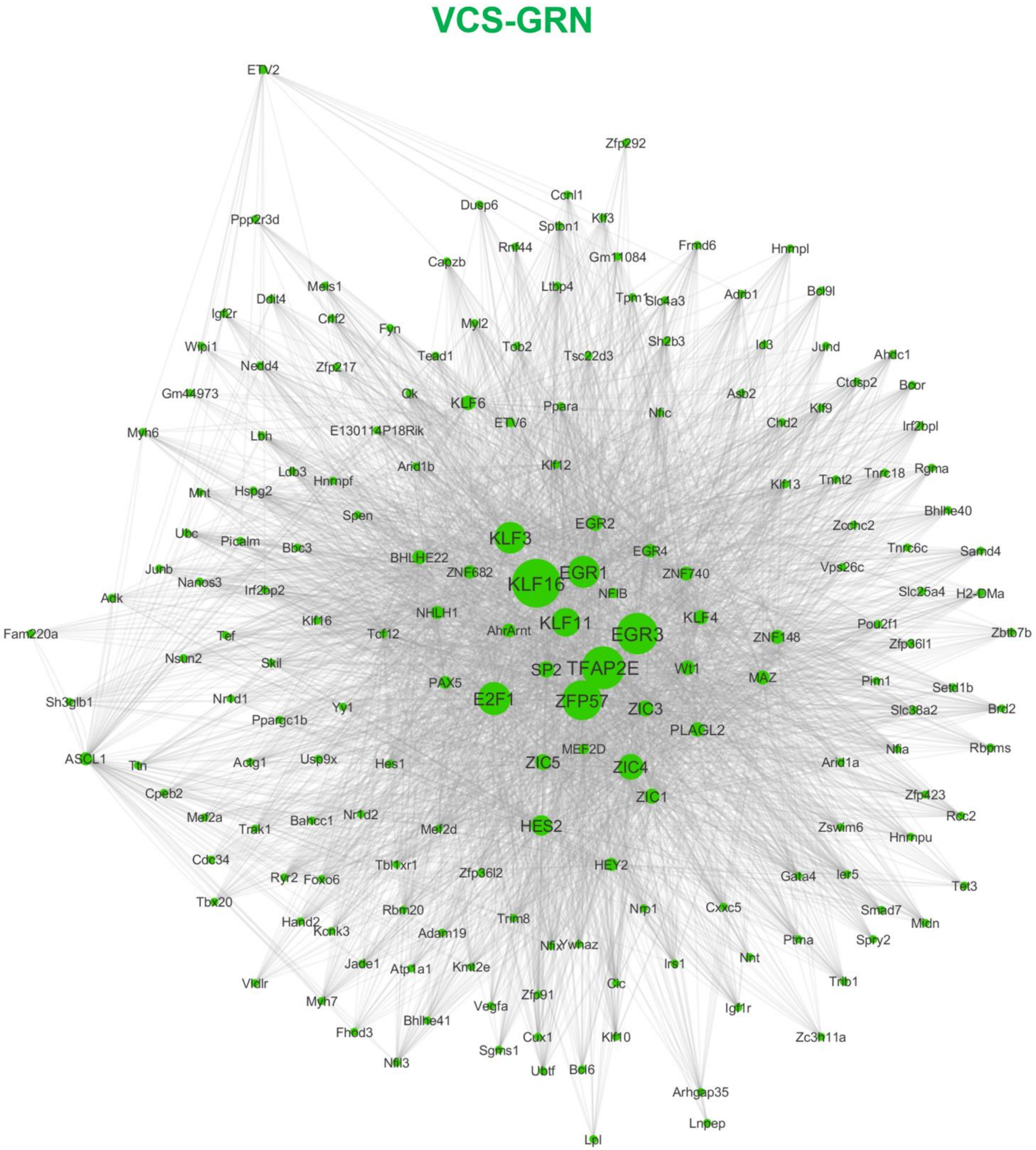

Supplemental Figure 11. Complete VCS GRN.

VCS GRN representing TF nodes (gray spheres) with >1 and < 2000 gene connections. Node sizes are determined from their weight of interactions with target genes. TFs are labeled in uppercase. Spheres on the periphery depict target genes regulated by TF nodes and are labeled in lower case. Connecting lines represent interactions.

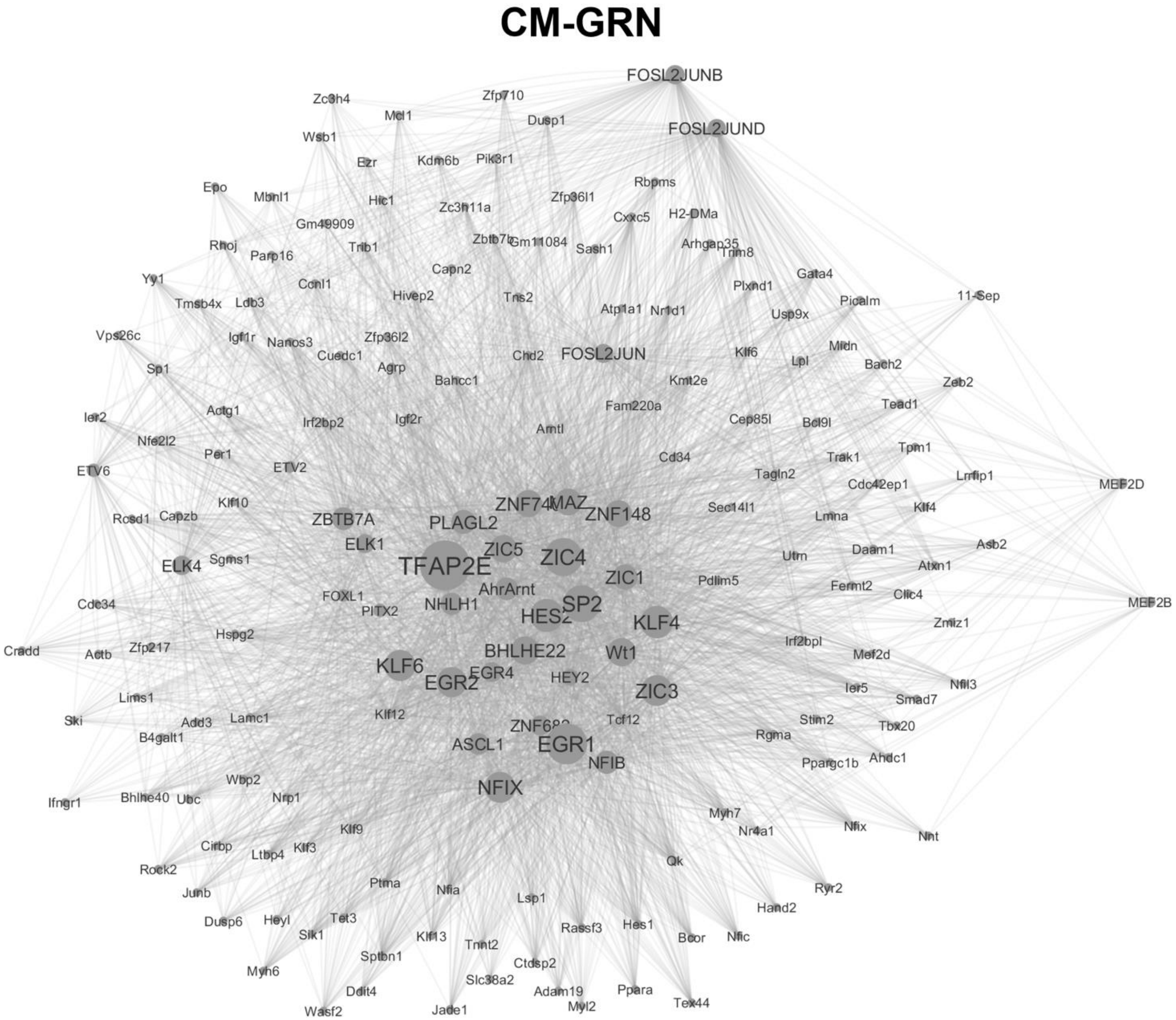

Supplemental Figure 12. Complete CM GRN.
CM GRN representing TF nodes (gray spheres) with >1 and < 2000 gene connections. Node sizes are determined from their weight of interactions with target genes. TFs are labeled in uppercase. Spheres on the periphery depict target genes regulated by TF nodes and are labeled in lower case. Connecting lines represent interactions.

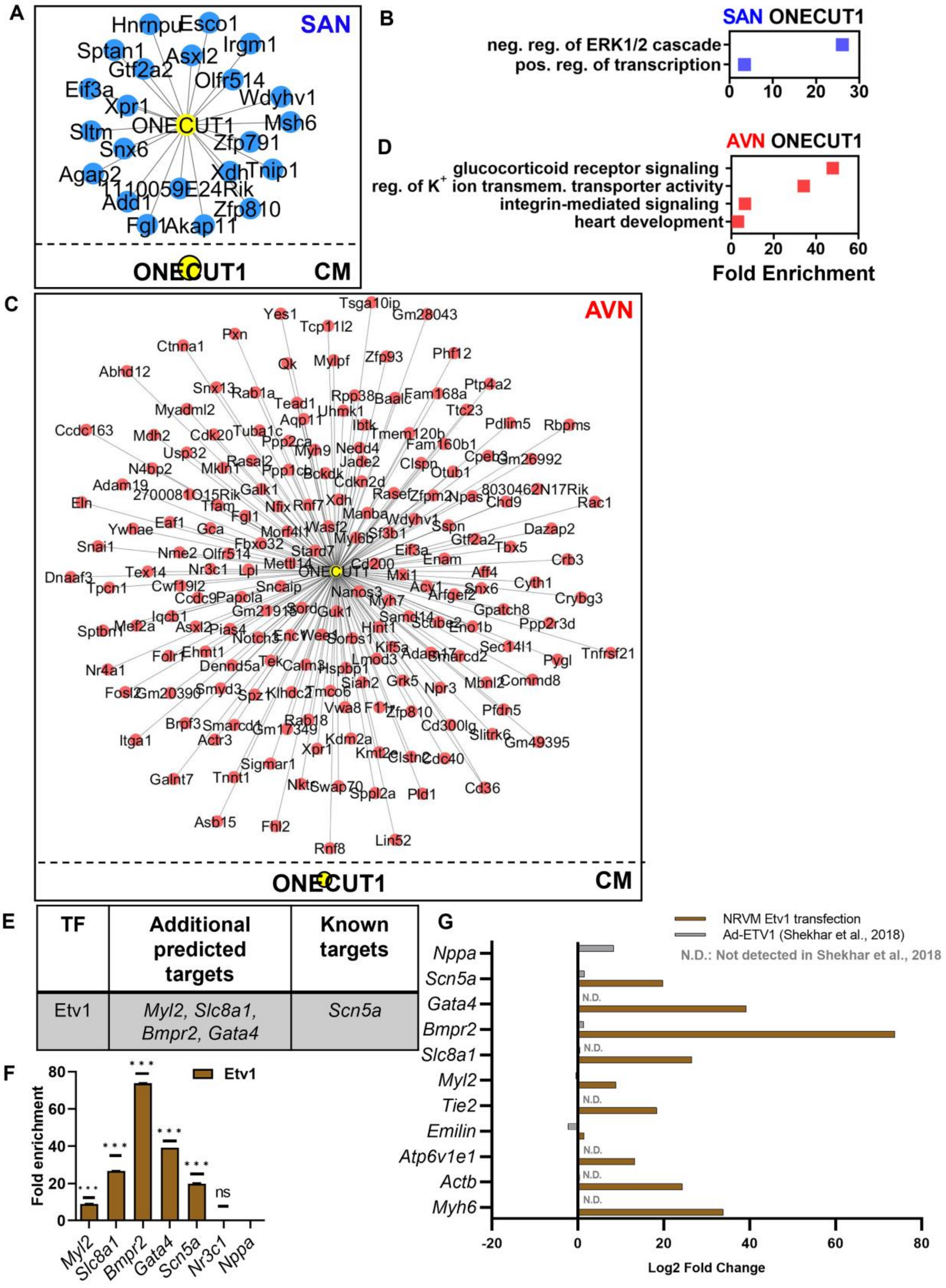

Supplemental Figure 13. Additional data relating to SAN and AVN TF sub-networks.
A) Interacting gene details for ONECUT1 sub-network in SAN and CM.
B) Enriched GO terms for ONECUT1 target genes in SAN.
C) Interacting gene details for ONECUT1 sub-network in AVN and CM.
D) Enriched GO terms for ONECUT1 target genes in AVN.
E) Table of additional predicted and known target genes for Etv1.

F) Bar graphs showing target gene induction for overexpressed Etv1. Error bars signify S.E.M. of target gene expression among 3 independent experiments. *Nppa* served as a negative control. Asterisks indicate highly significant P-value <0.000001 by Paired t-test.
G) Comparison of gene expression induced by Etv1 overexpression between the current study and Shekhar et al., 2018.

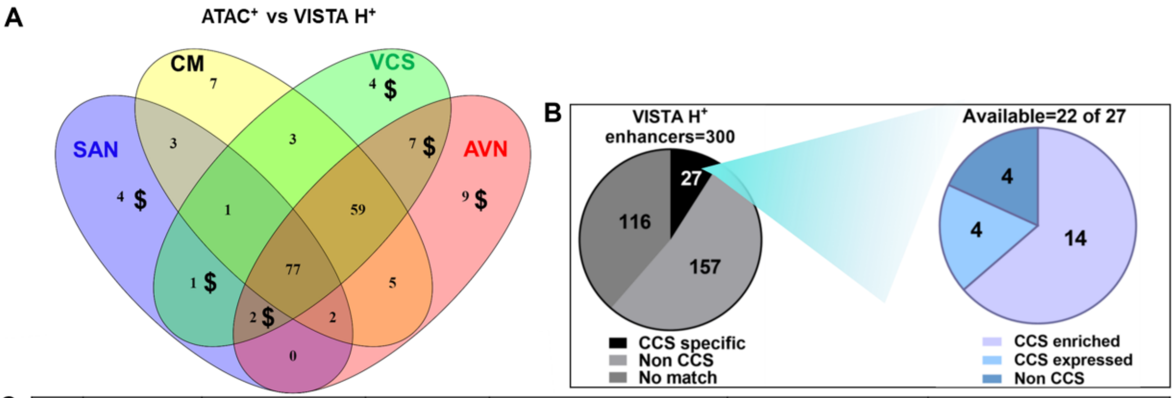

C

	VISTA ID	mm10 coordinates	CCS ATAC Dataset	LacZ localization	Bracketing genes (mouse)	Bracketing genes (human)
**1**	**mm1326**	**chr17:30548540-30552550**	**SAN**	**SAN Region**	*Gm6402-Glo1*	*BTBD9-GLO1*
**2**	**hs2384**	**chr12:8385675-8387404**	**AVN**	**AVC**		*RHOB-HS1BP3-IT1*
**3**	**hs1932**	**chr7:68010091-68013211**	**VCS**	**AVB+Trab**		*IGF1R(intragenic)*
4	mm50	chr7:142551566-142554813	AVN	AVC	*Nctc1(intragenic)*	*MRPL23-MRPL23-AS1*
5	hs1927	chr14:69828765-69831438	AVN+VCS	AVC+Trab	*Rhobtb2-Pebp4*	*PEBP4-RHOBTB2*
6	mm183	chr7:143112060-143112968	AVN+VCS	VV+ AVC+Trab	*Kcnq1(intragenic)*	
7	mm1713	chr6:93172038-93174010	AVN	VV+ SV+ AVN+ Trab	*9530026P05Rik-Magi1*	
8	mm108	chr17:7268493-7269968	SAN+AVN	Right horn of SV+Trab	*Rps6ka2(intragenic)*	*RPS6KA2-IT1-MIR1913*
9	mm1651	chr8:91966331-91968303	AVN+VCS	Trab	*Irx3-4933436C20Rik*	
10	hs1751%	chr18:67368230-67371525	SAN+VCS	AVC+Trab	*Cidea-Tubb6*	*CIDEA-TUBB6*
11	hs1002	chr3:143913930-143915288	AVN+VCS	Trab	*Gm6260-Lmo4*	*LOC100505768-LOC101927891*
12	mm78	chr12:83072395-83074413	AVN+VCS	AVC	*Rgs6 (intragenic)*	
13	hs1958	chr13:6664973-6675541	AVN	AVC+ Trab	*Pfkp-Adarb2*	*LINC00701-PFKP*
14	mm100	chr9:67040666-67043521	SAN	Sparse	*Tpm1(intragenic)*	*TPM1(intragenic)*
15	mm194	chr2:32127730-32128586	AVN	Sparse	*Ppapdc3-Prrc2b*	
16	mm1288*	chr14:55003765-55004913	AVN	Pan-cardiac	*Mir208b-Ngdn*	
17	hs2330*	chr14:55003667-55004830	AVN	Pan-cardiac		*MIR208B-NGDN*
18	hs2285	chr13:38147841-38150032	SAN	Pan-cardiac	*Riok1-Dsp*	*RIOK1-DSP*
19	mm1344	chr4:57536400-57540225	SAN+AVN	Epicardium+ IVS	*1700042G15Rik-Palm2*	*PALM2(intragenic)*
20	mm143#	chr3:104596935-104600599	AVN	4 chambers, absent in IVS	*Lrig2-Slc16a1*	
21	hs1672#	chr3:104596978-104600566	AVN	Trab		*SLC16A1-AS1-LOC100996251*
22	mm1164	chr15:56720139-56721671	SAN	AVC+Trab	*Has2os-Slc22a22*	*HAS2-AS1-ZHX2*

Supplemental Figure 14. Identification of CCS enhancers by comparison to VISTA database.
A) 4-way Venn diagram of CCS component-specific enhancers and VISTA heart (H) enhancers to identify CCS enhancer candidates (No. of mouse elements = 160; No. of human elements = 140; Total = 300). $ symbol indicates 27 CCS-enriched enhancers that were not captured by the CM-ATAC dataset.
B) Pie chart demonstrating the distribution of 300 VISTA H+ elements in comparison to our ATAC datasets (left). Amongst the 22 candidates tested, regional lacZ expression is summarized as a pie chart (right).
C) Table summarizing the 22 CCS-VISTA enhancers that underwent detailed analysis in Figure 4 and Supplementary Figure 15. % symbol for element 10 (hs1751) indicates that it has been previously described(101). * symbol for elements 16 and 17 (mm1288 and hs2330) and # symbol for elements 20 and 21 (hs1672 and mm143) indicate that they are orthologous elements from the VISTA database.

533

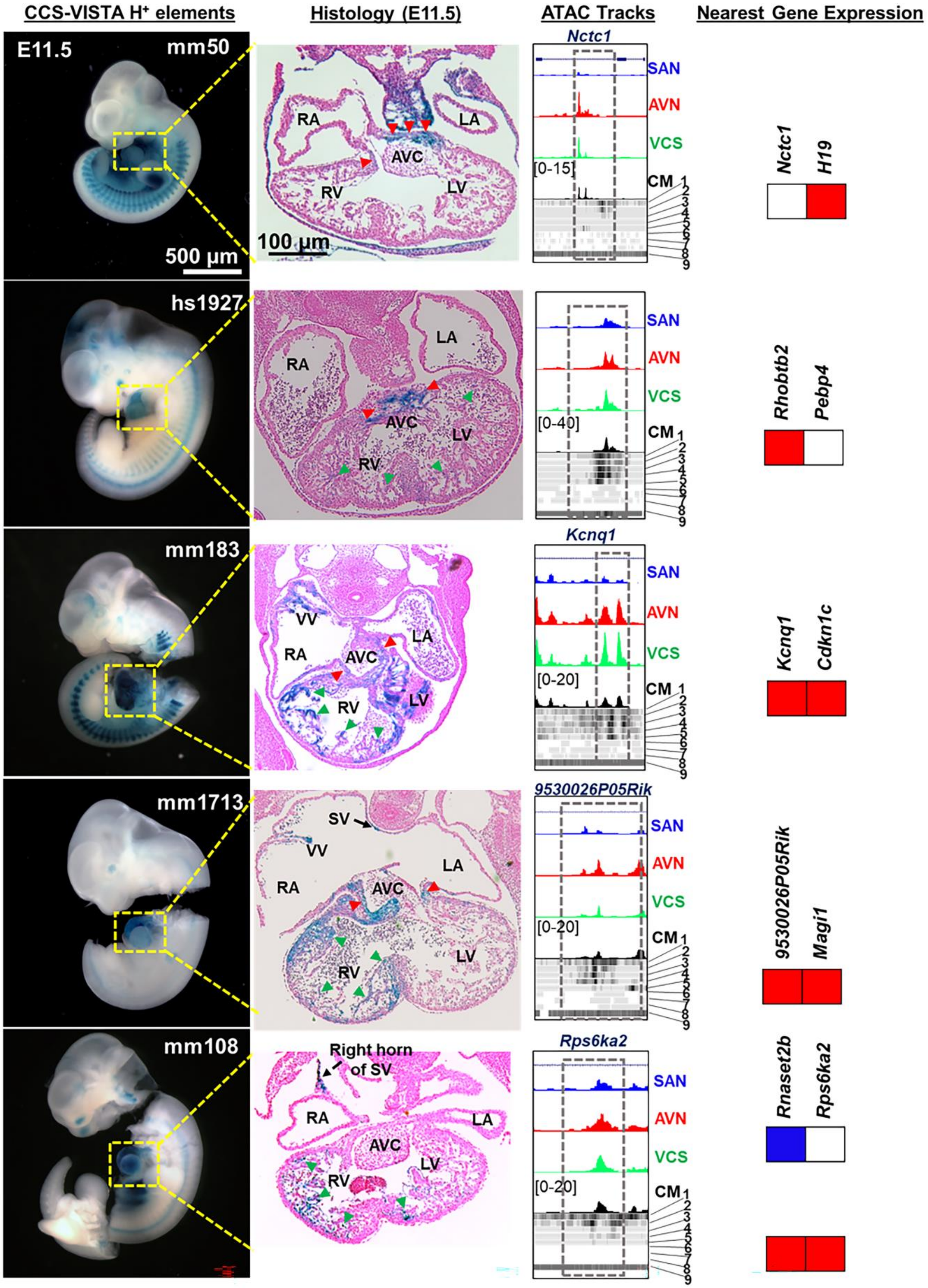

CCS-VISTA H+ elements
Histology (E11.5)
ATAC Tracks
Nearest Gene Expression
E11.5
mm50
500 µm
100 µm
RA
LA
AVC
RV
LV
Nctc1
SAN
AVN
VCS
[0-15]
CM
1
2
3
4
5
6
7
8
9
Nctc1
H19
hs1927
[0-40]
Rhobtb2
Pebp4
mm183
VV
Kcnq1
[0-20]
Kcnq1
Cdkn1c
mm1713
SV
9530026P05Rik
9530026P05Rik
Magi1
mm108
Right horn of SV
Rps6ka2
Rnaset2b
Rps6ka2

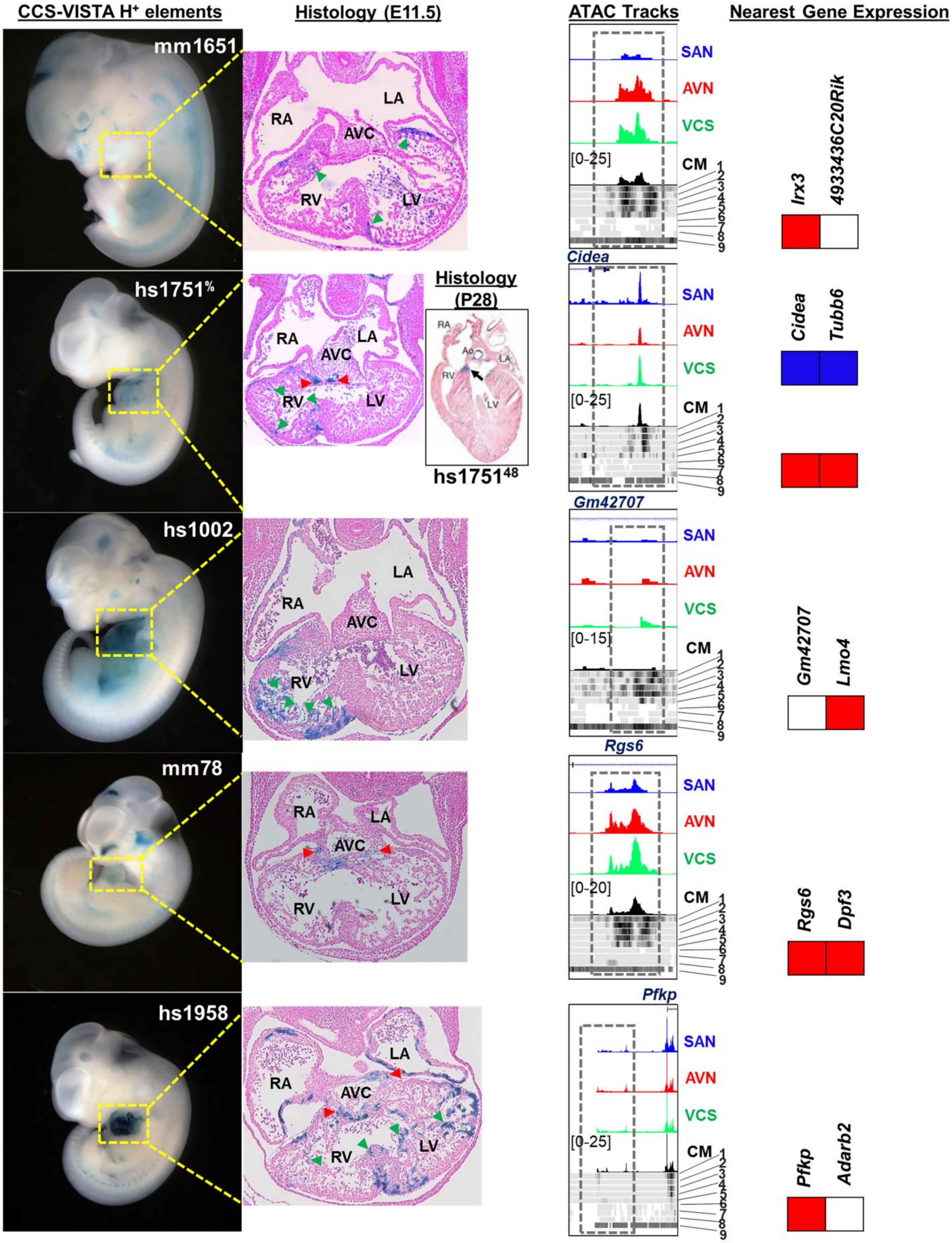
CCS-VISTA H+ elements
Histology (E11.5)
ATAC Tracks
Nearest Gene Expression
mm1651
RA
LA
AVC
RV
LV
SAN
AVN
VCS
[0-25]
CM
Irx3
4933436C20Rik
Cidea
hs1751%
Histology (P28)
hs1751[48]
Tubb6
Gm42707
hs1002
[0-15]
Lmo4
Rgs6
mm78
[0-20]
Dpf3
Pfkp
hs1958
Adarb2

534

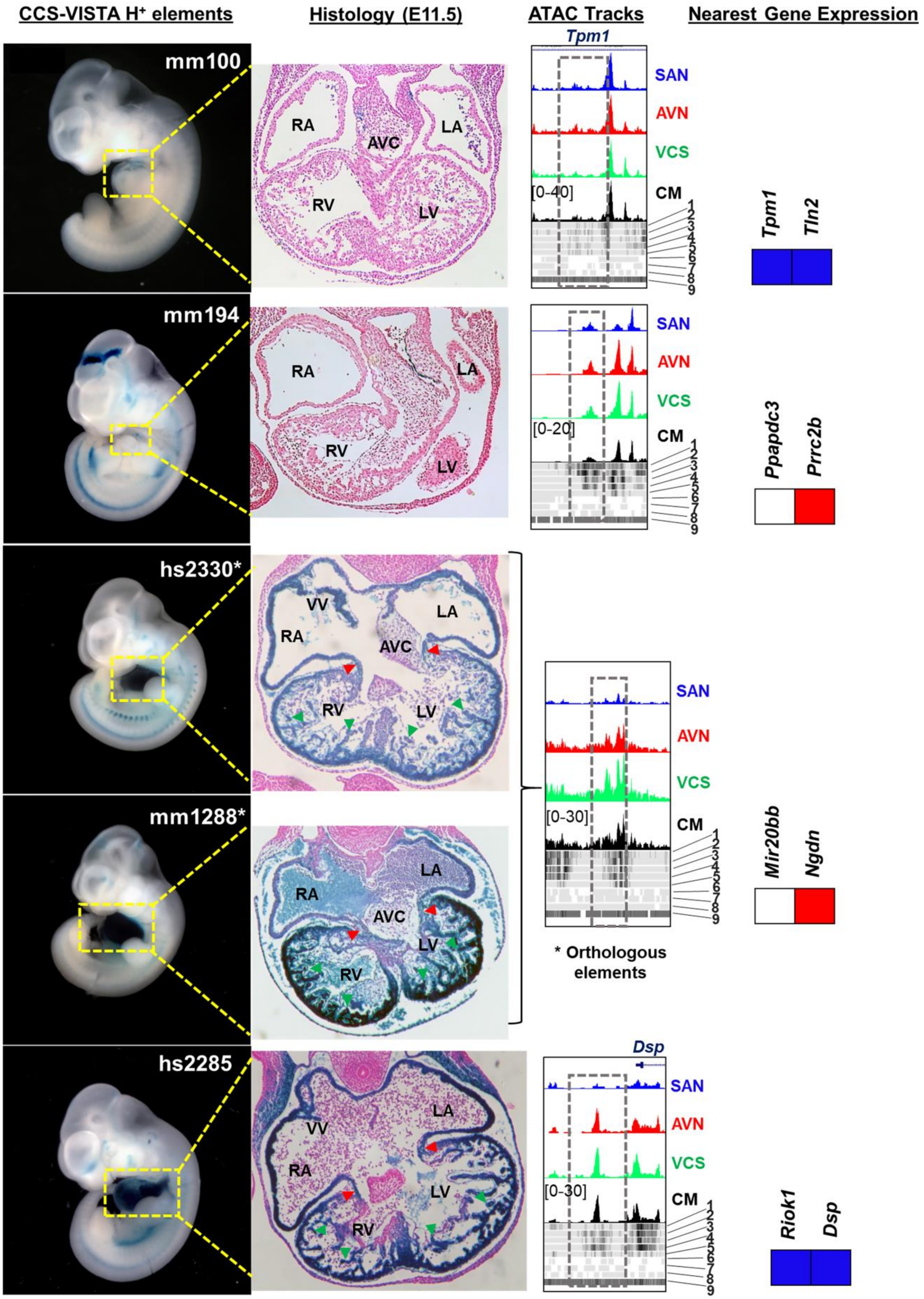
CCS-VISTA H+ elements
Histology (E11.5)
ATAC Tracks
Nearest Gene Expression
mm100
RA
AVC
LA
RV
LV
Tpm1
SAN
AVN
VCS
CM
[0-40]
1
2
3
4
5
6
7
8
9
Tpm1
Tln2
mm194
RA
LA
RV
LV
SAN
AVN
VCS
CM
[0-20]
Ppapdc3
Prrc2b
hs2330*
VV
RA
AVC
LA
RV
LV
mm1288*
RA
LA
AVC
LV
RV
SAN
AVN
VCS
CM
[0-30]
Mir20bb
Ngdn
* Orthologous elements
hs2285
LA
VV
RA
LV
RV
Dsp
SAN
AVN
VCS
CM
[0-30]
Riok1
Dsp

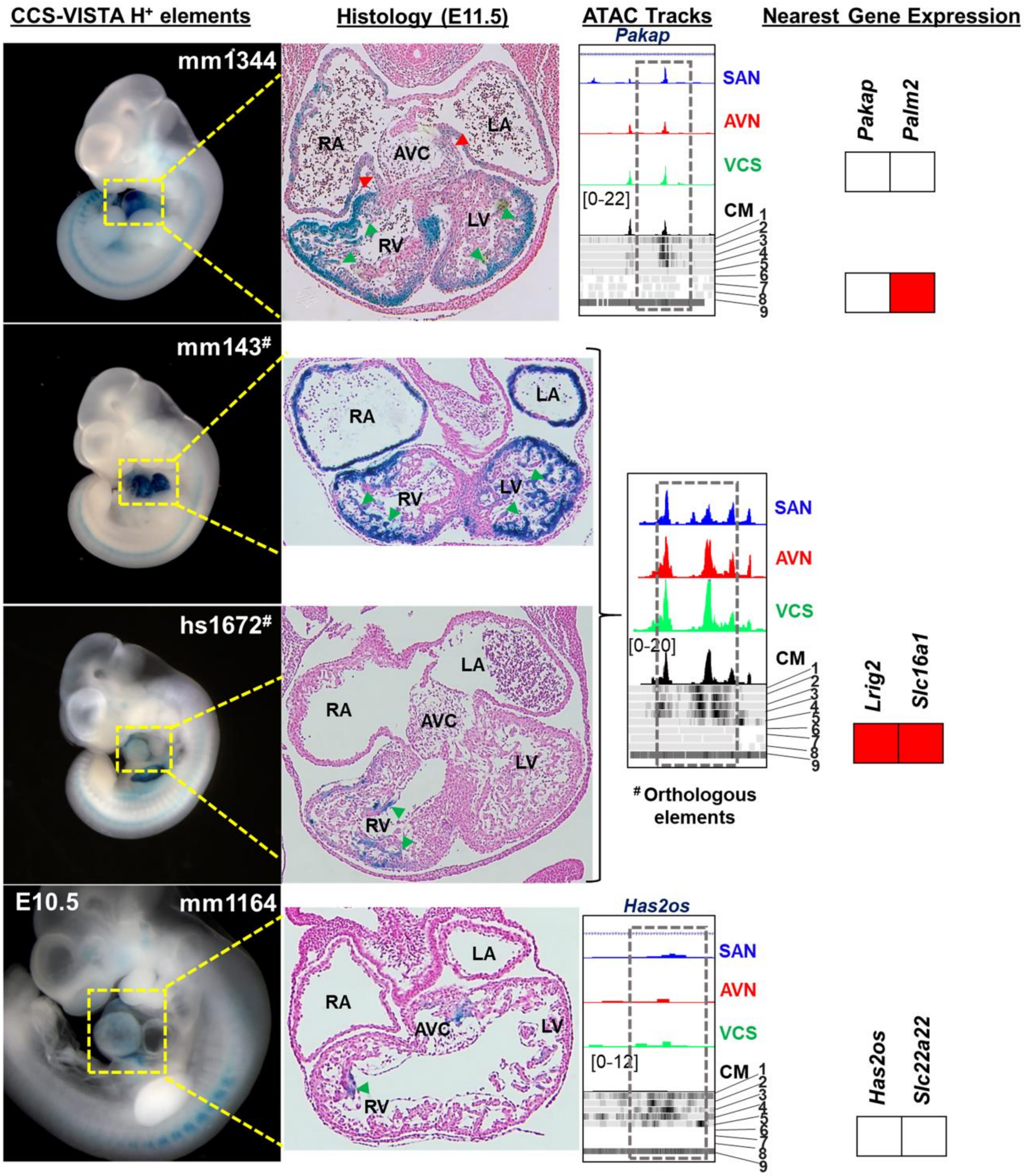

Number keys for genome browser tracks:

1. ENCODE mouse E10.5 heart-H3K27Ac ChIP-Seq
2. ENCODE mouse E12.5 heart-H3K27Ac ChIP-Seq
3. ENCODE mouse E16.5 heart-H3K27Ac ChIP-Seq
4. ENCODE mouse P0 heart-H3K27Ac ChIP-Seq
5. ENCODE mouse adult heart-H3K27Ac ChIP-Seq
6. ENCODE mouse adult brain cortex H3K27Ac ChIP-Seq
7. ENCODE mouse adult cerebellum H3K27Ac ChIP-Seq
8. ENCODE mouse adult spleen H3K27Ac ChIP-Seq
9. Placental conservation

Supplemental Figure 15. Comprehensive histological assessment of candidate CCS enhancers.

Whole mount images and histological sections of E11.5 VISTA enhancer transgenic embryos to localize LacZ expression in specific CCS components of the heart (Scale as shown); P28 CCS-ATAC browser tracks for each VISTA enhancer to show signal enrichment in respective CCS components. Gene expression for nearest-neighbor genes for each enhancer element are shown with solid blue box (from SAN scRNA-Req) or solid red box (from AVCS scRNA-Seq), which indicate transcript expression within >15% of SAN or AVCS cells, respectively. P28 lacZ expression in the heart is shown for hs1751(101) with distinct labeling of the AV Bundle region (black arrow).

Genome Browser tracks: grey dotted boxes highlight the location of specific enhancer elements. For each browser track, normalized ATAC read counts are shown on the y-axis with genome position on the x-axis.

Key for genome browser tracks: ENCODE mouse H-H3K27Ac ChIP-Seq (1: E10.5, 2: E12.5, 3: E16.5, 4: P0, 5: Adult); ENCODE mouse adult non-cardiac tissues H3K27Ac ChIP-Seq (6: Cortex, 7: Cerebellum, 8: Spleen); 9: Placental conservation.

Blue arrowheads indicate region of sinus horn myocardium. Red arrowheads indicate AVC myocardium and cushion mesenchyme. Green arrowheads point at developing Purkinje fibers (also known as endocardial trabeculations).

RA, right atrium; LA, left atrium; RV, right ventricle; LV, left ventricle; VV, venous valves; SV, sinus venosus.

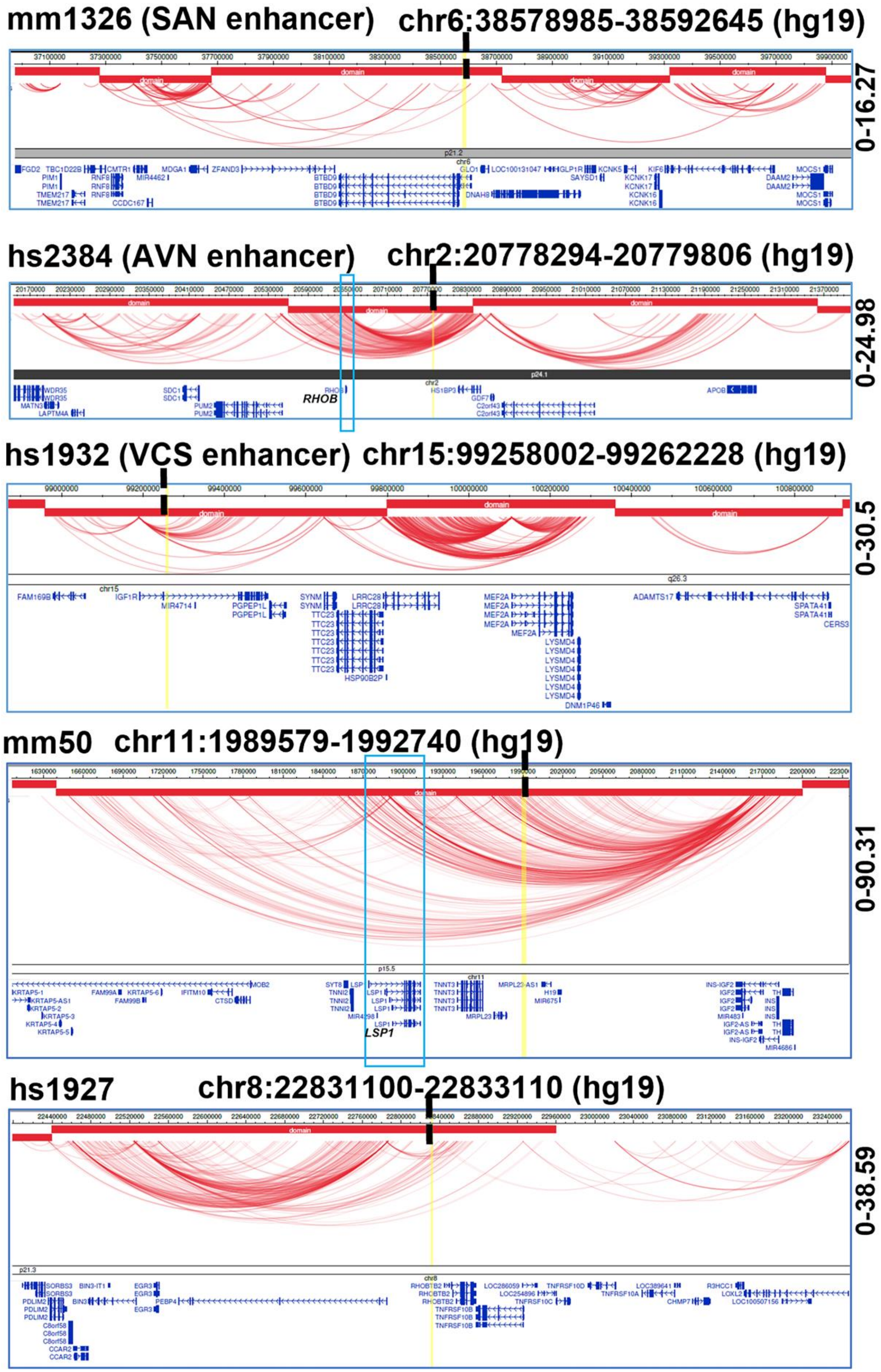
mm1326 (SAN enhancer) chr6:38578985-38592645 (hg19)
0-16.27
hs2384 (AVN enhancer) chr2:20778294-20779806 (hg19)
RHOB
0-24.98
hs1932 (VCS enhancer) chr15:99258002-99262228 (hg19)
0-30.5
mm50 chr11:1989579-1992740 (hg19)
LSP1
0-90.31
hs1927 chr8:22831100-22833110 (hg19)
0-38.59

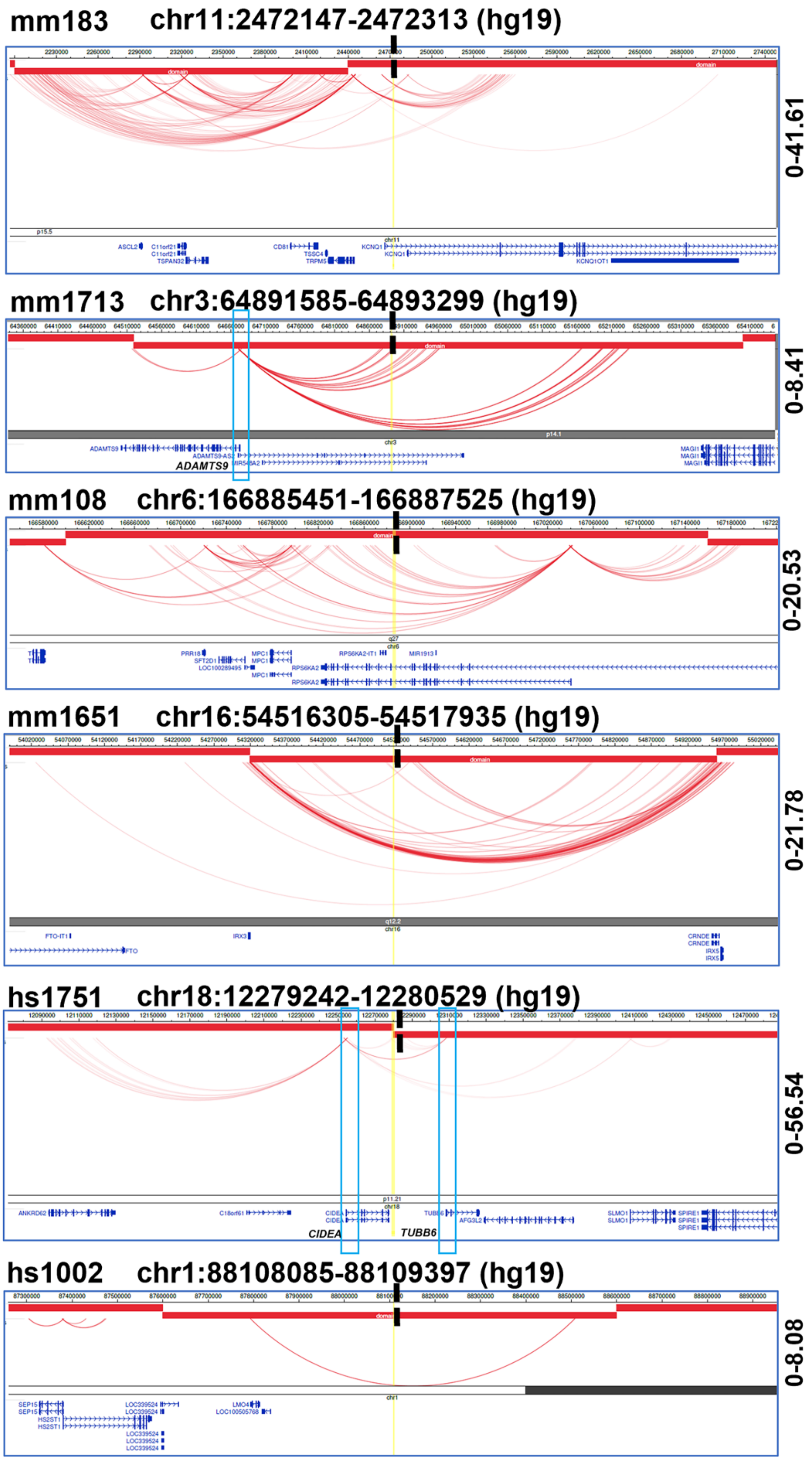

mm183
chr11:2472147-2472313 (hg19)
0-41.61
mm1713
chr3:64891585-64893299 (hg19)
0-8.41
ADAMTS9
mm108
chr6:166885451-166887525 (hg19)
0-20.53
mm1651
chr16:54516305-54517935 (hg19)
0-21.78
hs1751
chr18:12279242-12280529 (hg19)
0-56.54
CIDEA
TUBB6
hs1002
chr1:88108085-88109397 (hg19)
0-8.08

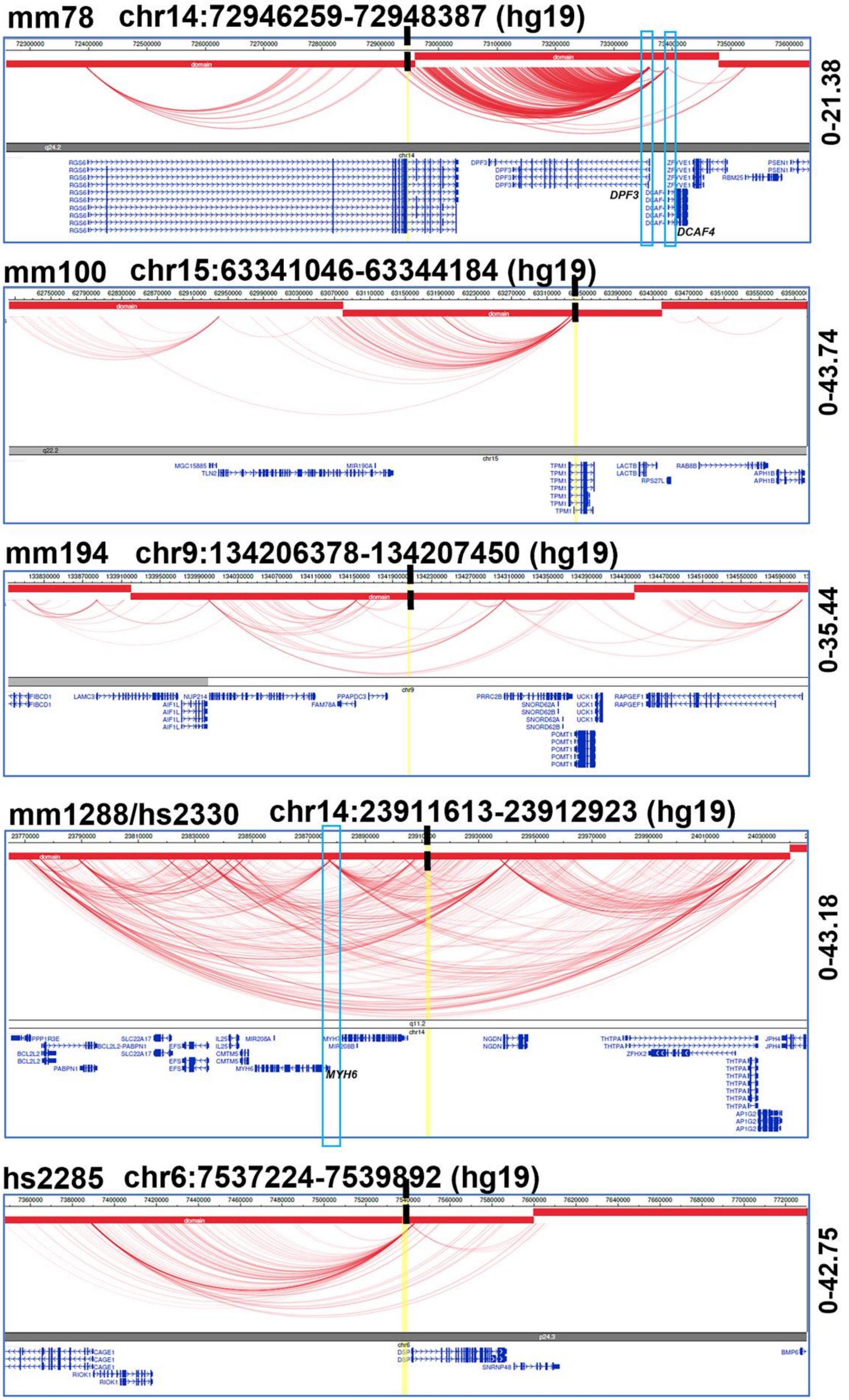
mm78 chr14:72946259-72948387 (hg19)
0-21.38
DPF3
DCAF4
mm100 chr15:63341046-63344184 (hg19)
0-43.74
mm194 chr9:134206378-134207450 (hg19)
0-35.44
mm1288/hs2330 chr14:23911613-23912923 (hg19)
0-43.18
MYH6
hs2285 chr6:7537224-7539892 (hg19)
0-42.75

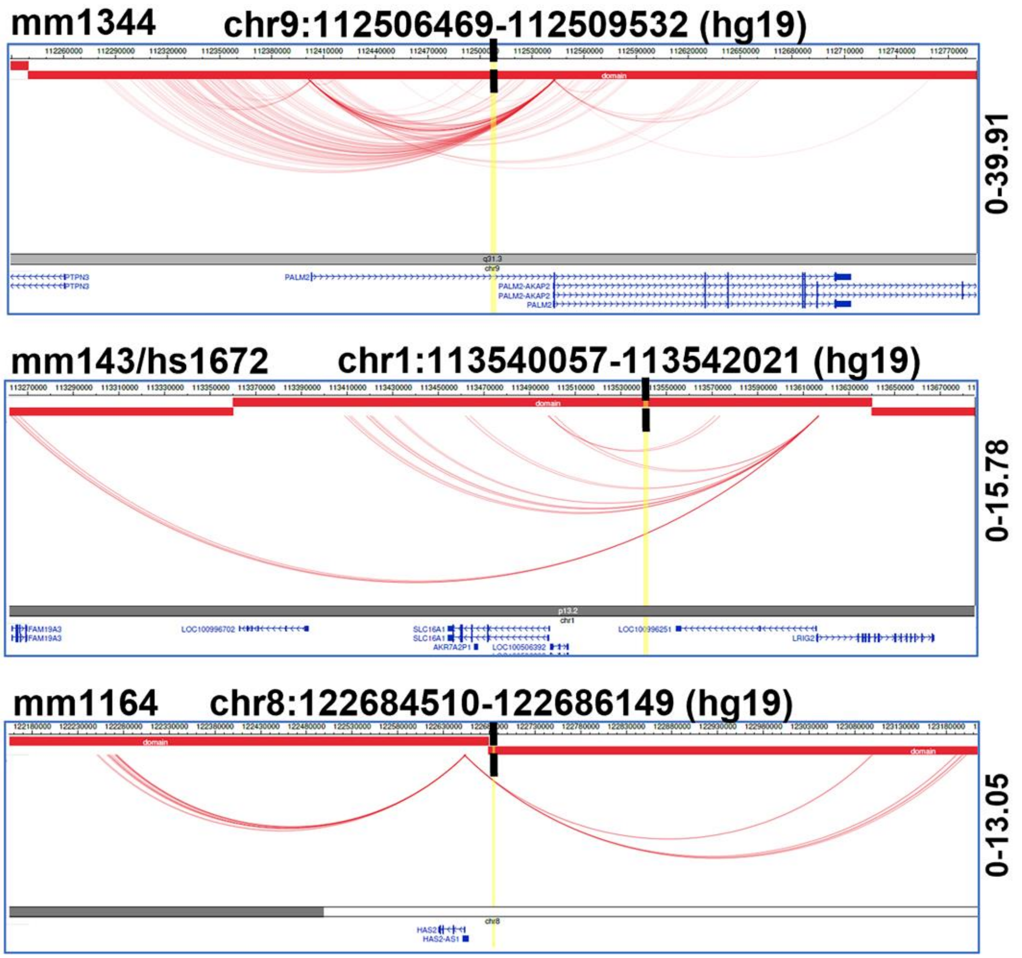

Supplemental Figure 16. Promoter-Capture Hi-C (PCHi-C) maps for candidate enhancers. Genome browser views for PCHi-C maps from human iPSC-derived CMs were obtained from the WashU EpiGenome Browser**.** Each regulatory element is labeled by its VISTA number along with its genomic coordinates mapped to hg19. For each browser track, the element is highlighted by a yellow line, and genomic contacts are shown as red lines. Clear enhancer-promoter connections identified by PCHi-C are highlighted by blue boxes (hs2384: *RHOB*; mm50: *LSP1*; mm1713: *ADAMTS19*; hs1751: *CIDEA*/*TUBB6*; mm1288/hs2330: *MYH6*). The elements are ordered as in Figures 4, S14, and S15.

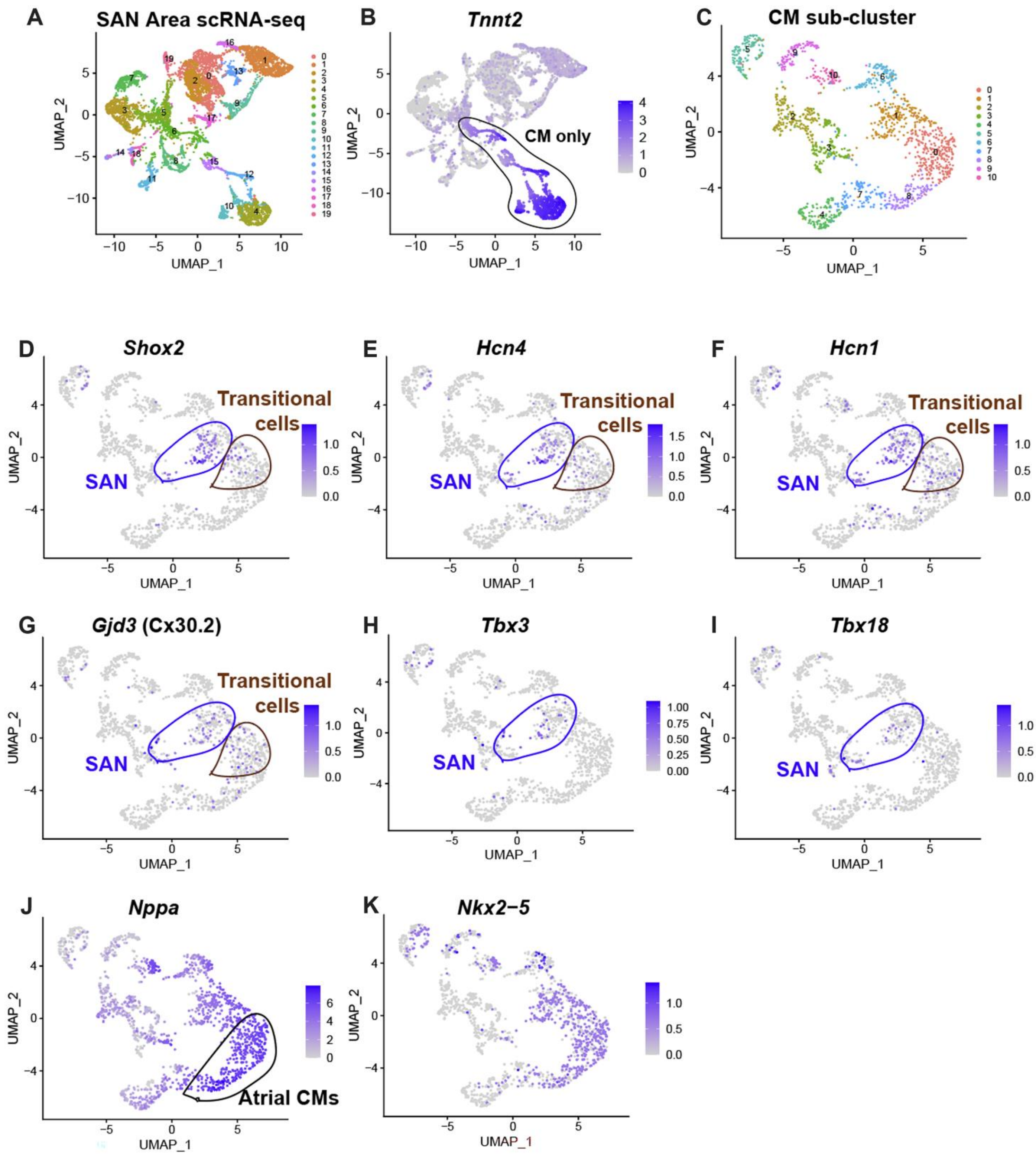

Supplemental Figure 17. Supervised cluster assignment for SAN single cell atlas.
A) UMAP plot of scRNA-Seq derived from micro-dissected P4 mouse SAN region after data filtering and normalization. Color code indicates individual cell clusters.
B) Same UMAP plot as in (A) with overlay of *Tnnt2* expression to distinguish CMs and non-CMs.
C) CMs were re-clustered and displayed as a secondary UMAP plot. Color code indicates individual cell clusters.
D) UMAP plot of SAN CMs with overlay of *Shox2* expression.

E) UMAP plot of SAN CMs with overlay of *Hcn4* expression.
F) UMAP plot of SAN CMs with overlay of *Hcn1* expression.
G) UMAP plot of SAN CMs with overlay of *Gjd3* expression.
H) UMAP plot of SAN CMs with overlay of *Tbx3* expression.
I) UMAP plot of SAN CMs with overlay of *Tbx18* expression.
J) UMAP plot of SAN CMs with overlay of *Nppa* expression.
K) UMAP plot of SAN CMs with overlay of *Nkx2-5* expression.
Blue region outlines cells of the compact SAN(102); brown region outlines transitional cells(102); black region outlines atrial cells.

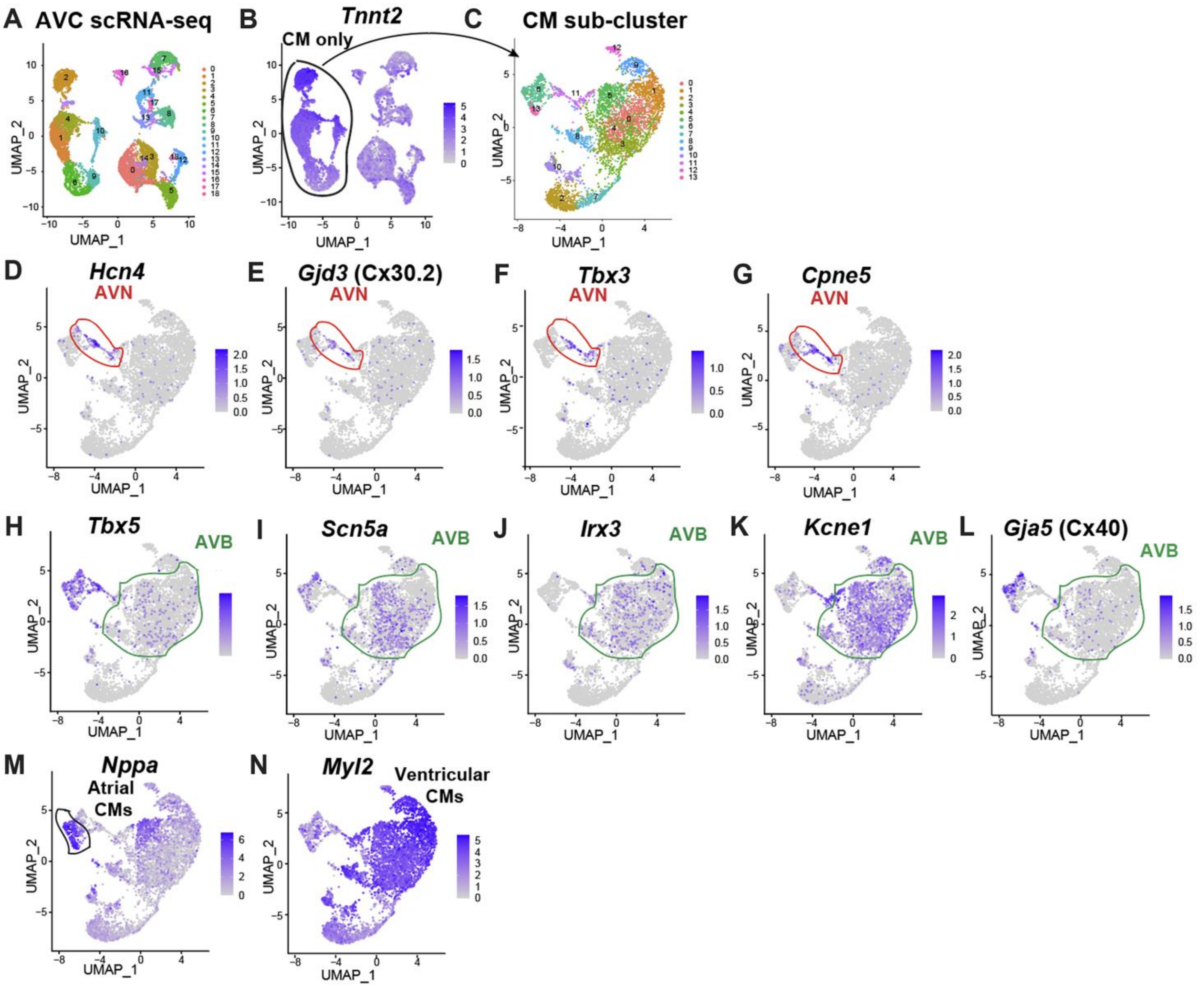

Supplemental Figure 18. Supervised cluster assignment for AVCS single cell atlas.
A) UMAP plot of scRNA-Seq derived from micro-dissected P0 mouse AVC region after data filtering and normalization. Color code indicates individual cell clusters.
B) Same UMAP plot as in (A) with overlay of *Tnnt2* expression to distinguish CMs and non-CMs.
C) CMs were re-clustered and displayed as a UMAP plot. Color code indicates individual cell clusters.
D) UMAP plot of AVC CMs with overlay of *Hcn4* expression.
E) UMAP plot of AVC CMs with overlay of *Gjd3* expression.
F) UMAP plot of AVC CMs with overlay of *Tbx3* expression.
G) UMAP plot of AVC CMs with overlay of *Cpne5* expression.
H) UMAP plot of AVC CMs with overlay of *Tbx5* expression.
I) UMAP plot of AVC CMs with overlay of *Scn5a* expression.
J) UMAP plot of AVC CMs with overlay of *Irx3* expression.
K) UMAP plot of AVC CMs with overlay of *Kcne1* expression.
L) UMAP plot of AVC CMs with overlay of *Gja5* expression.
M) UMAP plot of AVC CMs with overlay of *Nppa* expression.
N) UMAP plot of AVC CMs with overlay of *Myl2* expression.
Red region outlines cells of the compact AVN(98, 102); green region outlines AVB cells(98); black region outlines atrial cells.

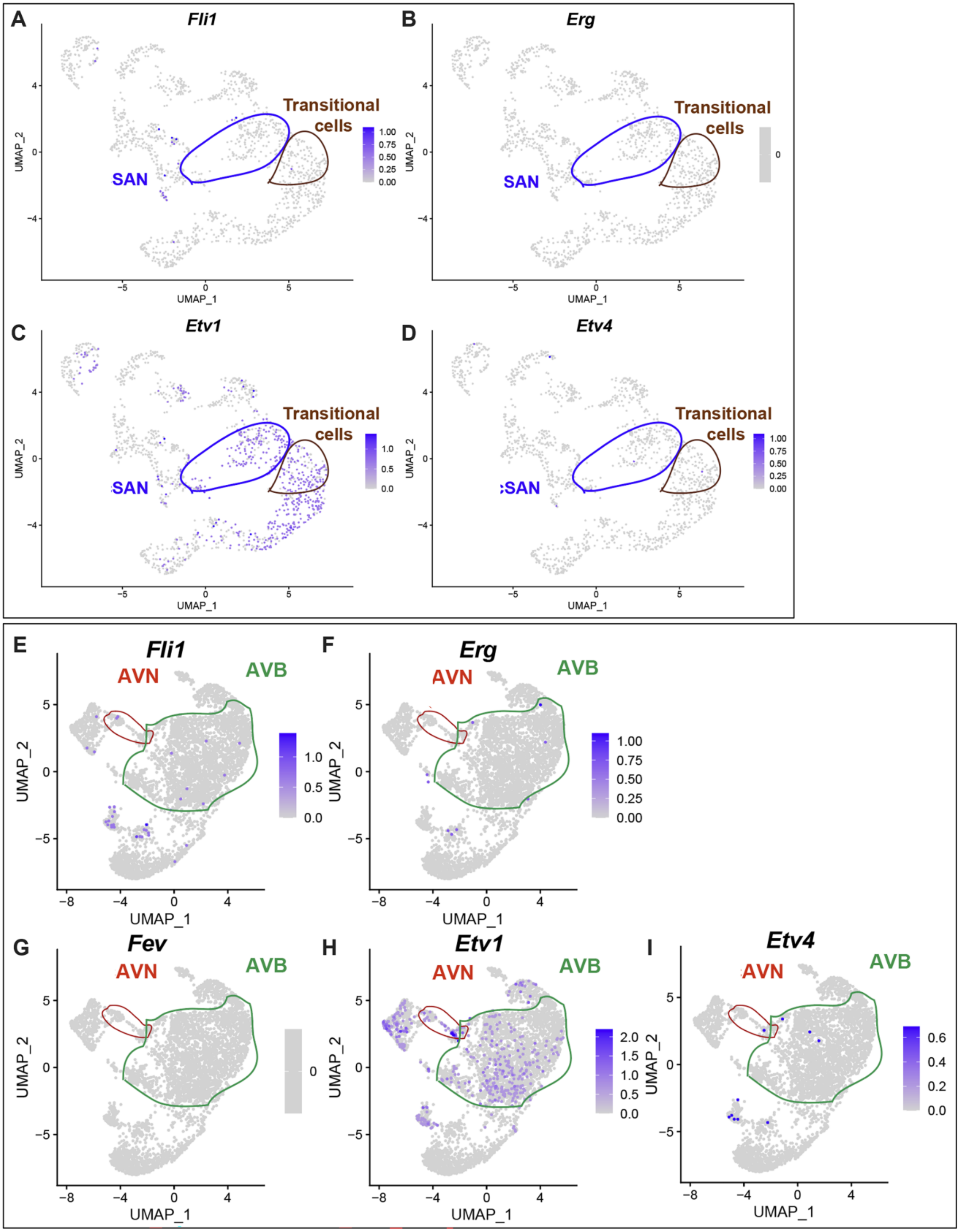

Supplemental Figure 19. CCS expression patterns of ETS family TFs.

A-D) UMAP plot of SAN CMs with overlaid expression of ETS family TFs (*Fli1, Erg, Etv1, and Etv4*).

E-I) UMAP plot of AVC CMs with overlaid expression of ETS family TFs (*Fli1, Erg, Fev, Etv1, and Etv4*).

Target genes with transcript expression in >15% cells of interest are displayed.

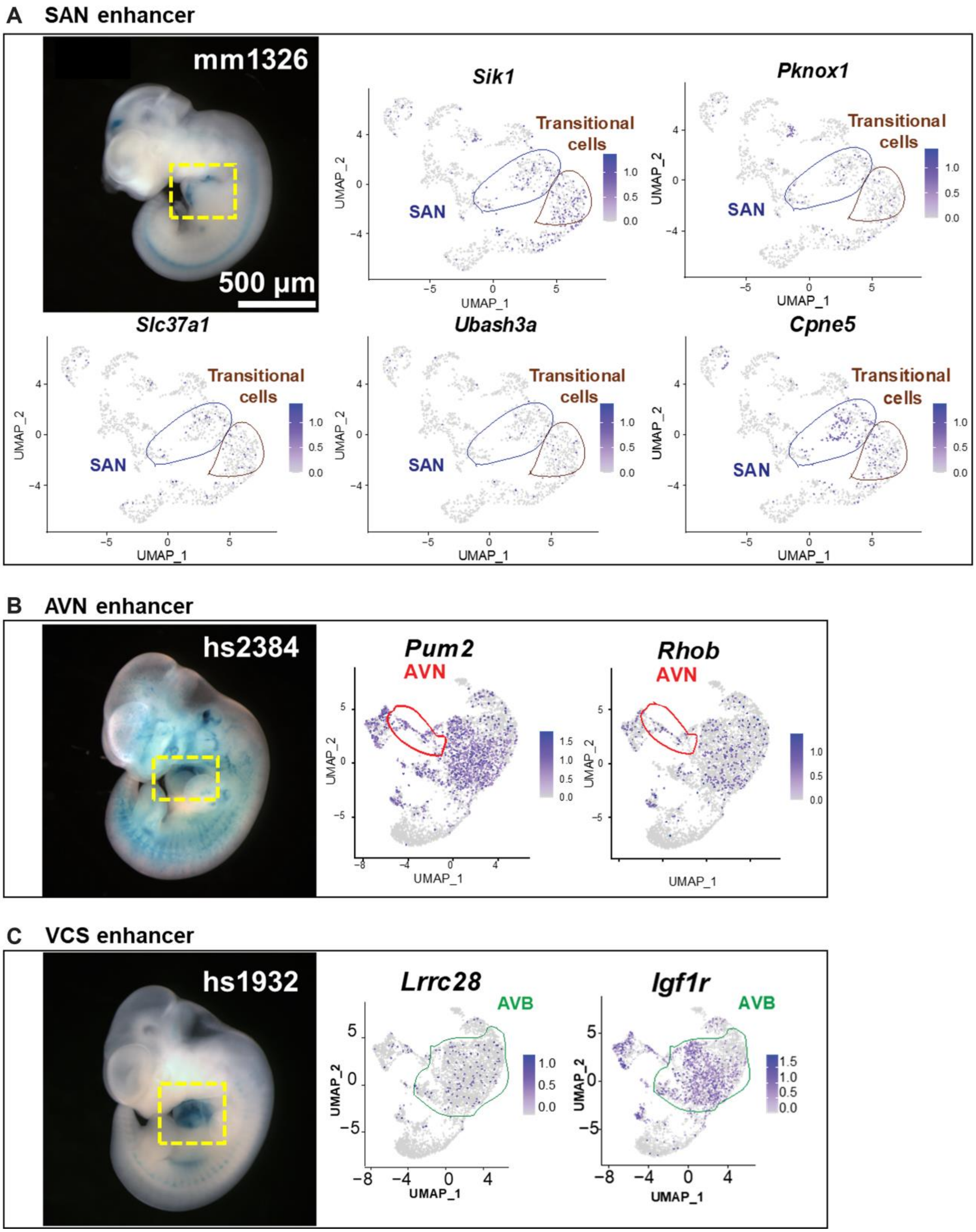

Supplemental Figure 20. Expression of candidate CCS enhancer target genes.
A) Whole-mount embryo image for SAN enhancer element mm1326 and UMAP plot of SAN CMs with overlaid expression of additional candidate target genes within ±500kb of the VISTA element. B) Whole-mount embryo image for AVN enhancer element hs2384 and UMAP plot of AVCS CMs with overlaid expression of additional candidate target genes within ±500kb of the VISTA element. C) Whole-mount embryo image for VCS enhancer element hs1932 and UMAP plot of AVCS CMs with overlaid expression of additional candidate target genes within ±500kb of the VISTA element. Target genes with transcript expression in >15% cells of interest are displayed.

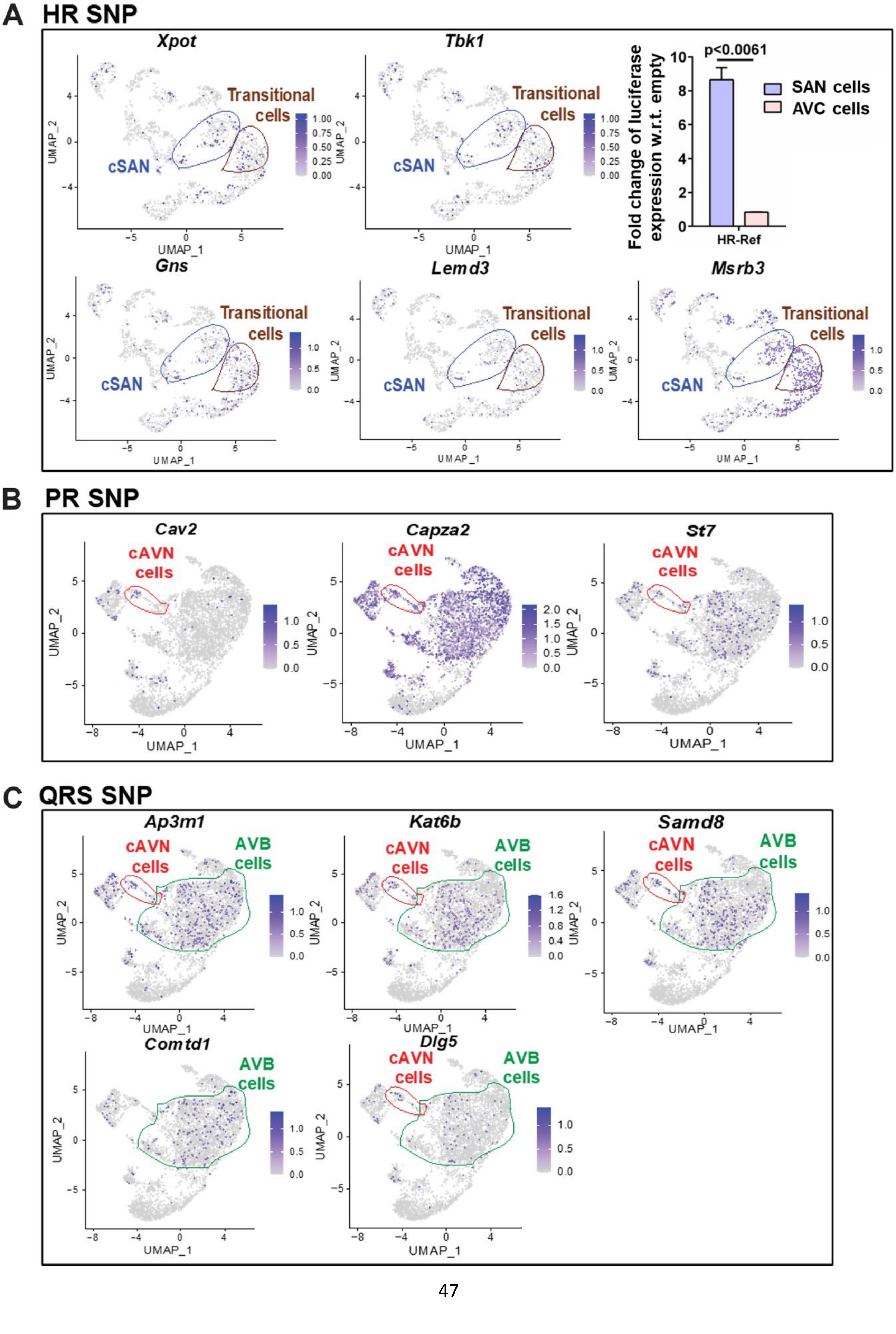
A HR SNP
Xpot
Tbk1
Gns
Lemd3
Msrb3
Transitional cells
cSAN
UMAP_1
UMAP_2
Fold change of luciferase expression w.r.t. empty
p<0.0061
SAN cells
AVC cells
HR-Ref
B PR SNP
Cav2
Capza2
St7
cAVN cells
C QRS SNP
Ap3m1
Kat6b
Samd8
Comtd1
Dlg5
AVB cells

D Q-T SNP

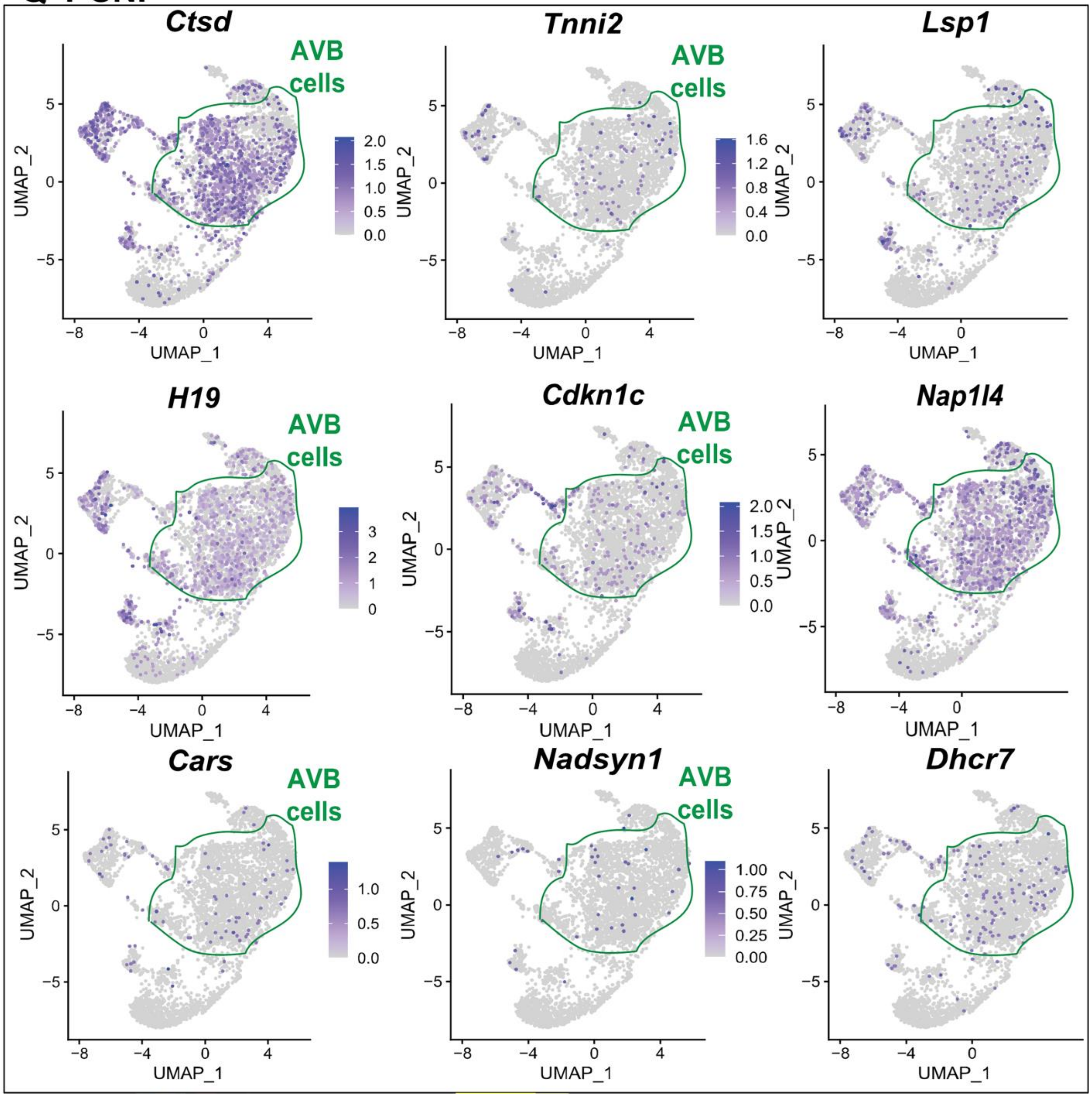

Supplemental Figure 21. Expression of candidate CCS GWAS SNP enhancer target genes. A) UMAP plot of SAN CMs with overlaid expression of additional candidate target genes (*Xpot*, *Tbx1*, *Gns*, *Lemd3*, and *Msrb1*) within ±500kb of rs867400 (HR SNP). Bar graph represents relative luciferase units for the element underlying rs867400 relative to empty-luciferase construct in primary mouse SAN and AVC cells. P-value by two-tailed Paired t-test is shown. Error bars signify S.E.M. of luciferase expression between 2 independent experiments.
B) UMAP plot of AVCS CMs with overlaid expression of additional candidate target genes (*Cav2, Capza2,* and *St7*) within ±500kb of rs3807989 (PR SNP).
C) UMAP plot of AVCS CMs with overlaid expression of additional candidate target genes (*Ap3m1, Kat6b, Samd8, Comtd1,* and *Dlg5*) within ±500kb of rs12764182 (QRS SNP).

D) UMAP plot of AVCS CMs with overlaid expression of additional candidate target genes (*Ctsd, Tnni2, Lsp1, H19, Cdkn1c, Nap1l4, Cars, Nadsyn1,* and *Dhcr7*) within ±500kb of rs2074238 (QT SNP).
Color-coded boundaries denote cell clusters of interest. Target genes with transcript expression in >15% cells of interest are displayed.

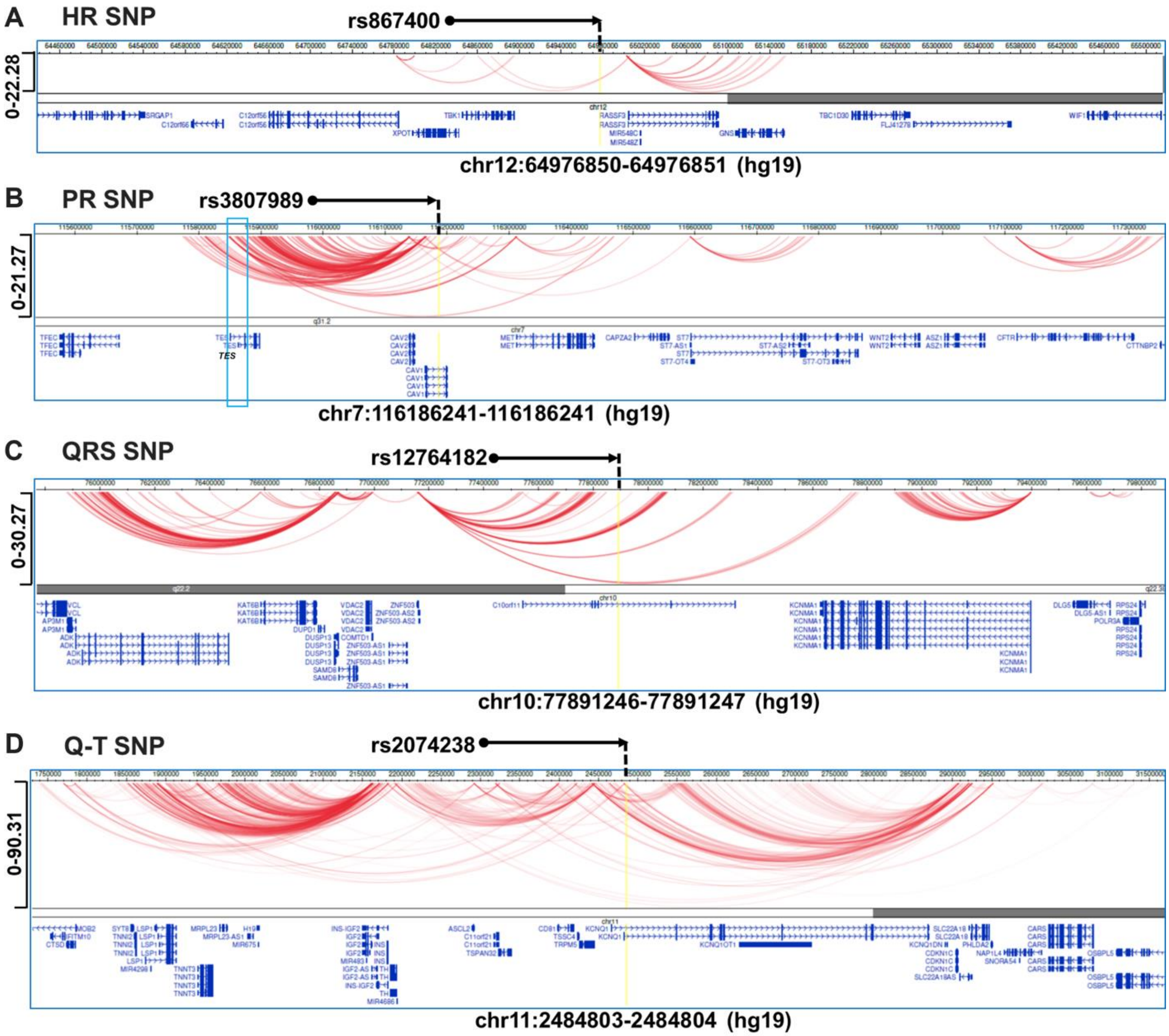

Supplemental Figure 22. PCHi-C maps for candidate enhancers underlying EKG SNPs. Genome browser views for PCHi-C maps from human iPSC-derived CMs were obtained from the WashU EpiGenome Browser**.** Each candidate SNP is labeled by its rsID number along with the genomic coordinates mapped to hg19. For each browser track, the location of the SNP is highlighted by a yellow line, and genomic contacts are shown as red lines. A clear SNP-promoter connections identified by PCHi-C is highlighted by a blue box (rs3807989: *TES*). The elements are ordered as in Figure 5.

Supplementary References

76. Corces MR, Trevino AE, Hamilton EG, Greenside PG, Sinnott-Armstrong NA, Vesuna S, Satpathy AT, Rubin AJ, Montine KS, Wu B, et al. An improved ATAC-seq protocol reduces background and enables interrogation of frozen tissues. *Nat Methods.* 2017;14(10):959-62.
77. Bhattacharyya S, Sathe AA, Bhakta M, Xing C, and Munshi NV. PAN-INTACT enables direct isolation of lineage-specific nuclei from fibrous tissues. *PLoS One.* 2019;14(4):e0214677.
78. Langmead B, and Salzberg SL. Fast gapped-read alignment with Bowtie 2. *Nat Methods.* 2012;9(4):357-9.
79. Bysani M, Agren R, Davegardh C, Volkov P, Ronn T, Unneberg P, Bacos K, and Ling C. ATAC-seq reveals alterations in open chromatin in pancreatic islets from subjects with type 2 diabetes. *Scientific reports.* 2019;9(1):7785.
80. Heinz S, Benner C, Spann N, Bertolino E, Lin YC, Laslo P, Cheng JX, Murre C, Singh H, and Glass CK. Simple combinations of lineage-determining transcription factors prime cis-regulatory elements required for macrophage and B cell identities. *Molecular cell.* 2010;38(4):576-89.
81. Yu G, Wang LG, and He QY. ChIPseeker: an R/Bioconductor package for ChIP peak annotation, comparison and visualization. *Bioinformatics.* 2015;31(14):2382-3.
82. Mouse EC, Stamatoyannopoulos JA, Snyder M, Hardison R, Ren B, Gingeras T, Gilbert DM, Groudine M, Bender M, Kaul R, et al. An encyclopedia of mouse DNA elements (Mouse ENCODE). *Genome biology.* 2012;13(8):418.
83. Quinlan AR. BEDTools: The Swiss-Army Tool for Genome Feature Analysis. *Curr Protoc Bioinformatics.* 2014;47(11 2 1-34.
84. Trapnell C, Pachter L, and Salzberg SL. TopHat: discovering splice junctions with RNA-Seq. *Bioinformatics.* 2009;25(9):1105-11.
85. Preissl S, Schwaderer M, Raulf A, Hesse M, Gruning BA, Kobele C, Backofen R, Fleischmann BK, Hein L, and Gilsbach R. Deciphering the Epigenetic Code of Cardiac Myocyte Transcription. *Circ Res.* 2015;117(5):413-23.
86. Love MI, Huber W, and Anders S. Moderated estimation of fold change and dispersion for RNA-seq data with DESeq2. *Genome biology.* 2014;15(12):550.
87. Subramanian A, Tamayo P, Mootha VK, Mukherjee S, Ebert BL, Gillette MA, Paulovich A, Pomeroy SL, Golub TR, Lander ES, et al. Gene set enrichment analysis: a knowledge-based approach for interpreting genome-wide expression profiles. *Proc Natl Acad Sci U S A.* 2005;102(43):15545-50.
88. van Eif VWW, Stefanovic S, van Duijvenboden K, Bakker M, Wakker V, de Gier-de Vries C, Zaffran S, Verkerk AO, Boukens BJ, and Christoffels VM. Transcriptome analysis of mouse and human sinoatrial node cells reveals a conserved genetic program. *Development.* 2019;146(8).
89. Horsthuis T, Buermans HP, Brons JF, Verkerk AO, Bakker ML, Wakker V, Clout DE, Moorman AF, t Hoen PA, and Christoffels VM. Gene expression profiling of the forming atrioventricular node using a novel tbx3-based node-specific transgenic reporter. *Circ Res.* 2009;105(1):61-9.
90. Shekhar A, Lin X, Liu FY, Zhang J, Mo H, Bastarache L, Denny JC, Cox NJ, Delmar M, Roden DM, et al. Transcription factor ETV1 is essential for rapid conduction in the heart. *J Clin Invest.* 2016;126(12):4444-59.
91. McLean CY, Bristor D, Hiller M, Clarke SL, Schaar BT, Lowe CB, Wenger AM, and Bejerano G. GREAT improves functional interpretation of cis-regulatory regions. *Nat Biotechnol.* 2010;28(5):495-501.

92. Rendeiro AF, Schmidl C, Strefford JC, Walewska R, Davis Z, Farlik M, Oscier D, and Bock C. Chromatin accessibility maps of chronic lymphocytic leukaemia identify subtype-specific epigenome signatures and transcription regulatory networks. *Nat Commun.* 2016;7(11938.

93. Fornes O, Castro-Mondragon JA, Khan A, van der Lee R, Zhang X, Richmond PA, Modi BP, Correard S, Gheorghe M, Baranasic D, et al. JASPAR 2020: update of the open-access database of transcription factor binding profiles. *Nucleic Acids Res.* 2020;48(D1):D87-D92.

94. . VISTA Browser. https://enhancer.lbl.gov.

95. Buniello A, MacArthur JAL, Cerezo M, Harris LW, Hayhurst J, Malangone C, McMahon A, Morales J, Mountjoy E, Sollis E, et al. The NHGRI-EBI GWAS Catalog of published genome-wide association studies, targeted arrays and summary statistics 2019. *Nucleic Acids Res.* 2019;47(D1):D1005-D12.

96. Sebastian A, and Contreras-Moreira B. footprintDB: a database of transcription factors with annotated cis elements and binding interfaces. *Bioinformatics.* 2014;30(2):258-65.

97. Zhou X, Li D, Zhang B, Lowdon RF, Rockweiler NB, Sears RL, Madden PA, Smirnov I, Costello JF, and Wang T. Epigenomic annotation of genetic variants using the Roadmap Epigenome Browser. *Nat Biotechnol.* 2015;33(4):345-6.

98. Bhattacharyya S, Duan J, Wang L, Li B, Bhakta M, Fernandez-Perez A, Hon GC, and Munshi NV. Using Gjd3-CreEGFP mice to examine atrioventricular node morphology and composition. *Scientific reports.* 2019;9(1):2106.

99. van den Boogaard M, Wong LYE, Tessadori F, Bakker ML, Dreizehnter LK, Wakker V, Bezzina CR, 't Hoen PAC, Bakkers J, Barnett P, et al. Genetic variation in T-box binding element functionally affects SCN5A/SCN10A enhancer. *Journal of Clinical Investigation.* 2012;122(7):2519-30.

100. Galang G, Mandla R, Ruan H, Jung C, Sinha T, Stone NR, Wu RS, Mannion BJ, Allu PKR, Chang K, et al. ATAC-Seq Reveals an Isl1 Enhancer That Regulates Sinoatrial Node Development and Function. *Circ Res.* 2020;127(12):1502-18.

101. May D, Blow MJ, Kaplan T, McCulley DJ, Jensen BC, Akiyama JA, Holt A, Plajzer-Frick I, Shoukry M, Wright C, et al. Large-scale discovery of enhancers from human heart tissue. *Nat Genet.* 2012;44(1):89-93.

102. Goodyer WR, Beyersdorf BM, Paik DT, Tian L, Li G, Buikema JW, Chirikian O, Choi S, Venkatraman S, Adams EL, et al. Transcriptomic Profiling of the Developing Cardiac Conduction System at Single-Cell Resolution. *Circ Res.* 2019;125(4):379-97.
